# Metabolomic and Lipidomic Biomarkers for Premalignant Liver Disease Diagnosis and Therapy

**DOI:** 10.3390/metabo10020050

**Published:** 2020-01-28

**Authors:** Diren Beyoğlu, Jeffrey R. Idle

**Affiliations:** Arthur G. Zupko’s Division of Systems Pharmacology and Pharmacogenomics, Arnold & Marie Schwartz College of Pharmacy and Health Sciences, Long Island University, 75 Dekalb Avenue, Brooklyn, NY 11201, USA; diren.beyoglu@liu.edu

**Keywords:** metabolomics, lipidomics, biomarker, premalignant, alcoholic liver disease, cholestasis, fibrosis, cirrhosis, NAFL, NASH

## Abstract

In recent years, there has been a plethora of attempts to discover biomarkers that are more reliable than α-fetoprotein for the early prediction and prognosis of hepatocellular carcinoma (HCC). Efforts have involved such fields as genomics, transcriptomics, epigenetics, microRNA, exosomes, proteomics, glycoproteomics, and metabolomics. HCC arises against a background of inflammation, steatosis, and cirrhosis, due mainly to hepatic insults caused by alcohol abuse, hepatitis B and C virus infection, adiposity, and diabetes. Metabolomics offers an opportunity, without recourse to liver biopsy, to discover biomarkers for premalignant liver disease, thereby alerting the potential of impending HCC. We have reviewed metabolomic studies in alcoholic liver disease (ALD), cholestasis, fibrosis, cirrhosis, nonalcoholic fatty liver (NAFL), and nonalcoholic steatohepatitis (NASH). Specificity was our major criterion in proposing clinical evaluation of indole-3-lactic acid, phenyllactic acid, *N*-lauroylglycine, decatrienoate, *N*-acetyltaurine for ALD, urinary sulfated bile acids for cholestasis, cervonoyl ethanolamide for fibrosis, 16α-hydroxyestrone for cirrhosis, and the pattern of acyl carnitines for NAFL and NASH. These examples derive from a large body of published metabolomic observations in various liver diseases in adults, adolescents, and children, together with animal models. Many other options have been tabulated. Metabolomic biomarkers for premalignant liver disease may help reduce the incidence of HCC.

## 1. The Need for Biomarkers of Premalignant Liver Disease

Hepatocellular carcinoma (HCC) and intrahepatic cholangiocarcinoma (ICC) are the commonest types of primary liver cancer, and their combined incidence ranks among the highest cancer rates in the world [1]. HCC in particular is a major health problem, with an annual death rate in excess of 500,000 worldwide [2]. HCC in the United States, which comprises 75% of all primary liver cancers [3], has been attributed primarily to a number of infectious and lifestyle causes. The principal attributable factors among these are alcohol (32.0% in males, 30.7% in females), adiposity (26.6% in males, 15.6% in females), hepatitis C virus (HCV) infection (17.5% overall), smoking (9.0% in males, 8.0% in females), diabetes (6.9% in males, 5.5% in females), and hepatitis B virus (HBV) infection (5.3% overall). In contrast, in China, HBV (53.8% overall) is the principal cause, with adiposity a relatively minor contributor (7.2% in males, 4.2% in females) [4]. These causative factors produce insults to the liver that include inflammation, steatosis, and fibrosis, all of which can progress through various stages, in particular cirrhosis, that can eventually lead to HCC. In recent years, there have been multiple attempts to develop predictive biomarkers of HCC, but many of these have involved the study of HCC cases themselves. Understanding the progression from hepatic insult through premalignant stages to HCC would seem to be the most fruitful means of predicting the development of HCC in susceptible individuals. In this review, we examine the investigations into key premalignant stages of HCC and ICC that have employed metabolomics both in patients and in animal models. In particular, we have focused on the metabolomics of alcoholic liver disease (ALD), cholestasis, fibrosis, cirrhosis, nonalcoholic fatty liver (steatosis, NAFL), and nonalcoholic steatohepatitis (NASH). In each case, we evaluated whether the experimental data provide sufficient grounds, especially in terms of specificity, to warrant further development of clinical biomarkers of hepatic premalignancy. Additionally, we considered only metabolites that were upregulated as potential biomarkers for the aforementioned premalignant liver diseases. The references cited in this review were culled from PubMed searches with keywords metabolomics OR metabonomics AND the various disease entities, such as alcoholic liver disease. Some references also arose from the bibliographies cited by publications found in these initial searches. 

## 2. Hepatic Metabolism

The human body comprises around 34 trillion cells of which ca. 240 billion (0.7%) make up the approximately 1.5 kg of healthy liver, the largest solid organ and the biggest gland in the body. Of the roughly 20,000 human protein-coding genes, 60% are transcribed in the liver, many of which are not expressed in any other tissue [5]. Studies in mice using single cell transcriptomics revealed that about half of all hepatocyte genes were expressed in a zonal manner, supporting the concept that different liver regions have diverse metabolic functions. This was interpreted as being due to variable microenvironments attributable to gradients of oxygen, nutrients, and hormones [6]. Metabolic reactions that are specific to the liver include de novo synthesis and secretion of the primary bile acids glycocholate, taurocholate, glycochenodeoxycholate, and taurochenodeoxycholate, together with ornithine degradation. Overall, the liver is the most metabolically-active tissue, followed by adipose tissue and skeletal muscle [5]. Parenchymal hepatocytes comprise up to 85% of the liver volume, with sinusoidal endothelial cells, perisinusoidal stellate cells and phagocytic Kupffer cells, with intrahepatic lymphocytes making up the rest. Strong evidence suggests that different hepatic cell types possess variable gene expression profiles [6,7,8]. The liver is therefore highly heterogeneous in both gene expression and metabolic function. Assignment of metabolic function to discrete hepatic regions based upon in vivo observations alone is extremely challenging, since metabolic phenotypes vary between cell types and also across the liver. The role of in vitro studies in this regard will be increasingly important as aids to the interpretation of in vivo metabolic phenotyping. For example, laser capture microdissection has been employed as an adjunct to genomic, transcriptomic, and proteomic analyses of liver diseases [9], but so far, rarely for metabolic profiling of liver tissue.

## 3. Metabolomics—The What, the How, and the Why

It is two decades since Jeremy Nicholson and colleagues introduced the concept of metabonomics, with the promise of biomarker discovery from changes in metabolite profiles that result from constitutional differences such as disease or genetics or from exogenous challenges due to drug administration or exposure to toxicants [10]. The initial protocols based upon high-resolution proton nuclear magnetic resonance spectroscopy (^1^H NMR) of body fluids have been supplemented by an array of additional technologies, based mostly on mass spectrometry (MS), which have infiltrated virtually every branch of biology and medicine. The literature currently stands at virtually 30,000 PubMed citations with almost 6000 in 2019 alone. The identification and quantitation of all metabolites in a given organism or biofluid was at first seen as a realistic goal [11]. However, as the biochemical complexity and analytical shortcomings came more into focus, global metabolite quantitation was abandoned, and more realistic definitions emerged, such as, “metabolomics studies the low molecular weight metabolites [e.g., <1.5 kDa] found in cells and organisms, usually through the analysis of plasma/serum, urine or cell culture medium using mainly MS or NMR technologies” [12]. There has also been some confusion regarding the use of the terms “metabolomics” and “metabonomics.” Although it has been stated that the difference in terms is not a technical one, and that the terminologies are often used interchangeably [13], almost without exception, metabonomics published reports were conducted using NMR rather than MS. Other commonly-used phrases include untargeted and targeted metabolic phenotyping. Untargeted metabolomics is commonly conducted by first separating the biological analytes that have a large range of physicochemical properties using ultraperformance liquid chromatography (UPLC) with either reversed phase (RP) and/or hydrophobic interaction chromatography (HILIC) columns [14]. Interfaced by electrospray ionization (ESI) in either positive (ESI+) and/or negative (ESI-) mode, the UPLC eluate is analyzed by quadrupole time-of-flight mass spectrometry (QTOFMS). This may yield in excess of 5000 ions in each ionization mode, which should not be interpreted as 5000 biological constituents, as many of these features correspond to adducts, dimers, multiply charged species, and fragment ions formed in the electrosprayer. In targeted metabolomics, specific metabolites, for example amino acids or acyl carnitines, are quantitated using stable isotope labeled standards [15]. This is frequently conducted using tandem mass spectrometry, often with a triple quadrupole mass spectrometer (TQMS), rather than a QTOFMS. Another common technology used in metabolomics is gas chromatography-mass spectrometry (GC-MS). This has the benefit of a high confidence in metabolite identification, albeit for a small number of metabolites and a lower throughput than UPLC-QTOFMS. The technologies available for metabolomic analysis have recently been reviewed in detail [16]. 

In a typical metabolomics experiment, two or more groups of samples are investigated. These could be biofluids from a patient group and age- and sex-matched healthy controls, genetically-modified mice and their wild-type (WT) controls, and persons or experimental animals that have been administered a drug, specific diet, or with some other lifestyle variable (e.g. smoking or particular occupation), compared with a suitable control group. Analysis of the biofluids, usually urine and/or serum/plasma, by MS- or NMR-based methods produces a data table that must first be preprocessed (normalization, scaling, peak picking) prior to multivariate data analysis (MDA). It is first prudent to conduct unsupervised MDA, for example, with principal components analysis (PCA), which reveals the internal structure of the dataset, the principal components of variance, and the existence of any outliers. A number of presentations of the data are common, including the scores plot (with one data point for each sample) and the loadings plot, which for MS methods show the ions responsible for the distribution of samples in the scores plot. If each sample group analyzed clusters and separates from the other group(s), then this leads to supervised analyses such as partial least squares-discriminant analysis (PLS-DA) and orthogonal PLS-DA (OPLS-DA). Unless at least a partial separation of scores was observed in the PCA analysis, there is a danger that the data could be overmodeled using these supervised analyses. The literature is replete with examples of this. The generated loadings plots can be used with various software packages that assist in the identification of metabolites that differ significantly between the test groups. The reader is directed to specific reviews in this area [17,18,19].

Various estimates of distinct human metabolomes have been reported that were derived using multiple analytical platforms to gain maximum metabolite coverage. The human cerebrospinal fluid metabolome (308 metabolites) [20], the human serum metabolome (4229 “highly probable” metabolites) [21], the human urine metabolome (2651 “confirmed” metabolites) [22], and the human fecal metabolome (>6000 identified metabolites) [23] have all been described. The culmination of these efforts is the human metabolome database (HMDB 4.0) that comprises 114,100 total metabolites that encompass “the complete collection of small molecules found in the human body including peptides, lipids, amino acids, nucleic acids, carbohydrates, organic acids, biogenic amines, vitamins, minerals, food additives, drugs, cosmetics, contaminants, pollutants, and just about any other chemical that humans ingest, metabolize, catabolize or come into contact with” [24]. This still may be the tip of the iceberg. It has been estimated that humans are probably exposed to some 1–3 million discrete chemicals in their lifetimes [11] of which >25,000 have already been described in the diet [25]. 

The lipidome refers to the total number of lipid species present in a cell, tissue, organ, organism, or biofluid such as plasma. Although there is overlap with the human metabolome, the human lipidome is expected to be highly complex due in great part to the varying chain lengths and degrees of unsaturation, together with structural isomerism. As of January 2018, there were more than 40,000 lipid structures listed in the LIPID MAPS database [26]. Our conservative estimate is that the human lipidome is made up of at least 100,000 discrete lipid entities. 

Based on the foregoing evidence, it is likely that a human metabolome that includes the lipidome may have some 200,000 members. As stated above, there are thought to be ~20,000 protein coding human genes, although the exact number is yet to be determined. The total number of cellular proteins (proteome) may be 16,000–17,000, similar to the total number of mRNA transcripts (transcriptome) obtained by untargeted RNA sequencing (RNA-seq) [27]. In addition, the existence of a human core proteome of 10,000–12,000 ubiquitously expressed proteins has been postulated, whose primary function is the general control and maintenance of cells [28]. Many of these are enzymes, and therefore contribute to the human metabolome, either through the metabolism of a single specific metabolite or pair of metabolites, such as lactate dehydrogenase or in a pleiotropic fashion, such as the thousands of potential metabolites produced by the human cytochromes P450 [29]. Nevertheless, it has been estimated that there are 1–2 million “protein entities” that are expressed in a cell at a given time as a result of posttranslational modifications (PTMs), such as acetylation, phosphorylation, and glycosylation [30]. To study mechanisms of liver disease through the lens of untargeted proteomics would be an extremely demanding task. However, targeted proteomics in the form of specific protein biomarkers in plasma or serum has a long history. This is because of the availability of commercial antibodies against virtually every protein and this forms the basis of convenient quantitative immunoassays such as ELISA. 

**Figure 1:**
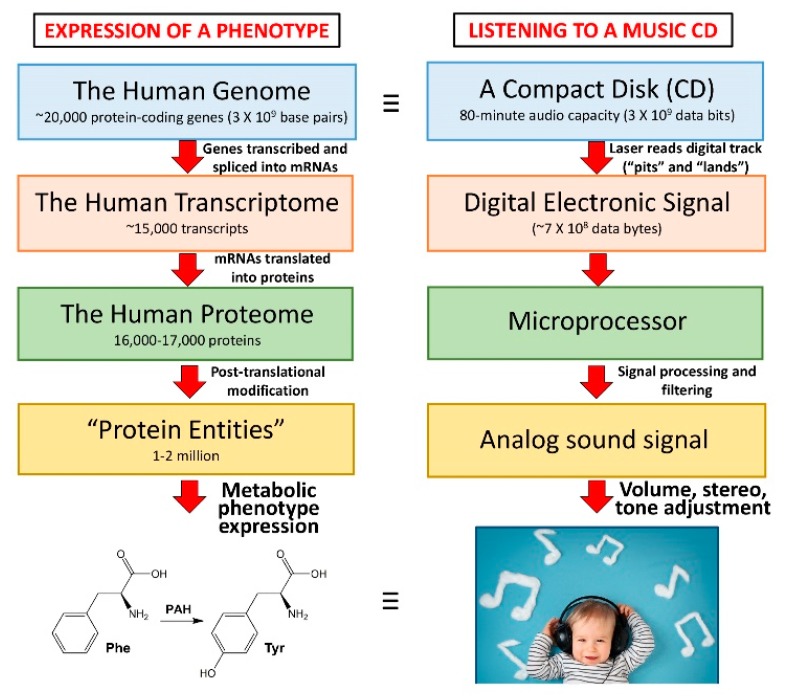
The flow of genetic information vs. digital music data flow.

The expression of phenotypes, including metabolic phenotypes, from a genomic sequence that is transcribed, spliced, and translated to protein with potential post-translational modifications, is analogous to the information flow involved when listening to a music compact disk or some other digital music format. In the former case, the genome is analogous to the compact disk itself, which without the apparatus for converting it to sound, is simply a digital storage system (Figure 1). This is why the metabolic phenotype is more revealing of the status of a cell, tissue, or organism than a genetic sequence, because it more resembles the musical experience rather than analyzing the so-called pits and lands (Figure 1) on a CD.

In terms of generating new knowledge regarding the liver, metabolomics has for some years offered this opportunity. Because the liver is the seat of much of the body’s metabolic processes, the systemic measurement of metabolites that originate in the liver should provide clear signposts to liver wellbeing or disease. This alone justifies the inclusion of metabolomic protocols in the study of hepatic pathogenesis. As we will demonstrate below, a plethora of such studies has already been reported, but the picture is still not in focus. We will seek to highlight the potential biomarkers that can be determined through metabolomics and that point most directly to disease mechanisms. We will discuss below the shortcomings of the current trend of identifying metabolomic biomarkers as risk factors for liver disease.

## 4. Biomarkers—The Good, the Bad, and the Ugly

A biomarker has been characterized as “a defined characteristic that is measured as an indicator of normal biological processes, pathogenic processes, or responses to an exposure or intervention, including therapeutic interventions.” It has also been emphasized that biomarkers can be medical measurements, including physiological measurements, blood tests, molecular analyses of biopsies, genetic or metabolic data, and measurements from images [31]. Blood pressure and blood glucose are commonly determined biomarkers of both pathogenic processes and therapeutic interventions. Neither of these biomarkers point to mechanisms of either disease or therapeutic response. Measurement of the pressure of various parts of the arterial circulation was initiated in the mid-18th century by the English clergyman Stephen Hales [32]. The testing of the color, smell, and taste of urine as indicators of disease goes back at least as far as the ancient Greeks, with diagnostic ‘urine charts’ dating from the Middle Ages [13]. Determination of blood glucose developed relatively recently and as a substitute to urine taste as a diagnostic biomarker for diabetes [33]. 

The first metabolic biomarkers that indicated disease mechanisms are contained in the remarkable work of Sir Archibald Garrod (1857–1936) who coined the phrases “inborn errors of metabolism” [34] and “chemical individuality” [35] in the early part of the 20th century. Garrod contended that four diseases, i.e., alkaptonuria, albinism, cystinuria, and pentosuria, were Mendelian autosomal recessive traits, therefore pointing to genetic mechanisms for each. Moreover, he recognized that increased urinary homogentisic acid (HGA; known then as “alkapton acid”) in newborn babies with alkaptonuria that stained their diapers black could be further increased by the oral administration of tyrosine or a diet rich in proteins containing aromatic amino acids such as tyrosine and phenylalanine [36]. This led Garrod to propose an impairment in the aromatic ring opening of aromatic amino acids as the mechanism of alkaptonuria. This flew in the face of the contemporaneous “germ theory of disease” that focused on external rather than inborn causes of disease, and maintained that alkaptonuria resulted from a gastrointestinal infection. These ideas hindered the acceptance of Garrod’s concepts for many years [37]. Today, we recognize that Garrod’s interpretation was correct, and also that mutations in the *HGO* gene causing a deficiency in hepatic homogentisate 1,2-dioxygenase (EC 1.13.11.5) activity result in an accumulation of HGA and its clinical sequelae such as ochronosis, the yellowish staining of connective tissue by HGA [38]. The major impact of a metabolic biomarker of disease (HGA) is that the mechanism when unmasked can lead to potential therapies of the disease. In the case of alkaptonuria, nitisinone has been shown in several studies to reduce the circulating levels of HGA. Nitisinone is an inhibitor of 4-hydroxyphenylpyruvate dioxygenase (EC 1.13.11.27), the enzyme responsible for the formation of HGA. A daily dose of 2 mg slowed progression of alkaptonuria and arrested ocular and ear ochronosis [39]. This old example of alkaptonuria is a clear-cut prototype for a metabolic biomarker of disease that originates in the liver, which has led to both an understanding of the disease mechanism and its potential treatment. Sadly, many recent examples of liver disease metabolic biomarkers have not lived up to this paradigm.

Alpha-fetoprotein (AFP) was reported in 1956 to be in human fetal serum but not in the serum of healthy adults. The production of AFP by fetal liver largely ceases before birth [40]. The discovery a few years later of AFP in animal models with hepatocellular carcinoma (HCC) [41] led to clinical investigations that associated AFP with HCC. It has been stated that ~70% HCC secrete AFP [42] and up to 40% of HCC patients may not show elevated serum AFP [43]. This suboptimal sensitivity is coupled with specificity issues in relation to premalignant liver diseases such as hepatitis and cirrhosis, together with ovarian and testicular malignancies. Therefore, the clinical interpretation of serum AFP with respect to HCC requires care. Nevertheless, serum AFP is widely used as both a diagnostic and prognostic biomarker for HCC [42,43,44]. In these regards, it is recognized that it should be replaced with more specific and sensitive biomarkers [43,44]. Neither European nor American guidelines for HCC screening include serum AFP concentration [45]. 

Although osteopontin (OPN), a protein normally expressed in kidney and bone, has a high sensitivity for the detection of HCC, its elevation can be linked to more than 30 types of cancer [45] and to many other diseases, including diseases of the liver [46]. Its employment as a HCC risk biomarker is clearly inappropriate. We will examine below whether metabolomics can disclose liver disease biomarkers with high sensitivity and especially with high specificity. 

Of the biomarkers for liver disease discussed above, the determination of metabolite HGA to diagnose the rare inborn error of metabolism alkaptonuria is by far the most sensitive and specific. Studies in experimental animals in the 1950s suggested that homogentisate 1,2-dioxygenase was expressed in liver, to a lesser extent in kidney, and with little enzyme activity reported for heart, skeletal muscle, brain, intestine, spleen, and blood [47]. Contemporary biochemical and molecular methodologies have recently revealed that homogentisate 1,2-dioxygenase is expressed in human and mouse brain, explaining the various observations of brain pigmentation found in cases of alkaptonuria [48]. Both AFP and OPN are compromised by insufficient specificity, which would require them to be used in combination with other biomarkers for liver disease risk.

## 5. Biomarkers of Premalignant Liver Disease

### 5.1. Alcoholic Liver Disease (ALD)

Excessive alcohol consumption is a global healthcare problem that accounts for almost 1% of all global deaths and 50% of all liver cirrhosis-attributable deaths [49]. The spectrum of hepatic lesions includes steatosis, alcoholic steatohepatitis (ASH), alcoholic hepatitis, fibrosis, cirrhosis, and HCC. Alcohol is a principal cause of end-stage liver disease, for which the only curative treatment is transplantation [50]. The insult on the liver by alcohol is closely related to the fact that the liver is the site of most of the metabolism of alcohol. Alcohol dehydrogenase (ADH; EC 1.1.1.1) converts ethanol to acetaldehyde with the generation of NADH reducing equivalents. Subsequent metabolism by acetaldehyde dehydrogenase (ALDH; EC 1.2.1.3) generates further equivalents of NADH. The elevated ratio of NADH/NAD^+^ due to excess alcohol consumption is responsible for many of the biochemical consequences in the liver. For example, lactic acidosis, hyperuricemia, enhanced lipogenesis, and depressed fatty acid β-oxidation have long been known to be driven by excess hepatic NADH [51]. However, the influence of ethanol exposure on lipid metabolism is considerably more complicated than redox inhibition of fatty acid β-oxidation [52]. 

Much of the understanding of the mechanisms of liver disease have been generated using animal models. In pioneering studies, rats fed a 5% ethanol diet (36% total calories) had a plasma glycerolipid profile that mirrored the serum ethanol profile. Relative to paired rats fed a sucrose diet, the ethanol-fed rats displayed a 3-fold increase in total hepatic lipids and an 8-fold greater hepatic triglyceride content [53]. This early work led to the establishment of the Lieber-Decarli experimental alcohol diet [54], which is still widely employed [55]. Binge ethanol administration to mice (5 g/kg in three divided doses over 36 h) has also been used [56], in which case, hepatic *S*-adenosylmethionine (SAM), cysteine, and glutathione were decreased, while hypotaurine and taurine levels were elevated. These findings were interpreted as being due to both oxidative injury and a rapid elevation in cysteine dioxygenase (EC 1.13.11.20) activity, responsible for the production of hypotaurine and taurine. These markers could be attenuated by the co-administration of betaine, thought to be due to the regulation by betaine of hepatic levels of SAM and GSH [56]. Changes in hepatic lipid profiles occurred after chronic feeding of Yucatan micropigs (20–40 kg) with a 40% ethanol folate-deficient diet. In alcoholic pigs, hepatic triglycerides were elevated with increased desaturation of fatty acids (16:0 to 16:1n7 and 18:0 to 18:1n9) by stearoyl-CoA desaturase (SCD; EC 1.14.19.1) and decreased fatty acid elongation pathway (ELOVL5; EC 2.3.1.199) and phosphatidylethanolamine *N*-methyltransferase (PEMT; EC 2.1.1.17) activity. This latter enzyme attenuation led to a shift from phosphatidylethanolamines to phosphatidylcholines in the liver [57].

**Figure 2:**
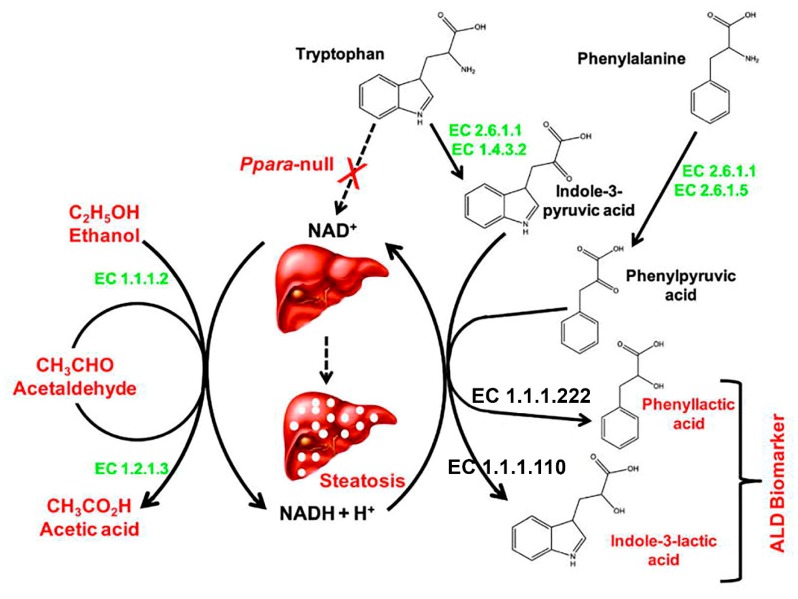
Generation of mechanism-based biomarkers of ALD (adapted from Manna et al., 2011 [59] with permission).

The above studies of the effect of alcohol administration were highly targeted, and therefore, limited in their description of the hepatic metabolic phenotype induced by alcohol. They were also limited by the vastly different protocols of ethanol administration. A study in mice was conducted using the Lieber-Decarli diet treated wild-type (WT) and *Ppara*-null mice (PPARα is a nuclear receptor that regulates much of lipid metabolism including fatty acid β-oxidation) [58]. Six months’ chronic alcohol exposure led to increased hepatic triglyceride accumulation in the *Ppara*-null mice. Urines collected from 2 to 6 months were analyzed using an untargeted metabolomic protocol by UPLC-ESI-QTOFMS, and showed differential elevated metabolite profiles for the WT and null mice. In WT mice, the principal elevated urinary metabolites resulting from alcohol administration were ethyl sulfate and ethyl-β-D-glucuronide, secondary metabolites of ethanol, together with 4-hydroxyphenylacetic acid and its sulfate conjugate. These were also found for the null mice and, in addition, elevated urinary excretion of indole-3-lactic acid was found only in the *Ppara*-null mice, which was mechanistically related to the administration of ethanol in these animals. In a subsequent and more detailed investigation [59] that used WT and *Ppara*-null mice with two different strain backgrounds, indole-3-lactic acid and phenyllactic acid were reported as ALD biomarkers, with their formation arising from their corresponding pyruvic acids having been driven by the NADH hepatic overload due to ethanol consumption (Figure 2). The mechanism-based biomarkers also shed light on the development of steatosis, driven by the deficit in NAD^+^ and the hepatic increase in NADH. The redox inhibition of fatty acid β-oxidation is an initial step of triglyceride and lipid droplet accumulation in the liver [52]. Metabolomic investigations in rats fed the Lieber-DeCarli liquid diet for 2 and 3 months have been conducted using high-field ^1^H and ^31^P NMR. These studies reported a two-fold increase in plasma triglycerides and a halving of plasma free fatty acids, mirroring smaller but statistically significant changes in the liver. Both total and free cholesterol were increased two-fold in the liver [60]. Metabolomics has identified specific lipids in serum that were associated with alcohol-induced liver diseases, specifically, *N*-lauroylglycine identified cirrhosis with 100% sensitivity and 90% specificity, while decatrienoic acid could evaluate liver disease severity with 100% sensitivity and specificity [61]. 

*N*-Acetyltaurine (NAT) has been reported to be a biomarker of alcohol exposure in mice, arising from metabolism of ethanol to acetaldehyde via ADH and CYP2E1 (EC 1.14.13.n7), and further by ALDH to acetate [62]. NAT is not specific to alcohol exposure, since it has been described as a biomarker of gamma-irradiation in both rats [63] and rhesus monkeys [64]. NAT urinary excretion has been reported in healthy human subjects who drank alcohol (0.66 to 0.84 g/kg) [65]. In blood, NAT concentration as a biomarker of alcohol exposure was of limited value [66]. To date, NAT has not been evaluated with respect to liver disease.

Chronic alcohol exposure in both experimental animals and humans leads to functional perturbations in the intestinal microbiota as determined by metabolomic investigations of intestinal metabolites. A wide range of altered intestinal microbiota metabolites has been reported, including decreased amino acids, changes in steroid, lipid, carnitine, and bile acid metabolism. Short-chain fatty acids (SCFAs) that are produced by bacterial fermentation were lowered by alcohol administration to rats, with the exception of acetate, which is an end-product of ethanol metabolism [67,68]. Additionally, saturated long-chain fatty acid (LCFA) biosynthesis by the microbiota is reduced by ethanol administration. These attenuated LCFA metabolites have been shown to contribute to alcohol-associated dysbiosis, influencing ALD [69]. Microbial metabolites combined with reduced levels of *Lactobacillus* trigger intestinal inflammation and liver disease following alcohol administration highlighting the role of gut microbiome-liver cross talk in ALD [49]. Studies that identified metabolomic and lipidomic biomarkers of alcoholic liver disease are listed in Table 1.

### 5.2. Cholestasis

Cholestasis is the impaired formation or secretion of bile into the small intestine, and can be classified as intrahepatic or extrahepatic, together with obstructive or nonobstructive. There are many causes of the various manifestations of cholestasis including gallstones, malignancy, and defective bile acid synthesis and secretion [76]. Metabolomics has been employed to attempt to distinguish between the different mechanisms of cholestasis. In the first such study, rat models of inhibited biliary secretion (intrahepatic) and obstructed bile flow (extrahepatic) were employed, and urine was analyzed by ^1^H NMR. It was concluded that bile acids, valine, and methyl malonate were possible cholestatic biomarkers [77]. These biomarkers did not appear to be specific to cholestatic injury. Another early approach was to use metabolomics to understand the metabolic consequences of perturbed bile acid (BA) homeostasis, as occurs in cholestasis. The farnesoid X receptor (FXR) is a nuclear receptor that regulates genes involved in BA synthesis, metabolism, and transport. *Fxr*-null and WT mice dosed with the FXR ligands CA or LCA generated metabolites indicative of intrahepatic cholestasis. These included the sulfate and β-D-glucuronic acid conjugates of *p*-cresol [78], a fermentation product of tyrosine produced by *Clostridium difficile* in the gut [79], thereby providing further evidence of gut microbiota-liver crosstalk. Other metabolites related to cholestasis included corticosterone and CA metabolites, with the latter being produced by induced CYP3A11 [78]. Furthermore, in LCA-induced experimental intrahepatic cholestasis in mice, TGFβ-SMAD3 signaling mediated the alterations in phospholipid and BA metabolism [80]. In a rat model for cholestasis, mass spectrometry-based targeted metabolomics revealed elevations in urinary taurine and hypotaurine (5- to 9-fold). The largest increases between cholestatic and control rats were for CA, LCA, deoxycholic acid, and ursodeoxycholic acid (10- to 23-fold, respectively) [81]. Four independent rat studies that employed the experimental cholestatic compound α-naphthylisothiocyanate (ANIT) reported that both free and conjugated primary BAs were significantly elevated above controls by ANIT administration [82,83,84,85]. It has been demonstrated that several traditional Chinese medicine (TCM) remedies for treating jaundice can reverse the metabolomic fingerprint of ANIT, and therefore, protect against ANIT-induced cholestasis. These treatments include paeoniflorin (from the dried root of *Paeonia lactiflora*) [83,84], rhubarb [85], Yinchenhao decoction (from the above ground parts of *Artemisia annua*) [86], chicken bile powder (containing mainly taurochenodeoxycholic acid that is deconjugated in the gut producing the primary BA that is a FXR ligand) [87], Huangqi decoction (a TCM comprising Radix Astragali and Radix Glycyrrhizae) [88], gentiopicroside (from *Gentiana rigescens* Franch. ex Hemsl.) [89], and Da-Huang-Xiao-Shi decoction [90]. In addition to TCMs, melatonin (100 mg/kg p.o.) has been administered to rats 24 h after they had received ANIT (25 mg/kg i.p.). This high dose of melatonin (relative to the 4-20 mg/kg doses used in mouse melatonin studies [91,92]) produced a modest reduction in serum liver enzymes and bilirubin with a less severe liver histology. The metabolomic changes in serum due to melatonin administration were unexceptional and, in part, derived metabolically from melatonin [93]. The mechanism of ANIT-induced cholestasis continues to be investigated using metabolomic tools. The plasma and liver biomarkers described in mice administered ANIT gave rise to the conclusion that the cholestatic liver injury might correlate significantly with hepatocyte necrosis, metabolic disorders, and an imbalance of intestinal microbiome ecology as a result of BA accumulation [94]. 

A metabolomic investigation has also been reported, whereby regulation of BA metabolism by the nuclear receptor PPARα and inhibition of NF-κB/STAT3 signaling protected against cholestasis induced by ANIT [95]. Furthermore, a lipidomic study of ANIT-induced intrahepatic cholestasis uncovered the role of the aryl hydrocarbon receptor (AHR) in regulating expression of choline kinase (CHK) in mice. Knockout of the *Ahr* gene significantly reversed ANIT-induced lipid metabolism via *Chka* expression, and reversed the intrahepatic cholestasis [96]. Vascular protein sorting-associated protein 33B (VPS33B) is involved in the trafficking of intracellular proteins to distinct organelles. Mutations in *VPS33B* are associated with a neonatal syndrome that includes cholestasis (OMIM 208085). Using the lipidomic and metabolomic profiles of hepatic *Vps33b*-null male mice, which displayed cholestasis with elevated serum liver enzymes and total bilirubin and total BAs, demonstrated the importance of VPS33B in BA, glycerolipid, phospholipid, and sphingolipid metabolism. In particular, the elevation of hepatic ceramides was thought to influence apoptosis and the progression of cholestasis [97].

Bile duct ligation (BDL) is a nonchemical means to produce experimental cholestasis in rats. Compared with sham operated rats, BDL rats displayed oxidative stress, with diminished serum GSH, total antioxidant capacity, and superoxide dismutase and glutathione peroxidase activities, with upregulated serum malondialdehyde. Changes in certain amino acids, lipids, Krebs cycle intermediates, and lactic acid were signs of the effects of cholestasis on energy metabolism [98]. The BDL cholestasis rat model was shown to generate similar metabolic characteristics as thioacetamide (TAA)-induced cholestasis in rats, with excessive fatty acid oxidation, insufficient glutathione regeneration, and disturbed gut microbiota. These features in both rat models could be reversed by the TCM Huang-Lian-Jie-Du-Decoction [99]. A metabolomic study recently compared three models of chemically-induced cholestasis, using ANIT, 3,5-diethoxycarbonyl-1,4-dihydrocollidine (DDC), or LCA. BAs were increased in all three models, whereas arginine was decreased. Hepatic protoporphyrin IX, a metabolic precursor of heme and cytochrome c, was increased only in the DDC model [100].

**Figure 3:**
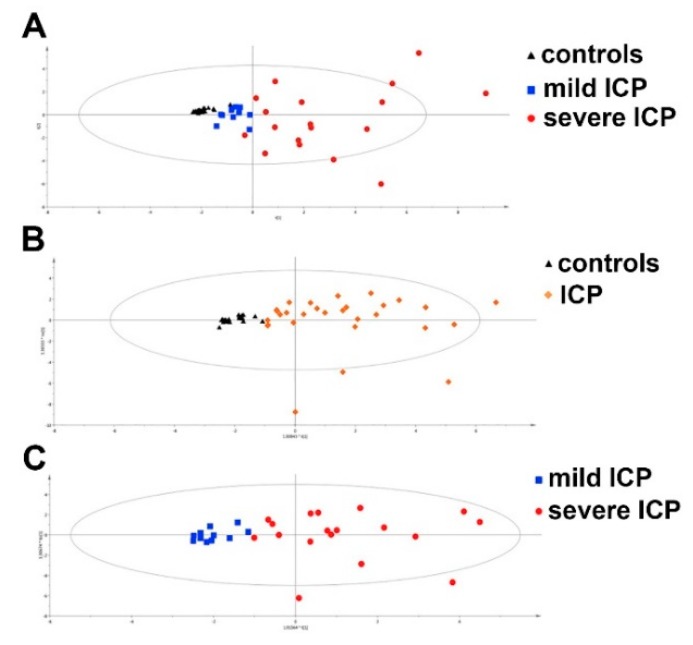
(**A**) PCA scores plot for controls vs. mild ICP vs. severe ICP; (**B**) OPLS-DA scores plot for controls vs. ICP; (**C**) OPLS-DA scores plot for mild ICP vs. severe ICP. Note the data clustering and separation (taken from Li et al., 2018 [112] with permission).

Both primary biliary cholangitis (PBC) (previously known as primary biliary cirrhosis) and primary sclerosing cholangitis (PSC) are chronic cholestatic liver diseases. PBC and PSC patients were investigated using targeted profiling of serum BAs. In PBC with cholestasis, total primary BAs (CA and chenodeoxycholic acid) were 13.5-fold higher than noncholestatic donors, in particular, their taurine conjugates (34- to 46.5-fold accumulation) [101]. A similar pattern of elevated free and conjugated primary BAs was reported in another PBC metabolomic investigation. The total secondary BAs (deoxycholic acid and LCA) were not significantly altered in PBC, nor were the 6α-hydroxylated BAs (hyocholic acid and hyodeoxycholic acid). In PSC with cholestasis, primary BAs were more abundant and both secondary BAs, and 6α-hydroxylated BAs were significantly reduced. The authors recognized that the BA composition of bile requires determination in these two cholestatic diseases [102]. Similar findings were reported in a later study that also included some small changes in free fatty acids and markers of inflammation and oxidative stress [103]. Furthermore, BAs increased during progression of PBC with a decline in acylcarnitines, such as propionyl and butyryl carnitine [104]. The metabolic signatures of PBC and celiac disease have been compared and contrasted with healthy controls using ^1^H NMR-based metabolomics on serum and urine. Both diseases showed distinct metabolite patterns, although relatively few metabolites, such as pyruvate, lactate, glutamate, glutamine, hippurate, and trigonelline (a metabolite of niacin also found in coffee) were described [105]. It is unclear whether the differences described were due to dietary factors. Intrahepatic cholestasis of pregnancy (ICP) has an incidence of between 0.1% (Europe) and 15.6% (South America) [106]. A urinary metabolomic study of ICP revealed several significant predictive biomarkers of ICP, including the primary BA metabolites glycocholic acid and chenodeoxycholic acid 3-sulfate [107]. In a serum targeted metabolomics ICP study, 60 BAs were detected of which most conjugated BAs were elevated in ICP. Metabolomics was also employed to monitor BAs during treatment with ursodeoxycholic acid [108]. Targeted metabolomics of urinary sulfated BAs was used to define biomarkers for the diagnosis and grading of ICP. Total sulfated BAs were remarkably increased in ICP, particularly those formed from glycine and taurine conjugated BAs. Clear clustering and separation of the PCA and OPLS-DA scores for controls, mild ICP, and severe ICP were reported, and are depicted in Figure 3. In order to better understand how ICP endangers the fetus and the links between fetal BA homeostasis and sulfation capacity, a metabolomic investigation in pregnant swine was conducted. It was found that sulfation played a pivotal role in maintaining BA homeostasis in the fetus. Furthermore, fetal mortality showed an exponential increase in relation to the total BA increase from week 60 to week 90 [109]. A controversial condition related to ICP that is asymptomatic and difficult to distinguish from ICP is asymptomatic hypercholanemia of pregnancy (AHP). A targeted metabolomics study was undertaken in order to establish a differential diagnosis of AHP. Compared to a control group, AHP had several higher urinary BAs and sulfated BAs than controls, and more that were lower in AHP than ICP. Glycocholic acid and tauro-ω-muricholic acid were a potential combination biomarker for AHP, whereas a further combination biomarker involving BA sulfates could distinguish AHP from ICP [110]. Metabolomic profiling of maternal hair was conducted to find predictive biomarkers of ICP. Despite the identification of 105 metabolites in hair, none was associated with ICP [111]. 

Cholestasis may also occur in neonates. Infantile hepatitis syndrome (IHS) and biliary atresia (BLA) are the most common in the first three months of life. Using GC-MS metabolomics on urine, it was reported that IHS could be distinguished from BLA with the biomarkers *N*-acetyl-D-mannosamine and α-aminoadipic acid [113]. A summary of studies is given in Table 2. 

### 5.3. Fibrosis and Cirrhosis

Fibrosis occurs when damage to the liver causing overactive wound healing leads to the formation of scarring or deposition of extracellular matrix proteins including collagen. This process occurs in most chronic liver diseases, and can ultimately lead to cirrhosis and liver failure. Such end-stage liver disease may require transplantation [114]. Fibrosis is staged 0 to 4 by liver biopsy using the METAVIR scoring system, F0 = no fibrosis, F1 = portal fibrosis, F2 = periportal fibrosis, F3 = bridging fibrosis, F4 = cirrhosis. Fibrosis is also graded according to the severity of the underlying disease process, activity grades A0 to A3 [115]. Fibrosis and cirrhosis are primarily caused by hepatitis or chronic alcoholism, but can also arise due to nonalcoholic fatty liver disease (NAFLD), including nonalcoholic steatohepatitis (NASH). In compensated cirrhosis, the liver is still able to perform most of its basic functions despite the scarring. Compensated cirrhosis involves Stage 1 (no varices, no ascites) and Stage 2 (varices, no ascites). In decompensated cirrhosis, excessive scarring inhibits basic liver functions and comprises Stage 3 (ascites ± varices) and Stage 4 (bleeding varices ± ascites) [116]. The 1-year survival for compensated and decompensated cirrhosis is 87.3% and 75.0% and 5-year survival is 66.5% and 45.4%, respectively [117]. As the terminal stages of liver fibrosis that can lead to HCC have a high morbidity and mortality with only transplantation as a therapeutic option, there have been extensive studies using metabolomics to define biomarkers for the underlying disease progression. 

Relatively few investigations have sought biomarkers of fibrosis using metabolomics. The greatest both quantity and quality of potential biomarker data has been leveraged using mass spectrometry methodologies. Metabolic pathways associated with hepatic fibrosis, specifically, for carbohydrates, amino acids, and lipids, have been reviewed [118]. In a Japanese study that employed CE-TOFMS and LC-TOFMS, the progression of fibrosis in NAFLD was reported to be associated with increased serum concentrations of several metabolites, among them the sulfates of the three steroids etiocholanolone (a major testosterone metabolite), dehydroepiandrosterone (a precursor of androgens and estrogens) and 16α-hydroxy-dehydroepiandrosterone (a precursor of estriol). The first of these sulfates decreased in relation to fibrosis progression from F0/F1 to F4, while the last steroid sulfate increased during fibrosis progression, especially when expressed as a ratio to either of the other two sulfates [119]. Although these steroid sulfates and their ratios appeared to be biomarkers of fibrosis progression in NAFLD, the key biomarker, 16α-hydroxy-dehydroepiandrosterone sulfate, has also been reported in serum of patients with breast cancer and endometrial cancer [120]. A Brazilian study in chronic hepatitis C collected large amounts of clinical data on 69 fibrotic patients classified with fibrosis by METAVIR that was significant (≥F2; 42), nonsignificant (<F2; 27), also as advanced (≥F3; 28), nonadvanced (<F3; 41), and as cirrhosis (F4; 18) and noncirrhosis (<F4; 51). ^1^H NMR was used to analyze serum, but not to identify metabolites. The PLS-DA 3-D scores plots showed clustering and separation for F0-F1 vs. F2-F4, F0-F2 vs. F3-F4 with partial separation of F0-F3 vs. F4, leading the authors to hypothesize that their metabolomic strategy could distinguish between significant fibrosis, advanced fibrosis, and cirrhosis [121]. Without knowledge of the altered metabolites central to the metabolomic model used, it is not possible to delineate whether the discriminatory signals arise as biomarkers for the disease process or due to confounding factors such as comorbidities or drug treatment, as commented in another similar case (see below) [122]. A ^1^H NMR-based metabolomic study was conducted in rats injected i.p. for 8 weeks with CCl4. Seven metabolites were diminished in urine of treated rats compared with controls, namely, 2-oxoglutarate, citrate, dimethylamine, phenacetylglycine, creatinine, and hippurate. Only taurine urinary excretion was found to be significantly elevated in this rat model of fibrosis [123]. A subsequent report from this group found more metabolomic changes in their CCl4 fibrosis rat model. They proposed that the TCM *Corydalis saxicola* Bunting exhibited antifibrotic effects by regulating ALT, FXR, COX-2, metalloproteinase-1, and angiotensinogen based upon network analysis with their NMR metabolomic data [124], about which we remain skeptical. Shi-Wei-Gan-Ning-Pill (SWGNP) is a multicomponent Tibetan recipe used to treat viral hepatitis, hepatic fibrosis and steatosis, cirrhosis, and HCC. In a study in the CCl4 rat model, SWGNP was also administered at a low, medium, and high dose, equivalent to 3-, 6-, and 12-times the clinical dose, respectively. ^1^H NMR-based metabolomics was conducted on liver extracts and serum. A total of 39 metabolites were identified in rat liver extracts and 28 in serum. Alterations in energy metabolites suggested that the liver responded to CCl4 crisis by metabolic remodeling from mitochondrial respiration to cytosolic aerobic glycolysis, increased fatty acid β-oxidation, glycogenolysis, and metabolism of ketone bodies. The medium and high doses of SWGNP significantly decreased the histological scores in the CCl4 model, together with fibrosis and oxidative stress markers. SWGNP also reversed changes in amino acids and nucleosides caused by CCl4. The authors concluded that SWGNP could alleviate liver fibrosis caused by CCl4 [125]. Another Tibetan folk remedy has been investigated in the CCl4 rat fibrosis model, that of *Herpetospermum caudigerum* Wall. (HCW), the Himalayan Bitter Gourd, a large climbing plant that grows at an altitude of 1500 to 3600 m, whose dry ripe seeds have been used as a hepatoprotectant. In the CCl4 experiments, HCW was administered at doses of 1 and 3 g/kg. HCW produced similar effects on fibrosis markers as SWGNP, with the exception that the lower dose was more effective than the higher dose. The metabolomic effects and proposed mechanisms were very similar for HCW [126] to those of SWGNP [125]. The active principles of neither of these TCMs have been identified, except that HCW was said to comprise mainly lignans, coumarins, triterpenes, saponins, phenols, essential oils, amino acids, and trace elements [126]. The underlying antifibrotic mechanisms of these TCM remedies remain unknown, despite the clues provided by metabolomics.

Earliest serum biomarkers of liver cirrhosis (LC) were derived from chronic hepatitis B patients in China, and comprised the four primary bile salts found by UPLC-QTOFMS [127]. However, elevated glycine and taurine conjugated primary bile acids are not specific to LC (see above). A similar population studied using GC-MS identified several elevated metabolic intermediates in cirrhotic serum, including butanoic and hexanoic acid [128]. These two SCFAs are presumably products of the gut microflora (see above). Amino acid D- and L-enantiomers in serum and urine have been examined using two-dimensional gas chromatography-time-of-flight mass spectrometry (GC X GC-TOFMS) in 25 LC patients and 16 controls in Germany. No L-amino acids were significantly higher in the serum of LC patients, although several were significantly higher in controls. In contrast, D-alanine and D-proline were significantly elevated in LC serum, and D-valine, D-leucine, and D-threonine were only detected in LC serum [129]. It is attractive to consider these D-amino acids as candidate biomarkers for LC. However, only D-serine and D-aspartate are considered human tissue-derived, while the rest most likely arise from microbial sources, either in the diet or from the gut microbiota [130]. This may be further evidence of gut microbiota-liver cross-talk in liver disease. Further evidence of this crosstalk is furnished by a Chinese study that examined stool samples by UPLC-ESI-QTOFMS taken from cirrhotic patients (etiologies either HBV, HCV or alcohol; 17) and healthy controls (24). The two groups clustered and separated in both the PCA and PLS-DA scores plots. Several metabolites that were reduced in cirrhotic feces, chenodeoxycholic acid, 7-ketolithocholic acid, urobilin, and urobilinogen. A number of metabolites were more prominent in cirrhotic feces, including amino acids, and long-chain fatty acids and their carnitine esters. These findings were interpreted as due to changes in biliary function and the gut microbiota in cirrhosis leading to fat malabsorption [131]. Another Chinese study claimed that taurocholate was not merely a biomarker for cirrhosis progression, but also actively promoted this progression. Of the 12 BAs targeted using UPLC-TQMS, taurocholate increased 76-fold between LC (32) and HV (27). This was said to be due to increased synthesis. In addition, the promotion of cirrhosis progression by taurocholate was postulated to be due to stellate cell activation via the TLR4 pathway [132]. 

We have reported a metabolomic and lipidomic investigation of into Swiss HCC patients (20) using UPLC-ESI-QTOFMS and GC-MS, in which LC patients (7) were included together with healthy volunteers (HV; 6) and an acute myelogenous leukemia (AML) control group (22). With one exception, all the HCC patients also had LC. Interestingly, LC and HCC clustered together in both the unsupervised (PCA) and supervised (PLS-DA) scores plots, and clearly segregated from the HV and AML clusters. This suggests that the greatest insult to liver metabolism resulted from LC rather than HCC. No elevated biomarkers specific to LC were described, although several were found for HCC (see below) [133]. The investigation by GC-MS of urine from HCV-positive untreated Egyptian patients with LC (40) and HCC (55), together with HV (45) essentially confirmed the findings of metabolomic similarity between LC and HCC patients. With the exception of AFP, serum biochemistry was similar for the LC and HCC. Several urinary metabolites were elevated above HV for both LC and HCC in a similar fashion, including serine, glycine, threonine, and citrate [134]. Although not stated, the HCC patients almost certainly also had LC, underlining the difficulties of distinguishing between HCC and LC in studies of this kind for an HCV population. In a Chinese study of LC (20), healthy controls (20) and HCC (59) using UPLC-ESI-QTOFMS, three ions corresponding to canavaninosuccinate (CVS) were virtually absent in LC serum relative to the other groups [135]. CVS is a derivative of aspartate formed from ureidohomoserine; aspartate is further converted to creatine [136]. The extinction of CVS in LC serum is an appealing biomarker for LC, except that it is also massively reduced in plasma of chronic kidney disease patients relative to controls, correlating strongly with the glomerular filtration rate [137]. A US study compared patients with high both liver and kidney disease severity (ascites present, GFR ≤ 60; n = 34) with those with low liver and kidney disease (ascites absent, GFR ≥ 60; n = 69) severity. Using UPLC-ESI-TQMS, 34/1028 plasma metabolites were significantly increased in the severe hepatorenal dysfunction group. The greatest change (2.39) was for 4-acetamidobutanoate, the acetylated metabolite of GABA and a product of arginine and proline metabolism (http://www.hmdb.ca/metabolites/HMDB0003681). Pathway enrichment analysis identified glucuronidation and methylation, together with ascorbate and aldarate metabolism, that were linked to hepatorenal dysfunction [138]. Another study in China used both NMR and UPLC-ESI-QTOFMS to analyze serum from LC (42), HCC (43) and HV (18). Several phospholipids and fatty acids together with bilirubin were elevated in LC vs. HV [139], findings similar to those which we had previously reported in Swiss patients [133]. A UK study that employed both NMR and UPLC-ESI-QTOFMS of plasma from 248 subjects examined the differences between surviving and nonsurviving patients with decompensated cirrhosis. NMR profiles of nonsurvivors had increased plasma lactate, tyrosine, methionine and phenylalanine. UPLC-ESI-QTOFMS showed that lysophosphatidylcholines (LPC) and phosphatidylcholines (PC) were downregulated in nonsurvivors. LPC concentrations negatively correlated with the circulating markers of cell death, M30 and M65. Therefore, metabolomic phenotyping (“metabotyping”) was said to accurately predict mortality in decompensated cirrhosis, due to LPC and amino acid metabolism dysregulation that reflected hepatocyte cell death [140]. Using LC-MS, a Chinese group profiled 43 steroids in the urine of HV (21), LC (21), and HCC (28) relative to urinary creatinine. The PCA scores plot showed some overlap between these three groups. Many steroids in LC displayed lower urinary excretion than HV controls, including pregnanediol, corticosterone, androsterone, etiocholanolone, dehydroepiandrosterone, and testosterone. In contrast, LC urinary excretion of 16α-hydroxyestrone was markedly elevated above HV controls. These findings are consistent with what has been described as a “feminization” phenotype in LC [141]. It is worth noting that these investigators treated the urines with sulfatase and β-glucuronidase prior to steroid analysis to determine total (free plus conjugated) steroids; therefore, their results are difficult to compare with those cited above where sulfated steroids were quantitated [120]. Using GC-MS, serum from Chinese HBV-positive (49), LC (52) and HCC patients (39), together with healthy controls (61) was analyzed. All four groups clustered and separated in the OPLS-DA scores plot. Of the top 30 discriminating metabolites, serine, succinate, malate, 5-oxoproline, glutamate, phenylalanine, ornithine, citrate, and tyrosine were all elevated in LC relative to controls. Palmitate was proposed as a biomarker for cirrhosis development in HBV hepatitis, with high sensitivity and specificity in ROC analysis. The purpose of this study however was to examine the progression of hepatitis B to HCC via cirrhosis [142]. Interestingly, a review of metabolomic studies of hepatitis B, HBV-related LC and HBV-related HCC clearly shows the overlap in these three groups in upregulated metabolites [143]. Oxylipins are another group of lipids that have been investigated in HBV-related LC and HCC. UPLC-ESI-TQMS was utilized to quantitate 18 omega-6 fatty acid-derived oxylipins in serum from patients with chronic hepatitis B (34), HBV-related LC (46), HBV-related HCC (38), and healthy controls (50). Compared with healthy controls, LC had statistically significantly elevated 13-HODE, but lower levels of TXB2 [144]. The 13(*S*)-HODE and 13(*R*)-HODE enantiomers are produced from linoleic acid by 15-lipoxygenase and are credited with differential effects on cell growth and apoptosis [145]. Unfortunately, it was not determined which enantiomer was elevated in plasma of LC patients [144]. Apparently, patients with HBV-related LC can be classified under the theory of TCM as having one of two typical patterns, Gan Dan Shi Re (GDSR) or Gan Shen Yin Xu (GSYX). Serum of cases with GDSR (40), GSYX (41), and those with no obvious pattern (called “Latent Pattern” (LP); 30) were investigated using GC-TOFMS metabolomics. Eight metabolites were specific to the GDSR type of HBV cirrhosis, a separate eight were specific to the GSYX type, and a further 10 metabolites were common to both types. The GDSR metabolites were said to be related to abnormalities in linoleic acid metabolism, while the GSYX metabolites were said to arise from abnormalities in glycine, serine, and threonine metabolism. All these 26 metabolites were potential biomarkers for HBV-related cirrhosis [146]. 

As mentioned earlier, BLA is a neonatal cholestatic condition and is the most life-threatening cholestatic disorder in children. In a Chinese study, liver samples from BLA (52) and IHS (16) were profiled for amino acids and biogenic amines using UPLC-ESI-TQMS. Several amino acids had higher hepatic concentrations in IHS than in BLA. However, histamine was twice as abundant in BLA as in IHS liver. In addition, the degree of fibrosis from F1/F2 to F4 correlated with histamine concentration. Histamine therefore presents a potential target for preventing fibrosis in BLA [147]. 

Several investigators have used ^1^H NMR in the search of biomarkers for liver fibrosis and cirrhosis. For example, a Spanish study of LC with minimal hepatic encephalopathy was conducted by ^1^H NMR, resulting in elevated glucose, lactate, methionine, trimethylamine *N*-oxide (TMAO), and glycerol [148], none of which is specific to LC or even liver disease. A further Spanish NMR study compared liver biopsies from cirrhosis and chronic hepatitis due to HCV, HBV, alcohol, and autoimmunity. Elevated in cirrhosis were glutamate and phosphoethanolamine [149]. A UK study used ^1^H NMR metabolite profiling to compare livers removed from patients with either LC associated with ALD (5) or with NASH (14) with healthy donor transplant livers (16). Cirrhotic livers had significantly increased levels of isoleucine, valine, succinate, lactate, and betaine [150]. Another NMR study was conducted on Chinese patients that included those with HCC. The elevated serum metabolites in LC occurred also when the patients had HCC, with the exception of taurine, namely, acetate, pyruvate, glutamine, α-ketoglutarate, glycerol, tyrosine, 1-methylhistidine, and phenylalanine [151]. A French study using ^1^H NMR examined metabolic differences between alcoholic cirrhotic patients with severe and mild chronic liver failure (CLF) that had been stratified by MELD score. Lactate, pyruvate, glucose, amino acids, and creatinine were significantly higher in patients with severe CLF than mild CLF [152]. These findings cannot be considered as biomarkers of severe CLF, as they are not specific. A Chinese study in compensated cirrhosis (30), decompensated cirrhosis (30), and healthy controls (30) using ^1^H NMR on serum samples reported that succinate, pyruvate, and phenylalanine increased with cirrhosis progression [153]. Yet, again, these cannot be considered as biomarkers due to their lack of specificity. An earlier Canadian study had been the first to profile metabolites in compensated and decompensated cirrhosis patients with HCV, together with healthy volunteers, but used ^31^P magnetic resonance spectroscopy performed on the abdomen over the liver. The acquired spectra showed phosphomonoesters (PME), phosphodiesters (PDE), and β-ATP resonances, the last of which was significantly lower in decompensated cirrhosis vs. the other two groups combined, and the PME/PDE ratio was significantly higher in decompensated cirrhosis than controls. This ratio was interpreted based upon published findings as an indicator of a disturbed endoplasmic reticulum membrane in decompensated cirrhosis [154]. Austrian investigators used high-field ^1^H-MRS and ultrahigh-field ^31^P-MRS to examine in vivo the livers of NAFLD patients with little or no fibrosis and NASH patients with advanced fibrosis. The ^1^H-MRS lipid signal was massively increased in NASH livers over NAFLD livers and cross-correlated with histology from liver biopsies. The lipid saturation, polyunsaturation, and monounsaturation indices did not differ between NAFLD and NASH livers. Moreover, ^31^P-MRS measures of the PME (including phosphoethanolamine) and PDE (including glycerophosphocholine) resonances reflected the severity of fibrosis. Changes in energy metabolism, as reflected by ATP flux, were decreased in advanced fibrosis. This noninvasive real-time profiling technique appeared to be of significant value for investigation of hepatic structure and function [155]. An Italian study combined NMR metabolomics of stool samples with 16S rRNA sequencing of gut microbiota in LC patients (46) and healthy age-matched controls (14). Peripheral blood and liver biopsies were also analyzed together with portal blood from seven cirrhotics and caecal biopsies taken during colonoscopy in 17 LC patients and 6 controls. The metagenomics data demonstrated a marked dysbiosis in LC patients. The principally elevated metabolites in LC feces relative to controls were phenylalanine, threonine, butanoate, methanol, cadaverine, and α-glucose. Using the metagenomics data, eight pathways were underrepresented and two overrepresented in LC. The authors concluded that intervention with prebiotics/probiotics/synbiotics, diet, or fecal microbiota transplant could support development of new customized treatments for LC patients [156]. Interestingly, partial reversal of dysbiosis and metabolomic profile was reported after splenectomy in LC patients (12) [157]. A combined metagenomics and metabolomic investigation of LC was conducted in China with HV (47), compensated LC (49) and decompensated LC (46). Urine was analyzed by UPLC-ESI-QTOFMS and PCA scores plots for total metabolites and a subset of 75 differential metabolites both separated HV from LC urines, with compensated and decompensated LC clustering together. Six metabolites were reported to be lower in LC urine than in HV urine, but none greater [158]. Another combined metagenomics and metabolomics investigation was conducted to compare Turkish patients on a Mediterranean diet (HV, 46; compensated LC, 50; decompensated LC, 43) with American patients on a Western diet (HV, 48; compensated LC, 59; decompensated LC, 50). In this study, ^1^H NMR was used for plasma metabolomics, which showed higher lactate concentrations in Turkey vs. USA. There were similar trends between decompensated LC and HV in both Turkey and USA, with reduced lipids and phosphocholines. Correlation networks in cirrhotics showed differences between the beneficial taxa *Blautia* and *Oscillispira* in Turkish compared with American patients [159]. The metabolomic differences described in this unique study were disappointing and would have greatly benefitted from analysis using MS-based methodology. 

Acute-on-chronic liver failure (ACLF) refers to patients with acute deterioration of liver function in compensated or decompensated but stable cirrhosis. Serum from a group of French compensated and decompensated cirrhosis patients (93) was compared with that from ACLF patients (30) using ^1^H NMR metabolomics. The latter group showed higher serum lactate, pyruvate, ketone bodies, glutamine, phenylalanine, tyrosine, and creatinine [160], none of which is a specific biomarker. A UK study examined plasma by ^1^H NMR for stable cirrhotic patients (18), patients with stable cirrhosis during an episode of encephalopathy (18), together with matched controls (17). With the exception of pyruvate, which was significantly higher, glycolysis end-products and gluconeogenesis precursors (pyruvate, alanine, threonine, glycine and aspartate) were significantly lower in cirrhotics with encephalopathy than without and both higher than controls. There was no discernable effect of encephalopathy on branched-chain and aromatic amino acids or on urea cycle intermediates [161]. Yet, again, such NMR-derived metabolites do not show sufficient specificity to be considered as biomarkers. In contrast, a French group compared hepatic encephalopathy (HE) patients (14) with control patients without neurological disease (27) using UPLC-MS analysis of cerebrospinal fluid (CSF) and plasma. A total of 73 metabolites were identified in CSF including amino acids, acylcarnitines, bile acids, and nucleosides. It was further reported that acetylated amino sugars, acetylated amino acids, and metabolites involved in ammonia, amino acid, and energy metabolism were specifically and significantly increased in CSF of HE patients [162]. These findings underscore the superiority of MS-based over NMR-based metabolomics protocols in terms of metabolite identification. Serum analysis by ^1^H NMR was conducted on a Spanish two groups of HCV patients, one without fibrosis (F0; 30) and the other with cirrhosis (F4; 27). Glucose, citrate, and VLDL1 were significantly elevated, and choline, glutamine, acetoacetate, glycoprotein *N*-acetyl groups, cysteine, histidine, and LDL1 were significantly depressed in the serum of cirrhotic HCV patients. The authors believed that these results provided new biomarkers to distinguish no fibrosis from severe fibrosis (cirrhosis) in HCV infection [163]. An investigation of Italian patients with chronic HCV attempted to diagnose the degree of fibrosis using ^1^H NMR on plasma, serum, and urine samples. Remarkably, these investigators did not identify metabolites, but rather, used statistical analysis of their spectra in an attempt to classify and distinguish chronic hepatitis C (little or no fibrosis) from cirrhosis (severe fibrosis) [164]. This study has been severely criticized not only on the basis of the lack of metabolite identification, but also for the statistical methods employed for data analysis [122]. 

Animal models have also been employed. TAA has been administered i.p. to rats to generate experimental fibrosis and cirrhosis. One such study tracked serum and urine by ^1^H NMR metabolomics over 7 weeks of TAA administration. Liver injury included fibrosis and cirrhosis. TAA was found to increase 2-oxoglutarate and decrease succinate in both serum and urine, while urinary excretion of fumarate, oxaloacetate, and citrate was increased, leading investigators to conclude that TAA impaired the TCA cycle [165]. These and other reported amino acid changes are not specific to fibrosis or cirrhosis. The i.p. administration of dimethylnitrosamine (DMN) to rats produces histologically confirmed fibrosis. UPLC-ESI-QTOFMS metabolomics on serum from control and DMN-treated rats, together with serum from rats treated with DMN together with Yin-Chen-Hao-Tang decoction (YCHT), a TCM long used in the treatment of liver diseases including fibrosis. Biochemical parameters including serum liver enzymes and total bilirubin, together with liver histology, in the YCHT treated rats were intermediate between the controls and the DMN-treated animals. Moreover, several serum lipids, including LPC(18:1), LPC(18:2), oleic acid (18:1), linoleic acid (18:2), arachidonic acid (20:4), and docosahexaenoic acid (22:6; DHA) that were altered by DMN treatment (LPCs ↑, fatty acids ↓), remained relatively stable with co-administration of YCHT [166]. Despite these lipidomic findings, the antifibrotic mechanism of YCHT remains unclear. Another TCM that has been evaluated in the DMN rat liver fibrosis model is Huangqi Decoction (HQD). In these experiments, 16 individual bile acids were profiled by LC-MS and demonstrated that bile acids were elevated by DMN treatment and that HQD restored these to normal levels. Additionally, gene expression related to bile acid synthesis and transport was examined, and also altered by DMN treatment, but restored by HQD [167]. 

Carbon tetrachloride (CCl4) is another hepatotoxin that can produce liver fibrosis in rats. Its effects upon the serum metabolome of rats has been reported using UPLC-ESI-QTOFMS. The protocol involved 12 weeks twice weekly s.c. injections of 50% CCl4 in olive oil at a dose of 5 mL/kg. Blood biochemistry and liver histology were consistent with liver fibrosis. Of the many prominent metabolites detected, two, i.e., cervonoyl ethanolamide (8,11,14-eicosatrienoyl ethanolamide) and β-muricholic acid, were defined as biomarker candidates. Pathway analysis proposed that CCl4 induction of liver fibrosis altered glycerophospholipid metabolism, linoleic acid metabolism, α-linoleic acid metabolism, glycine, serine and threonine metabolism, arachidonic acid metabolism, tryptophan metabolism, and aminoacyl-tRNA biosynthesis [168]. This provided a paradigm for chemically-induced liver fibrosis against which other studies could be compared. CCl4 has also been employed to induce decompensated cirrhosis with ascites in rats, using a similar protocol that that described above. In this study, serum and urine were analyzed by Orbitrap UPLC-MS. Aromatic amino acids, alanine, and bile acids were elevated in the CCl4-treated rats, while LPCs, eicosapentaenoic acid, creatine, carnitine, branched-chain amino acids (BCAAs), and arginine were significantly lowered [169]. 

The TCM used to treat liver fibrosis, Jiaqi Ganxian Granule (JGG), was tested against CCl4-induced hepatic fibrosis in rats. As the mechanism was unknown, detailed UPLC-ESI-QTOFMS metabolomics was conducted on rat serum. Fibrosis markers in serum, namely collagen type IV, procollagen III, hyaluronic acid, and laminin were all significantly increased by CCl4, but normalized by JGG intervention, as was liver histology. Lipid markers that were downregulated by CCl4, but normalized by JGG included sphinganine, dihydroceramide, and monostearoylglycerol. Metabolites that were upregulated by CCl4 but normalized by JGG were the bile acid 3,7-dihydroxy-12-oxocholanoic acid, the phosphatidylinositol PI(18:0/16:0), the ethanolamide metabolite of DHA, LPC(22:6), and PC(20:4/18:2). JGG, therefore, affected sphingolipid and glycerophospholipid metabolism among other pathways [170]. These represent further examples of where metabolomics has informed about the mechanism of action of a TCM on liver disease. A similar study reported in Chinese that *Scutellariae* Radix decoction, prepared from the root of a flowering plant of the mint family, and baicalin, a flavone glycoside purified from *Scutellaria baicalensis*, were effective against liver fibrosis in this rat model. UPLC-ESI-QTOFMS analysis showed that several elevated metabolites in fibrotic rat urine were ameliorated by the decoction treatment, including, L-tryptophan, 3-methyldioxyindole, 5-hydroxyindoleacetylglycine, kynurenic acid, 4-(2-amino-3-hydroxyphenyl)-2,4-dioxobutanoic acid, methylmalonic acid, and L-leucine. Baicalin treatment also reversed these urinary metabolites with the exception of L-leucine [171]. Another rat model of fibrosis uses dimethylnitrosamine (DMN) i.p. administration over a period of 8 weeks. Cultured bear bile powder (CBBP) has been used as a TCM to treat liver diseases for thousands of years. Using Orbitrap UPLC-MS, it was reported that CBBP co-administration (65, 130 and 260 mg/kg) restored the lowered serum concentrations of eicosapentaenoic and docosahexaenoic acids that occurred when DMN provoked fibrosis. CBBP had the additional effect of inducing the expression of the nuclear receptors PPARα and PPARγ. Moreover, expression of four PPARα-regulated genes involved in fatty acid β-oxidation (*Cpt1b*, *Cpt2*, *Mcad,* and *Hadha*) was decreased by DMN treatment but restored by CBBP, suggesting that CBBP may improve fatty acid β-oxidation. By inducing PPARγ, CBBP decreased the downstream expression of the inflammatory cytokine IL-6, while also inhibiting activation of hepatic stellate cells, thereby ameliorating fibrogenesis [172]. Further details of the aforementioned studies appear in Table 3.

### 5.4. NAFL and NASH

Nonalcoholic fatty liver disease (NAFLD) is the most common liver disorder in Western countries, affecting 17–46% of adults. NAFLD includes two pathologically-distinct conditions with different prognoses: nonalcoholic fatty liver (NAFL) and nonalcoholic steatohepatitis (NASH). “NAFLD is characterized by excessive hepatic fat accumulation, associated with insulin resistance (IR), and defined by the presence of steatosis in >5% of hepatocytes according to histological analysis“ [173]. The diagnosis of NAFLD requires the exclusion of chronic alcohol consumption as a cause. NASH is characterized by the presence of steatosis, inflammation, and ballooning degeneration of hepatocytes, with or without fibrosis [174]. NASH can progress to cirrhosis in up to 20% of cases [173,174]. The definitive diagnosis of NASH requires a liver biopsy [173]. A number of biomarkers of NAFLD have been evaluated, including fatty liver index (FLI), NAFLD liver fat score (NAFLD-LFS), hepatic steatosis index (HSI), visceral adiposity index (VAI), and triglyceride x glucose (TyG) index. When steatosis was histologically graded as none (<5%), mild (5–33%), moderate (33-66%), and severe (>66%), with the exception of VAI, all biomarkers showed a linear trend across the steatosis grades. The authors concluded, “More research is needed to identify truly independent and quantitative markers of steatosis” [175]. Metabolomics, therefore, has a role to play in delivering biomarkers for steatosis and its progression. Recently, it has been argued that NAFLD patients should be classified into different subtypes dependent upon perturbation of the principal pathways regulating fatty acid homeostasis. Specific serum lipid signatures can be associated with individual mechanisms of progression from steatosis to NASH, and possibly lead to novel and specific NASH therapies [176]. Such metabolomic approaches that help refine definition of the disease phenotype have now been integrated into orthogonal technologies, such as genomics, proteomics, structural biology, imaging [177], and metagenomics.

Investigation of both NAFL and NASH in a metabolomic context is a relatively recent endeavor. Because of the nature of the disease, many investigators have focused on the lipidome. Until a decade ago, the plasma lipidome of NAFLD and whether or not NASH expressed a distinct lipidomic signature were unknown. An early US study examined plasma lipid profiles in both NAFL and NASH compared to healthy controls (HV), and reported significantly increased monounsaturated fatty acids (MUFAs) with an altered pattern of polyunsaturated fatty acids (PUFAs) in both NAFL and NASH. Moreover, the progression of NAFL to NASH was characterized by an increase in the lipoxygenase metabolites 5-HETE, 8-HETE, and 15-HETE. Interestingly, the nonenzymic oxidation product of arachidonic acid, 11-HETE, was significantly increased only in NASH [178]. A Spanish group reported an altered pattern of serum phosphocholines and potentially antioxidant lyso plasmalogens [PC(P-24:0/0:0) and PC(P-22:0/0:0)] in NASH compared to stage 3 hepatic steatosis. Several sphingolipids were also altered in NAFLD compared with healthy subjects. Furthermore, arachidonic acid and glutamate were both decreased in NASH. Metabolic profiling by these authors of an animal model for NAFLD (glycine *N*-methyltransferase *Gnmt*-null mice) produced finding consistent with the patient observations [179]. Serum lyso plasmalogens are therefore potential biomarkers for NASH. Another US study of NAFL, NASH and HV found that NAFLD patients had perturbed glutathione metabolism compared to HV, with markedly higher conjugated primary bile acids in plasma. NASH patients displayed lower long-chain fatty acids, higher carnitine and short-chain acyl carnitines, together with several other metabolites. While the metabolomic fingerprints could distinguish NAFL or NASH from HV, they could not distinguish between NAFL and NASH [180]. A ^1^H NMR-based study in China investigated NAFLD patients and HV, together with mice fed a methionine- and choline-deficient (MCD) diet as a model for NAFLD. Based upon both clinical and animal model findings, four potential biomarkers of NAFLD were proposed: serum glucose, lactate, glutamate/glutamine, and taurine [181]. None of these “usual suspects” provides a basis for evaluating the progression of NAFLD due to lack of specificity. A dietary intervention study in US patients with NAFL examined the effect of insulin sensitivity on the plasma metabolome in NAFL. The pattern of LPCs, in particular LPC(16:0), which was significantly lower in insulin resistant NAFL patients (see Table 4), was put forward to potentially provide biomarkers for NAFL-associated insulin resistance [182]. Serum BA concentrations have also been investigated in NASH and reported to be elevated both fasting and after a fatty breakfast designed to contract the gall bladder. Elevated fasting BAs included secondary BAs, which are formed by dihydroxylation of primary BAs by gut microbiota species belonging to the orders *Bacteroides*, *Clostridium,* and *Escherichia*, which may be increased in the dysbiosis associated with NAFLD. Altered patterns of circulating BAs in NASH may contribute to hepatic damage [183]. The pattern of BCAAs and acyl carnitines has been investigated in liver samples from healthy subjects, NAFL, fatty NASH, and nonfatty NASH. Hepatic valine was decreased in NAFL, and all BCAAs and phenylalanine were elevated in NASH, with and without steatosis. Certain carnitine esters were elevated in NAFLD (see Table 4). The findings were interpreted as due to oxidative stress and inflammation in the liver [184]. None of these findings yielded a suitably specific biomarker of NASH or its progression. A US group examined whether or not arachidonic acid-derived eicosanoids could distinguish between NAFL and NASH, since lipotoxicity is a key component of the progression of NAFL to NASH. Several such lipids were altered between NAFL and NASH, including elevated PGE2, 13,14-dihydro-15-keto-PGD2, 11,12-diHETrE, 14,15-diHETrE and attenuated 15-HETE (Table 4). It was reported that 11,12-diHETrE, 13,14-dihydro-15-keto-PGD2 and eicosatetraenedioic acid (20-COOH AA) were the top candidate biomarkers to distinguish NASH from NAFL with an area under the receiver operating characteristic curve (AUROC) of 1 for 11,12-diHETrE and 1 for the combination of 13,14-dihydro-15-keto-PGD2 and 20-COOH AA [185]. If confirmed in other studies, these findings would have great potential as biomarkers for NASH in NAFLD. Another potential distinction between NAFL and NASH are ketone bodies, such as acetoacetate and 3-hydroxybutyrate, which are produced in the liver from fatty acids. NASH patients were found to have lower serum ketone bodies than NAFL patients, with a lower serum total free fatty acid level (Table 4) [186]. Although these findings contribute to an understanding of NASH pathogenesis, they are not useful for the generation of biomarkers of NASH. Examination of increasing severity of NAFL in obese patients revealed α-ketoglutarate as the principal marker of NAFL (Table 4) [187]. With a specificity of only 62.5%, α-ketoglutarate is unlikely to be a biomarker for NAFL. Patients undergoing bariatric surgery that have a wedge liver biopsy taken routinely during surgery have been investigated with lipidomics and metabolomics. Patients were classified histologically as non-NASH, non-NAFLD, NAFL, and NASH. *PNPLA3* I148M (isoleucine→methionine) variant was also determined that is more common in NASH. Discovery and validation cohorts were also used. A strong negative correlation was reported between the number of TG double bonds and the TG concentrations in NASH relative to non-NASH livers, for both discovery and validation cohorts. A “NASH ClinLipMet score” was developed based upon (i) clinical variables, (ii) *PNPLA3* genotype, (iii) lipidomic data and (iv) metabolomic data. This was highest performing combination biomarker with sensitivity of 85.5% and specificity of 72.1% for NASH (Table 4) [188]. In terms of biomarker discovery, the large amount of data were derived only from liver biopsies, and so there are no indications how parts (iii) and (iv) of the aforementioned NASH ClinLipMet score relate to, and can be determined from, serum or plasma. A small clinical study was conducted in liver samples from control, NAFL, and NASH patients in which lipidomic analyses were conducted in liver biopsies. These authors identified a signature comprising 32 lipids that distinguished NASH with 100% specificity and sensitivity. This signature comprised various phospholipids, sphingolipids, fatty acids, triglycerides, and cholesteryl esters, measured by LC-MS, which we do not believe could represent a viable biomarker for NASH due to its complexity. Furthermore, five fatty acids were identified as accumulating in NASH that were demonstrated to be toxic to HepG2 cells and primary human hepatocytes in culture (see Table 4) [189]. A Chinese urinary metabolomics study compared NAFL patients with normal liver function with NASH patients with abnormal liver function. Many discriminating metabolites were reported (Table 4) [190], although none displayed a large fold-change or was seen as highly specific to NASH. An elegant study was reported containing several large clinical cohorts containing biopsy-proven NASH patients that also had liver fat determined by CT. Serum metabolomics identified the top metabolite associated with liver fat as a mass of 202.1185^+^. Databases contained a large number of hits for this mass and so a GWAS strategy was adopted yielding SNPs for the *AGXT2* gene whose expressed enzyme produces a metabolite, dimethylguanidino valeric acid (DMGV), which matched this mass [191]. In terms of a biomarker for NASH or NAFL, DMGV displayed a wide overlap between control and NASH with approx. 20% higher mean value for NASH. This is not a viable biomarker and perhaps other ions in their “Top-20” [191] should be investigated. Other investigators chose a single biomarker for progression of NAFL to NASH, i.e. pyroglutamate (5-oxoproline), based upon a serum metabolomic study. 5-Oxoproline had a higher AUROC value than adiponectin, TNF-α, or IL-8 [192]. The utility of 5-oxoproline as a biomarker for NASH is doubtful, as it is often found to be elevated in relation to hepatic oxidative stress. We have recently reported a highly statistically significant upregulation of 5-oxoproline in HepG2 cells treated with the experimental anti-HCV drug [193], in the liver of whole-body γ-irradiated mice [194] and in γ-irradiated HepG2 cells [195].

An in vivo MRS technique has been applied to patients with biopsy-proven NAFL or NASH. Both high-field ^1^H and ultra-high-field ^31^P MRS were employed. Many MRS alterations correlated with NASH, mostly with advanced fibrosis, e.g. phosphoethanolamine/total phosphorus (TP) ratio. ATP/TP declined in advanced fibrosis and ATP flux was lower in NASH [155]. While these rapid noninvasive techniques are useful in a research setting, it is still premature to evaluate their diagnostic potential for NASH. A lipidomic study of patients with chronic hepatitis B virus infection (CHB) with and without NAFLD has been conducted in China. Monounsaturated triacylglycerols (TGs) were found more commonly in NASH patients than non-NASH patients [196]. However, there was considerable overlap between these groups, and examination of the raw data does not support the specificity of monounsaturated TGs, with both saturated and diunsaturated TGs associated with NASH. Patients with steatosis are known to have dysregulation of branched-chain and aromatic amino acids. A metabolomic, transcriptomic and metagenomic study of morbidly obese women with and without steatosis has been reported. The plasma metabolite phenylacetic acid produced from phenylalanine by gut microbiota was the most strongly correlated metabolite to steatosis. Mechanistic studies in human hepatocytes and in mice confirmed this association [197]. Using biopsy-proven patients with normal liver (NL), NAFL, and NASH, serum lipidomics was used to define the pattern of TGs in all three groups. Triglycerides were elevated in the order NAFL > NL ≥ NASH. Of the 28 TGs measured, TG(46:0), (48:0), (53:0), (44:1), (48:1), (49:1), (52:1), (53:1), (50:2), (54:5), and (58:2) were always NAFL > NL and NASH < NAFL. Satisfactory AUROC values were obtained for NAFLD vs. NL. Exclusion of patients with glucose > 136 mg/dL improved the sensitivity and specificity for NASH vs. NAFL [198]. As with all studies of this nature, there was considerable overlap between the three different clinical states in serum metabolite profiles. A lipidomic investigation in Greece reported differences between NASH, NAFL, and healthy subjects for several lipid groups and for certain free fatty acids in serum (Table 4). The authors proposed that their bioinformatic methods could distinguish between NASH, NAFL, and healthy status based upon the determination of 36 lipids, 61 glycans, and 23 fatty acids. Moreover, the authors stated that they could differentiate with very high accuracy (up to 90%) using 10–20 total variables between these three conditions. They also reported that they could robustly discriminate between the presence of fibrosis or not using a model containing 10 lipid species [199]. It is unclear to us at this time how such a complex procedure could be adapted to routine clinical diagnosis. 

A large study in Germany measured plasma and urine metabolomic profiles across a wide range of liver fat content (LFC) that had been determined by MRI in 769 selected nondiabetic patients. A wide number of metabolites correlated both positively and negatively with LFC (Table 4). Usual positive associations included BCAAs and aromatic amino acids and their metabolites. A more unusual metabolite correlating with LFC was 7α-hydroxy-3-oxo-4-cholestenoate [200], which is a metabolite in the primary bile acid synthesis pathway. Unfortunately, its utility as a potential biomarker for NAFLD is reduced by its occurrence in sterol 27-hydroxylase deficiency, familial hypercholanemia and Zellweger syndrome. A Mexican study targeted 31 acyl carnitines and 7 amino acids in relation to obesity and NAFLD. No biomarkers of NAFLD per se were reported [201]. A search for plasma biomarkers of visceral adipose tissue and hepatic triglyceride content (HTGC) has been reported. A significant number of plasma phospholipids were associated with HTGC (Table 4). Similar findings have been reported by other groups. The aromatic amino acids tyrosine and tryptophan were also positively associated with HTGC [202]. No useful biomarkers for NAFL emerged from this study. 

As stated earlier, redox changes in the liver can contribute to both steatosis (NAFL) and hyperuricemia (HU). It has been observed that HU often progresses together with NAFLD, and this has stimulated a metabolomic investigation of HU, HU that progressed to HU with NAFLD within one year, HU with NAFLD, and healthy controls. The principal serum changes were upregulated phosphatidic acid and CE(18:0) and downregulated inosine (Table 4) during progression from HU to HU plus NAFLD [203]. Unfortunately, the exact nature of the phosphatidic acid was not given by the authors, although the empirical formula cited corresponded to PA(16:0/16:0), the exact mass given did not, otherwise this could have been a potential biomarker for NAFLD. 

A dual investigation in human and mouse liver was conducted in which GC-MS analysis of both human discovery and validation sets found two hepatic metabolites negatively correlated with nonalcoholic steatosis score, i.e., nicotinic acid and hydroquinone. When HFD was supplemented with nicotinic acid or hydroquinone, nicotinic acid prevented fat accumulation in mouse liver and reduced serum ALT (Table 4). The authors discussed the use of nicotinic acid as a lipid lowering agent and the potential of future such studies in identifying novel therapeutic targets for NAFLD [204]. Another dual human and mouse liver investigation conducted a metabolomic and lipidomic analysis of *Mat1a*-KO and WT mouse liver and serum. MAT1A synthesizes the methylation cofactor *S*-adenosylmethionine. *Mat1a*-KO mice spontaneously develop steatohepatitis. Based upon the *Mat1a*-KO metabolome that is associated with NASH, serum of biopsy proven NAFLD patients was also analyzed and compared with *Mat1a*-KO mouse findings. The metabolomic signature of these mice, comprising high concentrations of triglycerides, diglycerides, fatty acids, ceramides, and oxidized fatty acids, was present in serum of 49% of NAFLD patients, leading to two subtypes of patient, so-called M-subtype and non-M-subtype. Metabolite patterns also distinguished NAFL from NASH. Potential biomarkers might be used to monitor disease progression and identify novel therapeutic targets [205].

A metabolomic study of Chinese NAFLD patients with and without type-2 diabetes mellitus (T2DM) reported elevated bilirubin, various amino acids, and acyl carnitines, together with oleamide [206]. Many of these metabolic changes were confirmatory of published studies. The elevated acyl carnitines reported are consistent with impaired long-chain fatty acid β-oxidation. Interestingly, we had previously reported a three-fold elevation of plasma oleamide in HCV-positive patients versus HCV-negative subjects [207]. Apparently, these authors did not test their patients for HCV, despite the high prevalence of HCV in liver disease patients in China [208].

The metabolomics of NAFLD has also been investigated in children and adolescents. A noninvasive breath test was employed to examine 21 volatile organic compounds (VOCs). Compared with children with a normal liver, children with NAFLD had significantly greater breath concentrations of acetaldehyde, acetone, isoprene, pentane, and trimethylamine. It is highly likely that the gut microbiota plays a role in the generation of both acetaldehyde and trimethylamine. We agree with the authors that breath testing represents a potential for screening with diagnostic biomarkers of pediatric NAFLD [209]. However, many of these VOCs may not be specific to NAFLD because of the 17 VOCs identified in the caecal contents of mice, eight, including acetaldehyde, were reported for mice fed either the MCD diet or normal chow [210]. An Italian study recruited children with biopsy-proven NAFLD (64) and matched healthy controls (64). HPLC was used to measure oxidative stress markers that arose from excessive consumption of GSH [211]. In obese Hispanic-American adolescents, with and without NAFLD, untargeted high resolution mass spectrometry demonstrated changes in lipid and amino acid biochemistry with a particular effect on tyrosine metabolism (see Table 4) [212]. The effect of NAFLD with and without obesity, together with small intestine bacterial overgrowth, on the urinary metabolome was examined in Italian children. Data were reported on multiple perturbed host and gut microbiota pathways (Table 4), and in particular, on elevated urine glucose concentrations in NAFLD [213]. Again, none of the reported changes met criteria for a diagnostic biomarker, in particular, the biochemical distinctiveness of the findings. A further Italian study investigated obese adolescents with and without NAFLD. Plasma metabolomics established increases in branched-chain and aromatic amino acids, together with certain acyl carnitines in NAFLD subjects (Table 4) [214]. Although one of the elevated metabolites in NAFLD (hydroxydecenoylcarnitine) was an unusual finding, the fold difference between the two groups (±NAFLD) was small with large variances and a borderline statistical significance, reducing the opportunity to develop this as a biomarker. Obese adolescents with and without NAFL and with and without metabolic syndrome (MetS) have been studied for their salivary metabolomic changes. Several fatty acids and sugars were reported to differ between these groups (Table 4) [215]. How NAFLD was diagnosed in adolescents, whether by ultrasound or liver enzyme elevations, made a significant difference to the metabolomic findings, especially with lipid profiles, and amino acid and ketone body plasma concentrations [216]. Another NAFLD study in children and adolescents reported changes in certain plasma amino acids and phospholipids (Table 4). These authors generated a model using random forests machine learning with a sensitivity of 73% and specificity of 97% for detecting NAFLD. Random forests was applied to a combination of metabolite and clinical data, such as waist circumference, whole-body insulin sensitivity index (based on an oral glucose tolerance test) and blood triglyceride level [217]. 

Investigations in animal models have been used frequently to understand the mechanisms of NAFLD and to find biomarkers for disease progression. A mechanistic investigation in MCD diet fed mice with NASH, using UPLC-QTOFMS, reported significant decreases in several serum LPCs with marked increases in tauro-β-muricholate, taurocholate and 12-HETE compared with control mice. These results could be explained by the observed up- and down-regulation of several enzyme and transporter genes. The authors concluded that phospholipid and bile acid metabolism is disrupted in NASH, probably due to enhanced inflammatory signaling in the liver [218]. This group conducted a second study with mice fed MCD, in which they reported an increase in serum oleic and linoleic acids and of nonesterified fatty acids that they attributed to enhanced fatty acid release from white adipose tissue in NASH. They demonstrated that this was due to methionine deficiency and not choline deficiency [219]. Another group fed mice a different NASH-inducing diet based upon lard, cholesterol, and cholic acid. Although this was essentially a proteomic investigation, various key metabolites were measured in liver extracts and found to be altered, including predictable lipid changes, but also perturbations in methionine cycle intermediates (Table 4) [220]. Another strategy for the investigation dietary-induced NASH was reported, whereby livers from mice with a disrupted LDL receptor gene (*Ldlr*-null) that had been fed a western diet (WD; 17% energy as protein, 43% as carbohydrate, 41% as fat, and 0.2% as cholesterol; supplemented with olive oil) were examined. *Ldlr*-null mice fed regular chow served as controls. WD livers displayed a histology and gene expression profile consistent with NASH. Experiments were conducted by replacing the olive oil supplementation with DHA (22:6n-3). As Table 4 shows, multiple lipid classes were either up- or downregulated by WD + olive oil in this genetic/dietary mouse model of NASH. DHA dietary supplementation was effective at protecting against the effects of WD in this mouse line [221]. The effect of NAFLD progression on hepatic BA pools and 70 genes involved in BA homeostasis have been examined in human liver samples. Expression of *CYP7B1* mRNA and protein were highly upregulated in NASH, together with clear changes in glycine- and taurine-conjugated BAs away from the classical BA synthesis pathway towards the alternative BA synthetic pathway (Table 4). These findings were interpreted as an attempt by the liver in NASH to minimize hepatotoxicity [222]. Other investigators have used a 16-week high-fat diet (HFD with 60% calories from fat) in WT mice compared with controls on normal chow (12.7% calories from fat). This HFD regimen produced NAFLD, which was then investigated by ^1^H NMR metabolomics in serum, liver and urine. Elevations in serum and liver glucose and lipids were reported, together with a decreased urinary excretion of amino acids (BCAAs, aromatic amino acids), energy metabolites and gut microbiota metabolites [223]. A similar study has been reported in which the mouse sera were analyzed by UPLC-QTOFMS and GC-MS. Glucose was elevated and GSH attenuated after HFD-induced NAFL. Several serum metabolites were altered and related to oxidative stress, inflammation, and mitochondrial dysfunction (Table 4) [224]. Although this was a detailed account of the effects of HFD-NAFLD on the metabolome, the findings do not lend themselves readily as biomarkers of NAFLD for reasons of specificity. A different diet feeding regimen has been used to generate NAFLD in the mouse without obesity. This procedure used a high-fat, high-cholesterol, cholate diet (HFDCC) and both liver and plasma were analyzed by GC-TOFMS and UPLC-QTOFMS. Total cholesterol and CE(16:1), (18:1), (18:2), (18:3), (20:1), (20:3), (20:4), (22:5), (22:6) were elevated in liver, together with cholic acid, DGs, TGs, CERs, SMs, LPCs, the PC/PE ratio, while PEs were downregulated. The nonlipid metabolites xylitol, xanthosine, squalene, and phenylethylamine were elevated in liver tissue of HFDCC-fed mice. Citrate, G-1-P, and saccharic acid were all downregulated in these livers. Subtle differences were reported for plasma of HFDCC-fed mice, including elevated total cholesterol, CE(16:1), (18:1), (18:2), (18:3), (20:1), (20:3), (20:4), (22:5), cholic acid, deoxycholic acid, CERs, SMs, and PEs, while FFAs, glycerol, TGs, and LPEs were all diminished in pathological livers [225]. Xanthosine, the ribonucleoside of xanthine, could be a potential biomarker when evaluated in patients. However, it was elevated in liver and its levels in plasma were not reported. Moreover, xanthosine has been reported to be a urinary biomarker for nephropathy in T2DM patients [226] thereby reducing its specificity. 

A further means of producing features of NASH in the mouse is with 5-diethoxycarbonyl-1,4-dihydrocollidine (DDC). The three mouse strains A/J, C57BL/6J and PWD/PhJ were placed on a diet supplemented with 0.1% DDC or a control diet for 8 weeks. Livers were analyzed for 44 metabolites by targeted MS methods and also subjected to proteomic and RNA-Seq analyses, which showed that many pathways were altered by DDC treatment, in particular, arachidonic acid and *S*-adenosylmethionine metabolism. However, after Bonferroni correction of their findings for multiple comparisons, the following hepatic metabolites were elevated by DDC: putrescine, arginine, citrulline, cAMP, 2-oxoglutarate, asparagine, and glutamate (Table 4). In silico modelling was conducted to understand the effect of DDC on eicosanoid metabolism [227]. Livers from mice fed a HFD were compared with controls in a wide-ranging lipidomics study that analyzed diacylglycerols (DAG), cholesterol esters (CE), phospholipids, plasmalogens, sphingolipids, and eicosanoids. A large number of differences between HFD and controls were observed (Table 4) [228]. Another NASH-generating diet has been employed in mice, that of a high-trans-fat, high-fructose diet (TFD) for 8 weeks (steatosis) and 24 weeks (NASH). These experiments sought to examine flux through the hepatic TCA cycle using ^13^C NMR-based mass isotopomer analysis, which remained normal during steatosis but was two-fold induced in NASH. In parallel to TCA cycle flux induction, ketogenesis was impaired and hepatic diacylglycerols (DGs), ceramides (CERs) and long-chain acyl carnitines accumulated in the liver (Table 4), suggesting inefficient disposal of free fatty acids. The authors concluded that accumulation of “lipotoxic” metabolites could promote inflammation and the metabolic transition to NASH [229]. As serum or plasma was not analyzed, it is not known whether or not any of the accumulated lipids associated with NASH were also present in the circulation and could be evaluated in patients as potential biomarkers for NAFLD progression. 

Correlations between specific gut microbiome species and plasma lipids in mice fed HFD that developed NAFL or NASH. *Bacteroides uniformis* species decreased while *Mucispirillum schaedleri* species increased in mice with NASH. Interestingly, *Bacteroides uniformis* correlated positively with TGs and negatively with FFAs. *Mucispirillum schaedleri* correlated positively with FFAs, LPC(20:3), LPC(20:4), and DG(16:1/18:2). Mechanistically, it was claimed that *Bacteroides uniformis* increased specific TGs and decreased hepatic injury and inflammation in diet-induced mice [230]. Clearly, these observations need to be independently evaluated and then investigated in NAFLD patients before potential biomarkers can be proposed.

The db/db mouse model of leptin receptor deficiency is currently the most widely-used mouse model of type-2 diabetes mellitus (T2DB). Another means of examining the metabolic pathways associated with NAFL is to reverse the steatosis. Caloric restriction (CR) was applied to obese diabetic db/db mice with insulin resistance and steatosis, which were also compared pre- and post-CR to nondiabetic heterozygous db/m mice without insulin resistance and steatosis. Compared to db/m mice, db/db mice had elevated hepatic ketone bodies, lactate, acetate, glutathione, and various glycerolipids, in particular, diglycerides and triglycerides, many of which were reversed by CR (Table 4). The transcriptomic findings were consistent with these observations [231]. In addition to the db/db mouse, a leptin-deficient obese mouse (ob/ob) has also been developed, which is a model for NAFLD. Homozygous ob/ob mice have been compared with nonsteatotic heterozygous ob/+ mice using high resolution magic-angle spinning (HR MAS) ^1^H NMR. ^1^H signals from lipids were highly statistically significantly elevated in ob/ob livers, as expected. Several other molecules involved in betaine (*N*,*N*,*N*-trimethylglycine) metabolism were altered (Table 4) [232]. 

Rats have also been fed a HFD to induce NASH and serum analyzed by UPLC-QTOFMS. Elevated glucose, triglycerides and cholesterol were indicative of insulin resistance. Altered lipid metabolites involved sphingomyelin (SM), phosphatidylcholine (PC), 13-hydroperoxy-9,11-octadecadienoic acid (13-HpODE), and fatty acids (FA) 20:3, 22:3, 20:1 and phytomonic acid (11,12-methyleneoctadecanoic acid) (Table 4) [233]. This last fatty acid is an unusual finding and, if confirmed, could be evaluated in human samples as a potential biomarker for NASH. Another rat study designed to evaluate the effect of turmeric extract on experimental NASH compared HFD-fed with control-fed rats. UPLC-QTOFMS analysis or serum revealed relatively few upregulated metabolites and a much greater number of downregulated lipids, in particular several steroids, including androgen and corticosteroid metabolites (Table 4) [234]. The only highly statistically significant upregulated metabolite was the fatty acid FA(28:8), which has been described as a marine ω-3 fatty acid [235] derived from dinoflagellate species [236]. If confirmed and a mechanism for its formation in human liver by fatty acid elongases and desaturases can be described, this would represent a potential NASH biomarker. A study was conducted comparing metabolomic profiles of rat and human liver, and, of particular interest, MCD diet-fed rat liver (model for NASH) and liver from NASH patients. Despite the large number of metabolic differences reported between treated and control rat liver and NASH liver and healthy patient liver, very few metabolites corresponded between MCD rat liver and human NASH liver. In fact, in the scores plot presented, healthy rat liver was closer to diseased rat liver than to healthy human liver, which was itself closer to diseased human liver. Asparagine, citrulline, and lysine, together with stearoyl carnitine, were the only metabolites upregulated in both rat MCD liver and human NASH liver (Table 4) [237]. Interestingly, stearoyl carnitine together with (9*E*)-octadecenoyl carnitine, docosapentaenoic acid and vitamin D2 were elevated in serum of rats fed either HFD (NAFL), MCD diet (NASH), or HFD plus streptozocin (NASH plus T2DM) [238]. These rat observations reduce the potential value of long-chain fatty acyl carnitines, like stearoyl carnitine, as potential biomarkers for clinical NASH or NAFLD progression. Another investigation was conducted in rats focusing on fatty acid profiles in blood cells and the liver of rats fed either a control diet or a HFD/cholesterol diet. Correlations between certain MUFAs and PUFAs were reported for both diets [239]. None of these fatty acids changes were specific enough to be evaluated as biomarkers of NAFLD in patients. Finally, an investigation of the pattern of BAs in serum, liver, and caecal contents was undertaken in rats fed HFD and control diet. Metagenomic analyses established that hyodeoxycholate, which was decreased in both serum and caecal contents of rats fed HFD, was related to the level of the *Bacteroidetes* phylum. The concentration of cholate that was increased in the caecal contents of rats fed HFD, was correlated with levels of *Firmicutes* and *Verrucomicrobia* phyla, but correlated inversely with *Bacteroidetes* [240]. As the BA pattern appeared to be dependent upon the status of the gut microbiota, the data obtained were not useful for evaluation as biomarkers of NAFLD.

#### Overall Summary

A summary of metabolic, metabolomic, and lipidomic investigations into ALD is given in Table 1. It is clear that the hepatic metabolic phenotypes and therefore biomarkers of both chronic alcohol consumption and ALD are far from being defined. Despite the considerable number of published investigations, large variations in study design, species investigated, experimental methodologies and metabolic findings render a consensus opinion difficult to formulate. Nevertheless, it is becoming clear that various lipid classes may play a role in both ALD etiology and in shaping the resultant hepatic metabolic phenotype. Moreover, the recent attention to gut microbiota-liver cross talk offers new avenues to solving the mechanisms of ALD and providing effective predictive biomarkers. The effects of alcohol on lipid metabolism has recently been reviewed [52], as has the Lieber-DeCarli diet as a model for experimental liver disease [55]. 

Regarding cholestasis, it is well known to be associated with elevated hepatic and serum bile acids, and can occur in the diseases PBC and PSC, as well as in pregnancy and in the neonatal period. Metabolomics has revealed that not just the expected primary BAs are elevated in these conditions, but also BA removal is enhanced by sulfation, with various sulfate conjugates found in the urine. More mechanistic investigations were generally conducted in rats, predominantly by administration of the hepatotoxin ANIT. This protocol has found particular utility in the screening of TCMs that have been used for centuries to treat jaundice in China. Attesting to the efficacy of these treatments, the metabolomic signature of ANIT-induced cholestasis was attenuated in all cases. In addition, a number of combination biomarkers have been evaluated for the various manifestations of both clinical and experimental cholestasis, but it remains to be seen if any of these are adopted into routine clinical practice (Table 2).

In the case of fibrosis and cirrhosis, a total of 38 studies are summarized in Table 3, i.e., 22 conducted by MS and 16 by NMR. Nine investigations were conducted in rats and 29 in patients and volunteers. Considering first the NMR-based studies, it should be noted that these investigations in general identify in liver samples, serum, plasma, or urine relatively high concentration metabolites, such as Krebs cycle intermediates, amino acids and simple sugars that have been described as “the usual suspects” [244]. This simple fact renders these targets unsuitable as biomarker candidates for the detection or progression of fibrosis because of their ubiquitous nature. Although ^1^H NMR-based metabolomic studies are seen as having many advantages, such as simplicity, rapidity, and reproducibility, they suffer from modest resolution and sensitivity. MS-based methodologies, in contrast, are able to resolve, identify and quantitate hundreds of molecules in a sample, rather than tens of metabolites by NMR. They have the distinct advantage in the realm of the relatively low concentration constituents of the metabolome. As Table 3 demonstrates, fibrosis progression in NAFLD could be evaluated in terms of decreasing serum concentrations of etiocholanolone sulfate (E) and dehydroepiandrosterone sulfate (D), with concomitant increasing concentration of 16α-hydroxy-dehydroepiandrosterone sulfate (16). Discovery of these molecules as potential biomarkers was assessed in a validation cohort [119]. The ratios 16/D and 16/E exhibited clear statistically significant trends across F0-1, F2, F3, to F4, with sensitivities/specificities of 81%/80% and 76%/85%, respectively [119]. More commonly, MS-based methods have shown elevations in specific serum bile acids in human liver cirrhosis [128,132,135], together with fluctuations in a broad range of urinary steroids [141]. Another common finding were alterations in serum phospholipids in both human liver cirrhosis [135,139,140] and in animal models [169,170,172]. In both the human and animal model investigations, perturbations in metabolic intermediates, akin to those revealed by NMR, have also been described. We consider that only metabolites that appear unique to fibrosis and/or cirrhosis, such as etiocholanolone sulfate, dehydroepiandrosterone sulfate, and 16α-hydroxy-dehydroepiandrosterone sulfate should be evaluated as biomarkers. The unusual metabolite cervonoyl ethanolamide (8,11,14-eicosatrienoyl ethanolamide) was elevated in the rat CCl4 fibrosis model, but has so far not been evaluated in patients with liver fibrosis. However, this fatty acid amide was also elevated in hyperlipidemic rats [245], reducing its potential as a biomarker for fibrosis. 

A total of 30 studies involving adult patients, nine involving adolescents or children, and 21 studies that involve mouse or rat investigations of NAFLD, are included in Table 4. Of these, a total of 49 investigations used mass spectrometry-based methodologies and ten used NMR-based methods. A large quantity of literature has described a wealth of metabolomic and lipidomic investigations into steatosis (NAFL) and NASH, together with experiments in laboratory rodent models. In the most part, this accumulated information largely describes potential mechanisms by which the liver accumulates lipid droplets and the transition to an accompanied inflammation that defines NASH. As Table 4 shows, there is a wealth of information regarding up- and down-regulated molecules in plasma/serum, urine and liver itself. The question is: how useful are these data for the generation of biomarkers of NAFL or NASH, or the progression of NAFL to NASH? The metabolic profiles of liver that have been determined in certain investigations are not immediately useful for biomarker evaluation unless serum/plasma or urine was also investigated. The purpose of a biomarker for liver disease is to avoid liver biopsy. The increase in peripheral fatty acids and acyl carnitines are consistent with the known etiology of fatty liver disease. Elevated concentrations of BAs, BCAAs, and aromatic amino acids are also well-known characteristics of these diseases. The issue is specificity, especially as many of the studies involved obese patients and those with diabetes and insulin resistance, all of which factors could confound the NAFLD findings. In a study of nondiabetic patients with steatosis and NASH, an interesting candidate biomarker emerged, γ-glutamyltyrosine, but unfortunately the change between control subjects and NAFLD patients was small (1.2–1.3-fold, but highly statistically significant) with a number of outliers [180]. Larger fold-changes were observed for acyl carnitines. Lauroyl carnitine was four-fold increased over controls in steatosis and NASH and hexanoyl carnitine was 3.5-fold elevated in NASH but 2.5-fold decreased in steatosis [184]. As no other study reported these acyl carnitine changes in NAFLD, they would need to be independently verified. Nevertheless, acyl carnitine patterns represent potential biomarkers for progression from steatosis to NASH. We have already discussed above 7α-hydroxy-3-oxo-4-cholestenoate [200] as a potential biomarker for steatosis. Providing that the patients under investigation were negative for sterol 27-hydroxylase deficiency, familial hypercholanemia and Zellweger syndrome, with which this BA intermediate is also associated, it could be further evaluated as a potential biomarker for steatosis. Regarding NAFL in children and adolescents, almost all patient groups in Table 4 were also obese. These studies did not appear to yield potential biomarkers of pediatric NAFLD.

NAFLD does not have a natural history in almost all laboratory rodent studies; rather, it is induced with specialized diets or occurs in genetically modified mice, such as leptin-deficient obese mice (ob/ob) or leptin receptor-deficient mice (db/db) (Table 4). Unusual metabolites such as the globotrioseacylceramide Gb3(d18:1/22:1), which was highly statistically significantly elevated in the livers of mice fed a high-fat high-cholesterol diet [228], could not be further evaluated because their serum concentrations were not determined. Another unusual metabolite, phytomonic acid (11,12-methyleneoctadecanoic acid), reported in serum of HFD-induced NASH in rats [233] may also not be useful as human biomarkers of NAFLD, because it may be produced by the gut microbiota, given that its older name is lactobacillic acid. The C8 and C16 acyl carnitines were elevated in liver tissue of mice fed a high-fructose high-trans-fat diet [229] similar to human findings referred to above [184]. However, again there were no serum/plasma data from which to evaluate the potential of acyl carnitines as biomarkers of human NAFLD. Other data from rats with different NAFLD phenotypes pointed to the elevation of stearoyl and elaidoyl [(9*E*)-octadecenoyl] carnitine in serum, with palmitoyl and stearoyl carnitine upregulated in liver tissue [238]. In summary, an abundance of metabolomic data from human and animal model studies of NAFLD provide a number of leads for evaluation of biomarkers in independent trials. 

## Figures and Tables

**Table 1:** Metabolomic and lipidomic biomarkers of alcoholic liver disease.

Species	Alcohol Dose	Pathology	Metabolites Reported	Ref.
Rat	20% or 36% of total calories; 24 days	Hepatomegaly Fatty infiltration	Plasma triglycerides↑ Plasma phospholipids↑ Hepatic triglycerides↑ 8-fold	[53]
Rat	5% alcohol Lieber-DeCarli diet; 2-3 months	Fatty infiltration Mild inflammatory infiltrate; 3 months Mild oxidative stress, 3 months	Liver triglycerides↑ Liver cholesterol↑ Liver phospholipids and lysophospholipids↓	[60]
Rat	6 g/kg alcohol + high-fat diet	Regional laminar necrosis and edema around central vein. Inflammatory cell infiltrate.	Total of 37 core ALD biomarkers identified. Pathways perturbed included TCA cycle, carbohydrate and amino acid metabolism.	[70]
Mouse	5 g/kg every 12 h X 3	Serum ALT↑ Hepatic CYP2E1↑	Malondialdehyde↑ Methionine↑ Hypotaurine↑ Taurine↑ SAM↓ GSH↓	[56]
Mouse 129 Sv WT and *Ppara*-null	4% alcohol Lieber-DeCarli diet; 2–6 months	Little change after 1 month	Ethylsulfate↑ Ethyl-β-D-glucuronide↑ 4-hydroxyphenylacetic acid (4HPAA)↑ 4HPAA sulfate↑ in both WT and null. Indole-3-lactic acid↑ in null only.	[58]
Mouse 129 Sv and C57BL/6 WT and *Ppara*-null	4% alcohol Lieber-DeCarli diet; 1 month	Steatosis in B6 null mice	Indole-3-lactic acid↑ and phenyllactic acid↑ in alcohol-treated *Ppara*-null mouse, both 129 Sv and C57BL/6	[59]
Mouse WT and *Cyp2e1*-null	2.2%, 4.5%, 5.4% Lieber-DeCarli semi-solid diet; 21 days	CYP2E1↑ in WT Microvesicular and macrovesicular steatosis around central vein; WT>null	Hepatic and serum triglycerides↑ in WT only. Urinary *N*-acetyltaurine, 4HPAA sulfate, ethylsulfate, ethyl-β-D-glucuronide↑	[62]
Mouse	4.896 g/kg; 7 days	ALT↑ AST↑ Focal hepatic necrosis Inflammatory infiltrate	Serum Malondialdehyde↑ GSH↓ GSSG↑ Methylglyoxal↑	[71]
Mouse	5% alcohol Lieber-DeCarli diet; 8 weeks	Mild steatohepatitis No fibrosis	Correlation between urinary and fecal metabolites. Many fecal and urinary metabolites altered. Amino acid metabolism perturbed. Indole-3-lactic acid↑	[72]
Mouse *Cramp*-null and WT	5% alcohol Lieber-DeCarli diet; 24 days	Not clearly stated	In alcohol-fed WT, fecal taurine, α-aminoisobutyric acid, nicotinic acid, serine, SCFAs↓ In alcohol-fed null mice, only nicotinic acid↑	[73]
Micropig	40% total calories Folate-depleted diet	Not determined	Hepatic triglycerides↑ SCD pathway↑ ELOVL5 pathway↑ PEMT pathway↓ Phospholipid export↓	[57]
Human	100–300 g/day; 10 days 118 g/day; 11 days, 141 g/day; 8 days	Fatty infiltration	Plasma triglycerides↑	[53]
Human	30 ALD patients (mean daily alcohol consumption 109.7 g/day) vs. 10 healthy controls	Cirrhosis (80%) Decompensated cirrhosis (DC; 23%)	*N*-Lauroylglycine↑ in cirrhosis. Decatrienoic acid associated with disease severity.	[61]
Human	30 Alcohol use disorder (AUD), 13 alcoholic hepatitis (AH) and 16 nonalcoholic controls	ALT↑ (AUD = AH) AST↑ (AH>>AUD)	Seven serum oxylipins and nine fecal oxylipins↑ Results related to inflammation and platelet aggregation. Inflammatory ω-6 PUFA oxylipins counteracted by ω-3 bioactive lipid mediators.	[74]
Human	64 AH patients, 26 DC patients without AH	AST and GGT↑ (AH > DC). Other serum markers and MELD score AH = DC	Metabolomic signature of AH claimed but not disclosed.	[75]

**Table 2:** Metabolomic and lipidomic biomarkers of cholestasis.

Species	Manipulation/Condition	Pathology	Analytical Methodology	Metabolites Reported	Ref.
Rat	Inhibition of bile secretion vs. bile flow obstruction	Intrahepatic cholestasis vs. extrahepatic cholestasis	^1^H NMR	Bile acids↑ Bilirubin↑ vs. Bile acids↑ BCAAs↑ SCFAs↑	[77]
Mouse	*Fxr*-null vs. WT treated with FXR ligands CA and LCA	Cholestasis	UPLC-ESI-QTOFMS	*p*-Cresol sulfate and β-D-glucuronide↑ Corticosterone metabolites↑ Cholic acid metabolites↑	[78]
Rat	Eisai hyperbilirubinemic rat	Cholestasis	UPLC-TQMS	Taurine↑ Hypotaurine↑ Unconjugated primary and secondary bile acids↑	[81]
Rat	ANIT Methapyrilene Dimethylnitrosamine	Cholestasis	UPLC-TQMS GC-MS UPLC-QTOFMS	Bile acids↑ Arginine↓ Pantothenate↑ Protoporphyrin IX↑ Palmitoyl carnitine↑ Arachidonic, linoleic and oleic acids↓	[82,83,84,85]
Mouse	*Vps33b*-depleted mouse	Cholestasis	UPLC-MS	Serum bile acids↑ triglycerides↑ and sphingomyelins↑	[97]
Rat	Bile duct ligation (BDL)	Cholestasis	UPLC-QTOFMS	Phenylalanine↑ Glutamate↑ Tyrosine↑ Kynurenine↑ Lactate↑ LPC(14:0) ↑ Glycine↑ Succinate↑ MDA↑ GSH↑ Valine↓ Isoleucine↓ Citrate↓ Palmitate↓ Taurine↓ LPC(19:0)↓	[98]
Rat	TAA or BDL	Cholestasis	^1^H NMR	BDL vs. TAA: 2-Hydroxybutyrate↑ BCAAs↑ Lysine↑ Arginine↑ Glycine↑ Citrate↑ 2-Oxoglutarate↑ Fumarate↑ Hippurate↑ Phenacetylglycine↑	[99]
Mouse	ANIT or DDC or LCA	Cholestasis	UPLC-QTOFMS	Phospholipids↑ Protoporphyrin IX↑ GSH↓	[100]
Human	Primary biliary cholangitis	Cholestasis	UPLC-QTOFMS	Primary bile acids↑ Phospholipids↑ Oleic and Linoleic acids↑	[101,102,103]
Human	Intrahepatic cholestasis of pregnancy (ICP)	Cholestasis	HPLC-QTOFMS	MG(22:5) ↑ LPE(22:5) ↑ L-Homocysteine sulfonic acid↑ Glycocholic acid↑ Chenodeoxycholic acid 3-sulfate↑	[107]
Human	Hypercholanemia of pregnancy (HCP) vs. ICP	Cholestasis	UPLC-QTOFMS	Sulfated bile acid pattern used for differential diagnosis of HCP and ICP	[110]
Human	Infantile hepatitis syndrome (IHS) vs. biliary atresia	Cholestasis	GC-MS	*N*-Acetyl-D-mannosamine and α-Aminoadipic acid used for differential diagnosis	[113]

**Table 3:** Metabolomic and lipidomic biomarkers of liver fibrosis and cirrhosis.

Species	Manipulation/Condition	Pathology	Analytical Methodology	Metabolites Reported	Ref.
Human	NAFLD	Fibrosis progression	CE-TOFMS LC-TOFMS	F0/F1→F4 Etiocholanolone sulfate↓ Dehydroepiandrosterone sulfate↓ 16α-hydroxy-dehydroepiandrosterone sulfate**↑** (**all in serum**)	[119]
Human	Fibrosis or Cirrhosis	Significant fibrosis, advanced fibrosis, cirrhosis	^1^H NMR	No metabolites reported, only multivariate model used to distinguish pathologies.	[121]
Human	Chronic hepatitis B	Cirrhosis	UPLC-QTOFMS	Glycocholic acid**↑** Glycochenodeoxycholic acid**↑** Taurocholic acid**↑** Taurochenodeoxycholic acid**↑** (**all in serum**)	[127]
Human	Chronic hepatitis B	Cirrhosis	GC-MS	Acetate**↑** Hexanoate**↑** Butanoate**↑** Glucose↓ Sorbitol↓ (**all in serum**)	[128]
Human	Causes not stated	Cirrhosis	2D-GC-TOFMS	D-Alanine**↑** D-Proline**↑** D-Valine**↑** D-Leucine**↑** D-Threonine**↑** (**all in serum**)	[129]
Human	HBV, HCV, alcohol	Cirrhosis	UPLC-QTOFMS	Chenodeoxycholic acid↓ 7-Ketolithocholic acid↓ Urobilin↓ Urobilinogen↓ LPC(16:0)**↑** LPC(18:0)**↑** LPC(18:1)**↑** LPC(18:2)**↑** (**all in feces**)	[131]
Human	HBV, alcohol, PBC, cryptogenic cirrhosis	Cirrhosis	UPLC-TQMS	Taurocholic acid**↑** Taurochenodeoxycholic acid**↑** Tauroursodeoxycholic acid**↑** Glycocholic acid**↑** Ursodeoxycholic acid**↑** Chenodeoxycholic acid**↑** Cholic acid**↑** Taurolithocholic acid**↑** Taurodeoxycholic acid**↑** Hyodeoxycholic acid**↑** Lithocholic acid**↑** Deoxycholic acid**↑** (**all in serum**)	[132]
Human	Chronic hepatitis C	Cirrhosis	GC-MS	Proline**↑** Serine**↑** Glycine**↑** Threonine**↑** Citrate**↑** Xylitol↓ Arabinose↓ Urea↓ (**all in urine**)	[134]
Human	Chronic hepatitis B	Cirrhosis	UPLC-QTOFMS	Phenylalanine**↑** Glycochenodeoxycholic acid**↑** Oleamide**↑** LPC(16:0)↓ PC(16:0/18:2)↓ PC(16:0/22:6)↓ PC(16:0/20:4)↓ PC(18:0/18:2)↓ Canavaninosuccinate↓ (**all in serum**)	[135]
Human	Hepatorenal syndrome	Cirrhosis	UPLC-TQMS	4-Acetamidobutanoate**↑** (**in plasma**)	[138]
Human	Chronic hepatitis B	Cirrhosis	^1^H NMR UPLC-QTOFMS	Tyrosine**↑** Oxaloacetate**↑** Phenylalanine**↑** C16-Sphinganine**↑** Phytosphingosine**↑** Isobutyrate**↑** LPC(18:1) **↑** Linoelaidic acid**↑** Bilirubin**↑** PC(18:4/20:1)↓ PC(14:1/14:1)↓ LPC(16:0)↓ Formate↓ Ascorbate↓ Carnitine↓ α-CEHC↓ (**all in serum**)	[139]
Human	Causes not stated	Decompensated cirrhosis (90-day mortality vs. survivors)	^1^H NMR UPLC-QTOFMS	Isoleucine**↑** Leucine**↑** Lactate**↑** Creatinine**↑** Urea**↑** Tyrosine**↑** Histidine**↑** Phenylalanine**↑** Formate**↑** LPC(16:0) **↑** Pyruvate↓ Choline↓ Phosphocholine↓ Glycine↓ Glucose↓ PC(34:2)↓ PC(18:2/18:2)↓ PC(16:0/18:2)↓ PC(18:0/18:2)↓ LPC(18:2)↓ PC(18:2/18:5)↓ PC(22:5/20:4)↓ PI(37:2)↓ PS(41:4)↓ (**all in plasma**)	[140]
Human	Causes not stated	Cirrhosis	UPLC-Orbitrap MS	16α-Hydroxyestrone**↑** 4-Androstenedione↓ 17α-Hydroxyprogesterone↓ 18-Hydroxycorticosterone↓ Cortisol↓ Cortexolone↓ Allotetrahydrocortisol↓ Deoxycorticosterone↓ Epitestosterone↓ Testosterone↓ Dehydroepiandrosterone↓ Etiocholanolone↓ Tetrahydrodeoxycortisol↓ Androsterone↓ 17α-Hydroxypregnenolone↓ Epiandrosterone↓ 11-Oxoetiocholanolone↓ 7β-Hydroxy-dehydroepiandrosterone↓ Androstenetriol↓ Androstenediol↓ Pregnanediol↓ (**all in urine**)	[141]
Human	Chronic hepatitis B	Cirrhosis (vs. HBV)	GC-TOFMS	Serine**↑** 5-Oxoproline**↑** Phenylalanine**↑** Tyrosine**↑** Ornithine**↑** Citrate**↑** Palmitic acid**↑** Fructose↓ Glutamate↓ Indole-3-acetic acid↓ arachidonic acid↓ 2-Deoxy-D-glucose↓ (**all in serum**)	[142]
Human	Chronic hepatitis B	Cirrhosis (vs. HBV/HV)	UPLC-TQMS	9,10-DiHOME**↑** 13-HODE**↑** TXB2↓ (**all in serum**)	[144]
Human	Chronic hepatitis B	Cirrhosis (GDSR and GSYX patterns, vs. latent pattern (LP))	GC-TOFMS	GDSR vs. LP: Nonanoate**↑** Urea↓ Serine↓ 2-Hydroxybutyrate↓ 2-Hydroxyglutarate↓ Phenylalanine↓ Asparagine↓ Citrulline↓ Tyrosine↓ Arabinose↓ Sorbose↓ Fructose↓ Myristate↓ Palmitolate↓ Palmitate↓ Linolate↓ Tryptamine↓ Glycolate↓ Quinate↓ Petroselinate↓ GSYX vs. LP: 1,5-Anhydrosorbitol**↑** Fructose**↑** 2-Hydroxybutyrate↓ Serine↓ Threonine↓ 5-Oxoglutarate↓ 2-Hydroxyglutarate↓ Phenylalanine↓ Asparagine↓ Tyrosine↓ Arabinose↓ Arabitol↓ Nonanoate↓ Glycerate↓ Pipecolate↓ Glutarate↓ Quinate↓ α-Tocopherol↓ (**all in serum**)	[146]
Human	Biliary atresia (BA) and neonatal hepatitis syndrome (NHS)	Fibrosis F1 to F4	UPLC-TQMS	BA/NHS: Histamine**↑** Methionine↓ Phenylalanine↓ Serine↓ Threonine↓ Valine↓ Glutamine↓ Sarcosine↓ Lysine↓ F4>F3>F1/F2 in BA: Histamine**↑** (**all in liver homogenates**)	[147]
Human	Alcohol	Cirrhosis ± minimal hepatic encephalopathy (MHE)	^1^H NMR	MHE+/MHE-: Lactate**↑** Glucose**↑** TMAO**↑** Glycerol**↑** LDL↓ VLDL↓ Isoleucine↓ Leucine↓ Valine↓ Alanine↓ Acetoacetate↓ Choline↓ Glycine↓ (**all in serum**)	[148]
Human	Chronic hepatitis C, Chronic hepatitis B, Alcohol, Autoimmunity	Cirrhosis	MAS ^1^H NMR	Phosphoethanolamine**↑** Phosphocholine**↑** Glutamate**↑** Aspartate↓ α-Glucose↓ β-Glucose↓ (**all in liver**)	[149]
Human	ALD, NASH	Cirrhosis	^1^H NMR	ALD Cirrhosis: Isoleucine**↑** Valine**↑** 1,2-Propanediol**↑** Succinate**↑** Aspartate**↑** Betaine**↑** Lactate**↑** Glucose**↑** Uracil**↑** Phenylalanine**↑** NASH Cirrhosis: Leucine**↑** Isoleucine**↑** Valine**↑** 1,2-Propanediol**↑** Succinate**↑** Aspartate**↑** Betaine**↑** Lactate**↑** Phenylalanine**↑** Uracil**↑** Uridine↓ Inosine↓ (**all in liver**)	[150]
Human	Causes not stated	Cirrhosis	^1^H NMR	Acetate**↑** Pyruvate**↑** Glutamine**↑** α-Ketoglutarate**↑** Taurine**↑** Glycerol**↑** Tyrosine**↑** 1-Methylhistidine**↑** Phenylalanine**↑** *N*-Acetylglycoproteins**↑** LDL↓ VLDL↓ Isoleucine↓ Leucine↓ Valine↓ Acetoacetate↓ Choline↓ (**all in serum**)	[151]
Human	Alcohol	Cirrhosis (mild vs. severe liver failure)	^1^H NMR	Correlated with severity of liver failure: 3-Hydroxybutyrate**↑** Alanine**↑** Acetate**↑** Choline/Phosphocholine**↑** (**all in serum**)	[152]
Human	Chronic hepatitis B	Cirrhosis (compensated vs. decompensated)	^1^H NMR	Distinguishing between compensated and decompensated cirrhosis: Succinate, Pyruvate, Phenylalanine, Histidine, Lysine, Glutamine, Acetone, Glutamate, Creatine, Alanine (**all in serum**)	[153]
Human	Causes not stated	Cirrhosis	^1^H NMR	Positively correlated with portal blood proinflammatory cytokines IL6, TNFα and IL1β: Trimethylamine Negatively correlated with portal blood proinflammatory cytokines IL6, TNFα and IL1β: Acetate, n-Heptanoate Positively correlated with WBC and platelet counts: Threonine, α-Galactose, β-Glucose (**all in feces**)	[156]
Human	Various liver injuries	Cirrhosis (compensated vs. decompensated)	UPLC-QTOFMS	Lower in LC: *N*^6^-Methyladenosine, 1-Methyluric acid, Cinnamic acid, Decenoylcarnitine, Phenacetylglutamine (**all in urine**)	[158]
Human	Various etiologies, incl. HBV, HCV, alcohol, NASH	Cirrhosis US vs. Turkish (TR) population (dietary)	^1^H NMR	Lactate (Controls and Decompensated; TR>US), Glucose (Controls and Decompensated; US>TR ) (**all in plasma**)	[159]
Human	Alcohol	Acute-on-chronic liver failure (ACLF) vs. stable compensated or decompensated cirrhosis (CLF)	^1^H NMR	ACLF > CLF: 3-Hydroxybutyrate, Lactate, Acetoacetate, Pyruvate, Glutamine, Glutamate, Creatinine, Tyrosine, Phenylalanine (**all in serum**)	[160]
Human	Causes not stated	Stable cirrhosis (C) ± encephalopathy (E) (C±E) and HV	^1^H NMR	C±E > HV: Lactate, Pyruvate, Alanine, Threonine, Glycine, Aspartate, Acetoacetate, 3-Hydroxybutyrate, Phenylalanine, Tyrosine, Methionine, Glutamate, Methylamine, Dimethylamine, TMAO, Glycerol C±E < HV: Valine, Glutamine, Histidine, Arginine E > HV: Leucine, Isoleucine C > HV: Myoinositol (**all in plasma**)	[161]
Human	Alcohol, HBV or HCV, NASH	Hepatic encephalopathy (HE), cirrhosis (C), neurological patients without liver disease (NP), HV	UPLC-Orbitrap MS	HE > NP: 13x *N*-Acetyl metabolites, 5x Glutamate/Glutamine metabolites, 4x Methionine metabolites, 4x Phenylalanine metabolites, 6x Tryptophan metabolites, 6x Fatty acid metabolites, Pyruvate, 5x Amino acid derivatives, 2x Dipeptides, 3x Bile acids, 3x Nucleoside derivatives, Dihydrothymine, 4x Alcohols and polyols, Ribitol/Arabitol, Cortisol, Pyridoxic acid, Phenyl sulfate HE < NP: Alanine, Taurine, Anhydro sorbitol, Levulinic acid (**both in CSF and plasma**) HE > C: 9x *N*-Acetyl metabolites, Phenacetylglutamine, 2x Methionine metabolites, 2x Phenylalanine metabolites, 3x Tryptophan metabolites, 4x Fatty acid metabolites, Citrulline, 2x Dipeptides, Taurocholic acid, 3x Nucleosides and derivatives, Anhydro sorbitol, 2x Alcohols, Ribitol/Arabitol, Cortisol, Phenyl sulfate HE < C: Methionine sulfoxide, Levulinic acid (**all in plasma**)	[162]
Human	Chronic hepatitis C	Fibrosis (F4 vs. F0)	^1^H NMR	F4 vs. F0: VLDL**↑** Citrate**↑** Glucose**↑** Phenylalanine**↑** LDL↓ HDL↓ Choline↓ Acetoacetate↓ Isoleucine/Leucine↓ Creatinine/Creatine↓ Glutamate↓ Glutamine↓ Asparagine↓ Valine↓ Lysine↓ Cysteine↓ Glycerol↓ Arginine↓ Histidine↓ 3-Hydroxybutyrate↓ (**all in serum**)	[163]
Rat	TAA	Fibrosis/Cirrhosis vs. controls	^1^H NMR	3-Hydroxybutyrate**↑** Acetoacetate**↑** Butyrate**↑** Choline**↑** Glycine**↑** Alanine**↑** Leucine**↑** Lysine**↑** Succinate**↑** Valine**↑** 2-Oxoglutarate↓ Acetate↓ Adipate↓ Dimethylglycine↓ Lactate↓ Pyruvate↓ TMAO↓ Tyrosine↓ (**all in serum**) 1-Methylhistidine**↑** 3-Hydroxybutyrate**↑** Acetate**↑** Alanine**↑** Butyrate**↑** Choline**↑** Creatinine**↑** Hippurate**↑** Isoleucine**↑** Pyruvate**↑** Succinate**↑** Taurine**↑** TMAO**↑** Tryptophan**↑** Valine**↑** 2-Hydroxybutyrate↓ 2-Oxoglutarate↓ Acetoacetate↓ Acetone↓ Adipate↓ Citrate↓ Dimethylamine↓ Dimethylglycine↓ Fumarate↓ Methylamine↓ Oxaloacetate↓ Sarcosine↓ Trimethylamine↓ (**all in urine**)	[165]
Rat	Dimethylnitrosamine	Fibrosis	UPLC-QTOFMS	LPC(18:1) **↑** LPC(18:2) **↑** LPC(20:4)↓ FA(22:6)↓ FA(20:4)↓ FA(18:1)↓ FA(18:2)↓ (**all in serum**)	[166]
Rat	Dimethylnitrosamine	Fibrosis	UPLC-QTOFMS	Cholic acid**↑** Deoxycholic acid**↑** Ursodeoxycholic acid**↑** Chenodeoxycholic acid**↑** Hyodeoxycholic acid**↑** Lithocholic acid**↑** Taurocholic acid**↑** Taurodeoxycholic acid**↑** Tauroursodeoxycholic acid**↑** Taurochenodeoxycholic acid**↑** Taurohyodeoxycholic acid**↑** Taurolithocholic acid**↑** Glycocholic acid**↑** Glycodeoxycholic acid**↑** Glycoursodeoxycholic acid**↑** Glycochenodeoxycholic acid**↑** (**all in serum**)	[167]
Rat	CCl_4_	Fibrosis	UPLC-QTOFMS	Cervonoyl ethanolamide**↑** β-Muricholic acid**↑** (**all in serum**)	[168]
Rat	CCl_4_	Decompensated cirrhosis/ascites	UPLC-Orbitrap MS	Alanine**↑** Phenylalanine**↑** Tryptophan**↑** Tyrosine**↑** Nutriacholic acid**↑** LPC(16:0)↓ LPC(18:0)↓ LPC(18:2)↓ FA(20:5)↓ Carnitine↓ Creatine↓ Valine↓ Isoleucine↓ Arginine↓ (**all in serum**) Glutamyltaurine**↑** 4,6-Dihydroxyquinoline**↑** Phenylalanine**↑** TMAO**↑** 3-Methyldioxyindole**↑** 1,2,3-Trihydroxybenzene**↑** Tryptophan**↑** Histamine**↑** Tyrosine**↑** Pantothenic acid**↑** 2-Phenylglycine**↑** Proline**↑** *N*^6^,*N*^6^,*N*^6^-Trimethyllysine**↑** Dopamine**↑** Phenacetylglycine↓ Creatinine↓ Creatine↓ 4-Acetamidobutanoate↓ Indole↓ Carnitine↓ (**all in urine**)	[169]
Rat	CCl_4_	Fibrosis	UPLC-QTOFMS	12-Ketochenodeoxycholic acid**↑** PI(18:0/16:0) **↑** Cervonoyl ethanolamide**↑** LPC(18:2)**↑** LPC(22:6)**↑** PC(18:1/16:0)**↑** PC(18:2/16:0)**↑** PC(20:4/18:2)**↑** PC(22:6/18:1)**↑** Creatine↓ Sphinganine↓ Dihydroceramide↓ 8-HETE↓ LPC(18:0)↓ LPC(20:1)↓ LPC(22:0)↓ (**all in serum**)	[170]
Rat	Dimethylnitrosamine	Fibrosis	UPLC-Orbitrap MS	Leucine↓ LPC(16:0)↓ LPC(16:0)↓ LPC(18:0)↓ LPC(20:1)↓ LPC(20:4)↓ LPC(22:6)↓ FA(16:0)↓ FA(18:0)↓ FA(20:4)↓ FA(20:5)↓ FA(22:6)↓ All-*trans*-retinoic acid↓ Bilirubin↓ (**all in serum**)	[172]
Rat	CCl_4_	Fibrosis	^1^H NMR	2-Oxoglutarate↓ Citrate↓ Dimethylamine↓ Creatinine↓ Phenacetylglycine↓ Hippurate↓ Taurine**↑** (**all in urine**)	[123]
Rat	CCl_4_	Fibrosis	^1^H NMR	Glucose↓ Lactate**↑** Fumarate↓ NADPH↓ Succinate**↑** Acetate**↑** 3-Hydroxybutyrate↓ UDP-glucose**↑** UDP-galactose**↑** (**in serum and liver**)	[125]

**Table 4:** Metabolomic and lipidomic biomarkers of NAFL and NASH.

Species	Manipulation/Condition	Pathology	Analytical Methodology	Metabolites Reported	Ref.
Human	Obesity/metabolic syndrome	NAFL and NASH NAFL→NASH	HPLC-TQMS	FA(14:0)**↑** FA(16:0)**↑** FA(14:1n5)**↑** FA(16:1n7)**↑** FA(18:2n6)↓ FA(18:3n6)**↑** FA(20:3n6)**↑** FA(22:6n3)/FA(22:5n3) in PC and PE pools↓ 5-HETE**↑** 8-HETE**↑** 15-HETE**↑** (**all in plasma**)	[178]
Human	Nondiabetic. NAFLD confirmed by liver biopsy	NAFLD vs. HV	UPLC-TQMS GC-MS	Glycocholate**↑** Taurocholate**↑** Glycochenodeoxycholate**↑** Homocysteine**↑** Cysteine**↑** GSH↓ Glutamylvaline**↑** γ-Glutamylleucine**↑** γ-Glutamylphenylalanine**↑** γ-Glutamyltyrosine**↑** Cysteine-glutathione-disulfide↓ Carnitine**↑** Propionylcarnitine**↑** 2-Methylbutanoylcarnitine**↑** Butanoylcarnitine**↑** Tyrosine**↑** Glutamate**↑** Isoleucine**↑** Leucine**↑** Valine**↑** Taurocholate**↑** (**all in plasma**)	[180]
Human	Liver samples from normal (17), steatosis (4), NASH (fatty) (14) and NASH (not fatty) (23)	NAFL→NASH	UPLC Orbitrap-MS	Taurocholate**↑** Taurodeoxycholate**↑** Glycochenodeoxycholate**↑** Taurine**↑** Cholic acid↓ Glycodeoxycholate↓ (**all in liver**) Gene expression data consistent with the above (*CYP7B1***↑**)	[222]
Human	Dietary intervention study, unrelated healthy surgical liver samples	NAFL (20 insulin-resistant/20 insulin sensitive) vs. control	UPLC-TQMS GC-MS	Insulin-resistant NAFL vs. insulin-sensitive NAFL: Total LPCs↓ LPC(16:0)↓ (**all in plasma**)	[182]
Human	NASH and healthy subjects given high-fat meal to stimulate gall bladder contraction	Fasting and postprandial serum from NASH and healthy subjects	UPLC-TQMS	NASH vs. control (preprandial): Total BAs**↑** Glyco-BAs**↑** Tauro-BAs**↑** NASH vs. control (postprandial): Mainly Total BAs**↑** Glyco-BAs**↑** (**all in serum**)	[183]
Human	Normal, Steatosis, NASH with steatosis, NASH without steatosis livers	Normal, NAFL, fatty NASH, nonfatty-NASH	UPLC Orbitrap-MS	Control→NAFL: Acetyl carnitine**↑** Lauroyl carnitine**↑** Butanoyl carnitine**↑** Palmitoyl carnitine**↑** Hexanoyl carnitine↓ Valine↓ NAFL→NASH: Leucine**↑** Isoleucine **↑** Tyrosine**↑** Valine**↑** Phenylalanine**↑** (**all in liver**)	[184]
Human	Biopsy-proven NAFL, biopsy-proven NASH and normal controls with MRI fat fraction <5%	Normal, NAFL and NASH	UPLC-QTRAP MS/MS	NAFL→NASH: PGE2**↑** 13,14-dihydro-15-keto-PGD2**↑** 11,12-diHETrE**↑** 14,15-diHETrE**↑** 15-HETE↓ [all AA-derived] (**all in plasma**)	[185]
Human	Obese normal liver, obese NAFL and obese NASH	Normal, NAFL and NASH	^1^H NMR	LDL-cholesterol**↑** Alanine**↑** Histidine**↑** Phenylalanine**↑** Tyrosine**↑** Leucine**↑** Free fatty acids↓ Citrate↓ 3-Hydroxybutyrate↓ Acetoacetate↓ (**all in serum**)	[186]
Human	Morbid obesity with and without NAFL	Obesity without NAFL, mild NAFL, moderate NAFL, severe NAFL	UPLC-LITMS GC-TOFMS Metabolon, Inc.	α-Ketoglutarate principal plasma marker with AUROC of 0.743, sensitivity of 80%, specificity of 62.5%. (**all in plasma**)	[187]
Human Mouse	Liver biopsies from patients with normal liver and NAFLD HFD, HFD + nicotinic acid, HFD + hydroquinone, HFD + *tert*-butylhydroquinone	NASH vs. NAFL vs. control	GC-MS	Nicotinic acid and hydroquinone negatively correlated with steatosis (NAS) score. Nicotinic acid supplementation of HFD prevented fat accumulation and improved serum ALT. (**all in liver**)	[204]
Human	NAFLD, NAFLD + T2DM, control, evaluated by ultrasound	NAFLD, NAFLD + T2DM, control	UPLC-QTOFMS	NAFLD vs. control: Proline**↑** Phenylalanine**↑** Oleamide**↑** Bilirubin**↑** Palmitoyl carnitine**↑** LPC(20:5)**↑** Lyso-PAF C-18↓ NAFLD + T2DM vs. control: Leucine**↑** Oleamide**↑** LPC(14:0)**↑** Bilirubin**↑** Tetradecenoyl carnitine**↑** Linoleoyl carnitine**↑** Tetradecadienoyl carnitine**↑** (all in serum)	[206]
Human	Hyperuricemia (HU), HU+NAFLD, HU progressed to HU+NAFLD, healthy controls	HU, initial HU+NAFLD, initial HU→outcome HU+NAFLD, healthy controls	UPLC-QTOFMS	HU vs. control: Phosphatidic acid**↑** 3,4-Dihydroxyphenylglycol**↑** Valine**↑** CE(18:0)**↑** Uric acid**↑** Acetyl carnitine**↑** Inosine↓ 5-Hydroxyindoleacetic acid↓ 5-Aminoimidazole ribotide↓ Pyrrolidonecarboxylic acid↓ Glycerophosphocholine↓ HU vs. outcome HU+NAFLD: Phosphatidic acid**↑** Inosinic acid**↑** Tryptophan**↑** Valine**↑** Alanine**↑** Lactate**↑** CE(18:0)**↑** Uric acid**↑** Trimethylamine**↑** Acetyl carnitine**↑** 5-Methoxyindoleacetic acid**↑** Acetoin**↑** Inosine↓ Kynurenine↓ 5-Hydroxyindoleacetic acid↓ Pyrrolidonecarboxylic acid↓ 4-Fumarylacetoacetate↓ Pregnenolone sulfate↓ (**all in serum**)	[203]
Human	Bariatric surgery patients with wedge liver biopsy during surgery classified histologically as non-NASH, non-NAFLD, NAFL and NASH. *PNPLA3* I148M variant also determined (more common in NASH). Discovery and validation cohorts used.	non-NASH vs. non-NAFLD vs. NAFL vs. NASH	UPLC-QTOFMS 2D-GC-TOFMS	Strong negative correlation between number of TG double bonds to TG concentrations in NASH relative to non-NASH for both discovery and validation cohorts. A “NASH ClinLipMet score” was developed based upon (i) clinical variables, (ii) *PNPLA3* genotype, (iii) lipidomic data and (iv) metabolomic data. This was highest performing combination biomarker with sensitivity of 85.5% and specificity of 72.1% for NASH. (**all in liver**)	[188]
Human	Normal liver, NAFL liver, NASH liver	NASH vs. NAFLD vs. control lipidomics	UPLC-TQMS GC-MS	Thirty-two lipids discriminated NASH with 100% sensitivity and specificity. Accumulated hepatotoxic lipids in NASH included FA(14:0), FA(16:0), FA(16:1n-7), FA(18:1n-7) and FA(18:1n-9). Reduced in NASH: FA(20:4n-6), FA(20:5n-3), FA(22:6n-3), total CER, total SM, total PI, total PS, total PE, total PC. (**all in liver**)	[189]
Human	Nondiabetic NAFL patients with normal liver function, NASH with abnormal liver function, healthy controls.	NASH vs. NAFL vs. control urines.	LC-TQMS	NASH vs. control: Lysine**↑** Valine**↑** Citrulline**↑** Arginine**↑** Threonine**↑** Tyrosine**↑** Leucine**↑** Hippurate**↑** 3-Indoleacetate**↑** 5-Hydroxyindoleacetate↓ 3-Indoleformate↓ Cortisol↓ NASH vs. NAFL: Methyl xanthine**↑** Tryptophan**↑** 3-Indoleacetate**↑** Gluconate**↑** Proline↓ (**all in urine**)	[190]
Human	Several large clinical cohorts with CT-defined liver fat plus NASH patients.	NASH vs. controls	UPLC-Q-Orbitrap-MS	Top metabolite correlated with liver fat was 202.1185^+^, which produced 24 hits in HMDB. Dimethylguanidino valeric acid (DMGV) chosen on basis of GWAS, which found SNPs for AGXT2 that produces DMGV. (**all in plasma**)	[191]
Human	NAFLD criteria met/not met at baseline, after dietary manipulation.	Non-NAFLD, Non-NAFLD→NAFLD, NAFLD→Non-NAFLD	UPLC-QTOFMS	Phospholipid and sphingolipid changes not of great statistical significance. Also lipid groups, not individual lipids, given only. (**all in serum**)	[241]
Human	NAFL and NASH based upon liver biopsy, healthy controls.	NASH vs. NAFL vs. controls	HPLC-Orbitrap-MS	Five metabolites increased control→NAFL→NASH – Uracil, α-Linolenic acid (all-*cis*-9,12,15-octadecatrienoic acid), Glutamate, Glutamine and 5-Oxoproline, which was chosen as a biomarker with a better AUROC for NASH vs. NAFL, than adiponectin, TNF-α, or IL-8. (**all in serum**)	[192]
Human	NAFL and NASH confirmed by liver biopsy	NASH vs. NAFL	High-field ^1^H MRS and ultra-high-field ^31^P MRS (*in vivo*)	Many MRS alterations correlated with NAFL→NASH, mostly with advanced fibrosis, e.g. phosphoethanolamine/total phosphorus (TP) ratio. ATP/TP↓ in advanced fibrosis and ATP flux↓ in NASH	[155]
Human	Chronic hepatitis B (CHB) with biopsy-proven NAFLD and without NAFLD, healthy controls	CHB +NAFLD vs. CHB-NAFLD vs. controls	UPLC-QTOFMS	Most neutral lipids and ceramides were elevated in CHB+NAFLD but decreased in CHB-NAFLD vs. healthy controls. Monounsaturated TGs were a good predictor of NASH, superior to cytokeratin-18 or ALT.	[196]
Human	Hepatic steatosis in morbidly obese women	Metagenomic signature of hepatic steatosis	^1^H NMR (urine and plasma) UPLC-TQMS (plasma)	Microbiota metabolite produced from phenylalanine, phenylacetic acid (PAA) associated with steatosis.	[197]
Human	Biopsy-proven subjects with normal liver (NL), NAFL and NASH	NASH vs. NAFL vs. NL discovery and validation cohorts	UPLC-QTOFMS	Triglycerides are elevated NAFL > NL ≥ NASH. Of the 28 TGs measured, TG(46:0), (48:0), (53:0), (44:1), (48:1), (49:1), (52:1), (53:1), (50:2), (54:5) and (58:2) were always NAFL > NL and NASH < NAFL. (**all in serum**)	[198]
Human	Large study of 769 nondiabetic patients with liver fat content measured by MRI and correlated with metabolite profiles of urine and fasting plasma	613 plasma and 587 urine samples across a range of liver pathologies (34.7% with steatosis)	UPLC-LITMS ^1^H NMR	Associations in plasma with LFC: BCAAs**↑** Aromatic amino acids**↑** Dipeptides**↑** Proline**↑** Tryptophan**↑** Indoleacetate**↑** Urate**↑** Piperine**↑** 7α-Hydroxy-3-oxo-cholestenoate**↑** Ether-PCs↓ 3-Phenylpropionate↓ Proline betaine↓ Associations in urine with LFC: BCAA derivatives**↑** Lactate**↑** Isovalerylglycine↓ Isobutyrylglycine↓ γ-Glutamylthreonine↓ 4-Vinylphenol sulfate↓ Hippurate↓ Cinnamoylglycine↓	[200]
Human	NAFLD determined by hepatic ultrasound	BMI < 25 vs. BMI > 30 vs. BMI > 30 with NAFLD	TQMS for 31 acyl carnitines and 7 amino acids	Family history predicted obesity correlating with amino acids that contributed to an increase in specific acyl carnitines. Excess FFAs related to obesity were associated with NAFLD.	[201]
Human	Patients with normal fasting glucose. Visceral adipose tissue (VAT) assessed by MRI. Hepatic TG content (HTGC) determined by proton-MR spectroscopy.	Range of VAT and HTGC	ESI-FIA-MS/MS	Associated with HTGC: LPC(14:0), PC(28:1), PC(30:0), PC(32:1), PC(32:2), PC(34:1), PC(34:3), PC(34:4), PC(36:1), PC(36:2), PC(36:3), PC(36:6), PC(38:3), PC(38:5), PC(40:4), PC(40:5), SM(22:3), Tryptophan, Tyrosine (**all in plasma**)	[202]
Human	NAFL and NASH determined by liver biopsy and healthy controls by ultrasound and liver enzymes	NASH vs. NAFL vs. controls	UPLC-Orbitrap-MS GC	Lipid group trends: DG: NASH > NAFL > healthy PG: NASH ≈ NAFL > healthy PA: NASH ≈ NAFL > healthy AcCa: NASH < NAFL ≈ healthy CE: NASH < NAFL < healthy LPC: NASH < NAFL ≈ healthy SM: NASH < NAFL ≈ healthy FA(16:0): NASH > NAFL > healthy FA(16:1n-7*cis*): NASH > NAFL > healthy FA(18:1n-9*cis*): NASH > NAFL > healthy FA(18:2n-6): NASH < NAFL < healthy FA(20:4n-6): NASH < NAFL < healthy	[199]
Human	Children, overweight or obese, with or without clinical/radiological signs of NAFLD	NAFLD vs. control	Selective ion flow tube mass spectrometry (SIFT-MS)	Acetaldehyde**↑** Acetone**↑** Isoprene**↑** Pentane**↑** Trimethylamine**↑** (**all in breath**)	[209]
Human	Children with biopsy-proven NAFLD and matched healthy controls	NAFLD vs. control	HPLC	Homocysteine**↑** Cysteine**↑** CysGly**↑** GSH↓ (**all in plasma**)	[211]
Human	Children with obesity and NAFL confirmed by MRS and matched obese controls	NAFL vs. control	UPLC-Q-Orbitrap-MS	Tyrosine**↑** Glutamate**↑** Octanoic acid**↑** Linoleic acid↓ (**all in plasma**)	[212]
Human	Children with obesity, NAFL, NASH and healthy controls	NASH vs. NAFL vs. control	GC-MS	1-Butanol**↑** (in NAFL) 1-Pentanol**↑** (in NAFL) ↓ (in NASH) Phenol**↑** (in NAFL) 2-Butanone**↑** (in NAFL and NASH) 4-Methyl-2-pentanone↓ (in NAFL)**↑** (in NASH) (**all in feces**) Metagenomics also conducted. Correlations with NAFLD and certain VOCs reported	[242]
Human	Children with NAFLD, with or without obesity	Obese − NAFL, obese + NAFL, normal weight healthy controls	GC-MS	NAFL vs. control: Glucose**↑** 1-Methylhistidine**↑** Pseudouridine**↑** Glycolic acid**↑** Mannose↓ *p*-Cresol sulfate↓ Kynurenine↓ Hydroquinone↓ Adipate↓ Phenylacetic acid↓ Small intestine bacterial overgrowth (SIBO): Glycolic acid**↑** Mannose**↑** Valine↓ *p*-Cresol sulfate↓ Butanoate↓ Adipate↓ (**all in urine**)	[213]
Human	Children with obesity, with and without NAFLD assessed by MRI	Obese + NAFLD vs. obese − NAFLD	UPLC-QTRAP-MS	Isoleucine**↑** Leucine**↑** Valine**↑** C4-carnitine**↑** C5-carnitine**↑** C14:1-OH-carnitine**↑** Tryptophan**↑** Lysine**↑** Glutamate**↑** PC(32:1)**↑** (**all in plasma**)	[214]
Human	Children with obesity, with and without NAFL assessed by ultrasound (US), with and without metabolic syndrome (MetS) and nonobese controls	Obese + NAFL vs. obese − NAFL Obese + MetS vs. obese − MetS	GC-MS	Obese − NAFL vs. controls: Palmitate**↑** Myristate**↑** Urea**↑** *N*-Acetylgalactosamine**↑** Maltose**↑** Gluconate**↑** Isoleucine**↑** Hydroxybutanoate↓ Malate↓ Obese + NAFL vs. controls: Laurate**↑** Maltose**↑** (**all in saliva**)	[215]
Human	Adolescents with NAFLD assessed by US or ALT/AST	US vs. ALT vs. AST diagnostic methods	Biochemical lipid analysis ^1^H NMR	Many differences in lipid profiles, amino acids (alanine, glutamine, histidine; BCAAs; aromatic amino acids) and ketone bodies (acetate, acetoacetate, β-hydroxybutyrate) (**all in plasma**)	[216]
Human	Adolescents with obesity and with or without NAFLD confirmed by MRI	NAFLD vs. non-NAFLD	UPLC-Q-Orbitrap-MS	Leucine/Isoleucine**↑** Tryptophan**↑** Serine↓ Dihydrothymine↓ LPE(20:0)↓ LPC(18:1)↓ (**all in plasma**)	[217]
Human	Children with or without NAFLD confirmed by ultrasound and liver enzymes	NAFLD vs. non-NAFLD	GC-MS	24-h Urinary steroid profiles: Cortisol (obese controls)**↑** Tetrahydrocortisone (NAFLD)**↑** Overall data pointed to 5α-reductase**↑**, 21-hydroxylase**↑** and 11β-hydroxysteroid dehydrogenase 1↓	[243]
Human Mouse	Morbidly obese, nondiabetic *Gnmt*-null vs. WT	NAFL→NASH NASH vs. control	UPLC-QTOFMS	PC(14:0/20:4)**↑** LPC(18:1)**↑** PC(P-24:0/0:0)↓ PC(P-22:0/0:0)↓ PC(O-20:0/0:0)↓ FA(20:4)↓ Glutamate↓ (**all in serum**) Results consistent with human studies	[179]
Mouse Human	Methionine and choline deficient diet (MCD) HBV-negative, NAFLD confirmed by liver biopsy	NASH vs. NAFL vs. control	^1^H NMR	Glucose**↑** Lactate**↑** Glutamate**↑** Taurine**↑** TG**↑** Total cholesterol**↑** LDL cholesterol**↑** Glucose**↑** Lactate**↑** Glutamate**↑** Taurine**↑** (**all in serum**)	[181]
Mouse Human	*Mat1a*-KO vs. WT mouse liver and serum metabolome NAFL and NASH discovery and validation cohorts	*Mat1a*-KO vs. WT Clustering analysis into M-subtype and Non-M-subtype based upon mouse metabolomes	UPLC-QTOFMS	M-subtype NASH biomarkers: Amino acids (5), Fatty acyls (8), Triglycerides (3), Glycerophospholipids (37), Sphingomyelins (1) Non-M-subtype NASH biomarkers: Amino acids (1), Fatty acids (1), Bile acids (1), Triglycerides (3) M-subtype patients: 34% NASH Non-M-subtype patients: 39% NASH	[205]
Mouse	MCD	NASH vs. control	UPLC-QTOFMS	Tauro-β-muricholate**↑** Taurocholate**↑** 12-HETE**↑** LPC(16:0)↓ LPC(18:0)↓ LPC(18:1)↓ (**all in serum**)	[218]
Mouse	MCD vs. choline-supplemented MCD (MCS)	Differential effects of methionine and choline deficiency	UPLC-QTOFMS	MCD vs MCS: Oleic acid**↑** Linoleic acid**↑** Total nonesterified fatty acids**↑** (**all in serum**)	[219]
Mouse	NASH-inducing diet (35% lard, 1.25% cholesterol, 0.5% sodium cholate)	NAFLD vs. control	HPLC-TQMS	Glycerol**↑** Free cholesterol**↑** Esterified cholesterol**↑** Putrescine**↑** *N*^8^-Acetylspermidine↓ Spermine↓ Adenine↓ Adenosine↓ Homocysteine↓ Methylthioadenosine↓ *S*-Adenosylhomocysteine↓ *S*-Adenosylmethionine↓ Proteomic findings were consistent with the above (**all in liver**)	[220]
Mouse	*Ldlr*-null mice fed a Western diet (energy as 17% protein, 43% carbohydrate, 41% fat, 0.2% cholesterol) + olive oil (WD + O)	NAFLD/NASH vs. control	UPLC-LITMS GC-MS Metabolon, Inc.	Saturated fatty acids**↑** MUFAs**↑** Palmitoyl-sphingomyelin**↑** Cholesterol**↑** n-6 PUFA**↑** 12-HETE**↑** C_20-22_ n-3 PUFA-containing phosphoglycerolipids↓ 18-HEPE↓ 17,18-diHETE↓ *S*-Lactoyl-glutathione↓ (**all in liver**) F3-Isoprostanes↓ (**in urine**)	[221]
Mouse	HFD (60% calories from fat) and normal chow (12.7% calories from fat)	NAFLD vs. control	^1^H NMR	Glucose**↑** Total cholesterol**↑** HDL-cholesterol**↑** AST**↑** ALT**↑** Phosphatidylcholine**↑** Pyruvate↓ Acetate↓ Lactate↓ Citrate↓ Arginine↓ Ornithine↓ Acetoacetate↓ 3-Hydroxybutyrate↓ Isoleucine↓ Leucine↓ Valine↓ Glutamate↓ Glutamine↓ Tyrosine↓ Phenylalanine↓ Alanine↓ Lysine↓ Glycine↓ Betaine↓ Isobutanoate↓ 1-Methylhistidine↓ (**all in serum**) Total cholesterol**↑** Triglycerides**↑** Fatty acids**↑** PUFA/MUFA↓ (**all in liver**) Pyruvate**↑** Creatinine**↑** Taurine**↑** Glycine**↑** Formate**↑** Butanoate**↑** Guanidinoacetate**↑** Glucose**↑** 1-Methylnicotinamide**↑** Nicotinamide *N*-oxide**↑** Acetoacetate↓ Succinate↓ Citrate↓ 2-Oxoglutarate↓ Trimethylamine↓ *Trans*-aconitate↓ Hippurate↓ Trigonelline↓ Niacinamide↓ Tyrosine↓ 1-Methylhistidine↓ Phenylalanine↓ (**all in urine**)	[223]
Mouse	HFD (60% calories from fat) and normal chow (13.5% calories from fat)	NAFLD vs. control	UPLC-QTOFMS GC-MS	Methylhippurate**↑** Glycerol 3-phosphate**↑** Mannose**↑** Ketoleucine**↑** 2-Ketohexanoate**↑** Hydroxyphenyllactate**↑** Succinate**↑** Xylose/Ribose/Arabinose↓ Glucuronate↓ Catechol↓ 4-Coumarate↓ Hippurate↓ Taurocholate↓ Glycochenodeoxycholate↓ Glycocholate↓ Histamine↓ (**all in serum**)	[224]
Mouse	A/J, C57BL/6J and PWD/PhJ strains fed standard diet with 0.1% DDC	DDC-treated vs. control	UPLC-Q-LITMS	Putrescine**↑** Arginine**↑** Citrulline**↑** cAMP**↑** 2-Oxoglutarate**↑** Asparagine**↑** Glutamate**↑** (**all in liver**)	[227]
Mouse	HFD-fed mice (42% calories from fat, 43% from carbohydrates, 15% from protein) vs. standard chow (17% from fat, 58% from carbohydrates, 25% from protein)	NAFLD vs. control	UPLC-Q-LITMS	SFA-DAGs**↑** MUFA-DAGs**↑** PUFA-DAGs↓ SFA-CEs**↑** MUFA-CEs**↑** PAs**↑** PGs**↑** SFA-CERs**↑** Sphingosine**↑** Sphingosine-1-phosphate**↑** Dihydrosphingosine**↑** Dihydrosphingosine-1-phosphate**↑** Galactosylceramide↓ Glucosylceramide↓ Lactosylceramide**↑** Globotrioseacylceramide**↑** TxB2**↑** PGF2α**↑** All other eicosanoids↓ Pattern changed from weeks 16-52. (**all in liver**)	[228]
Mouse	db/db leptin receptor-deficient mice with insulin resistance and steatosis subjected to caloric restriction (CR), db/m mice without insulin resistance and steatosis	db/db, pre- and post-caloric restriction, db/m	^1^H NMR UPLC-QTOFMS	db/db vs. db/m: Acetone**↑** 3-Hydroxybutyrate**↑** Lactate**↑** Acetate**↑** Glutathione**↑** Ascorbate**↑** Many glycerolipids**↑** db/db + CR vs. db/db: 3-Hydroxybutyrate↓ Ascorbate↓ Many glycerolipids↓ RT-PCR findings consistent with metabolomic data (**all in liver**)	[231]
Mouse	Leptin-deficient obese *ob*/*ob* mice and nonsteatotic *ob*/+ heterozygous mice	Intact liver tissues of two mouse lines compared	HR-MAS ^1^H NMR	Many lipid ^1^H signals highly statistically significantly elevated in steatotic *ob*/*ob* livers compared with nonsteatotic *ob*/+ livers, as expected. *ob*/*ob* livers vs. *ob*/+ livers: Betaine**↑** Phenylalanine**↑** Uridine**↑** Creatinine↓ Glutamate↓ Glycine↓ Glycolate↓ Trimethylamine *N*-oxide↓ *N*,*N*-Dimethylglycine↓ ADP↓ AMP↓	[232]
Mouse	High-*trans*-fat high-fructose diet (TFD) for 8 weeks (steatosis) and 24 weeks (NASH)	TFD-fed, normal diet-fed	Fasting hepatic mitochondrial flux by ^13^C NMR isotopomer analysis. LC-TQMS lipidomics	8-week (steatosis) vs. 24-week (NASH): Endogenous glucose production**↑** TCA cycle flux**↑** Anaplerosis**↑** Pyruvate cycling**↑** Control vs. 8-week (steatosis): Total diacylglycerols**↑** Total ceramides**↑** C8-acyl carnitine**↑** C16-acyl carnitine**↑** 8-week (steatosis) vs. 24-week (NASH): DG(16:1/16:1)**↑** DG(16:0/18:1)**↑** DG(34:2)**↑** DG(18:1/18:1)**↑** DG(18:1/18:2)**↑** DG(18:2/18:2)**↑** DG(16:0/20:4)**↑** DG(18:0/20:4)**↑** DG(18:1/20:4)**↑** DG(18:2/20:4)**↑** DG(18:2/20:2)**↑** DG(16:1/22:6)**↑** DG(18:1/22:6)**↑** C6-acyl carnitine**↑** C8-acyl carnitine**↑** C14-acyl carnitine**↑** C16-acyl carnitine**↑** CER(20:0)↓ CER(22:0)↓ (**all in liver**)	[229]
Mouse	High-fat, high-cholesterol, cholate (HFDCC)-fed mice with NAFLD without obesity	HFDCC vs. control	GC-TOFMS UPLC-QTOFMS	Total cholesterol**↑** CE(16:1), (18:1), (18:2), (18:3), (20:1), (20:3), (20:4), (22:5), (22:6)**↑** Cholic acid**↑** DGs**↑** TGs**↑** CERs**↑** SMs**↑** LPCs**↑** PC/PE**↑** PEs↓ Xylitol**↑** Xanthosine**↑** Squalene**↑** Phenylethylamine**↑** Citrate↓ G-1-P↓ Saccharic acid↓ (**all in liver**) Total cholesterol**↑** CE(16:1), (18:1), (18:2), (18:3), (20:1), (20:3), (20:4), (22:5)**↑** Cholic acid**↑** Deoxycholic acid**↑** CERs**↑** SMs**↑** PEs**↑** FFAs↓ Glycerol↓ TGs↓ LPEs↓ (**all in plasma**)	[225]
Rat	HFD-induced NASH	HFD vs. control diet	UPLC-QTOFMS	Glucose**↑** Triglycerides**↑** LDL-cholesterol**↑** SM(36:1)**↑** LPC(18:1)**↑** LPC(20:2)**↑** SM(34:2)**↑** PC(34:1)**↑** PC(38:4)**↑** PC(38:3)**↑** LPC(17:1)**↑** PC(35:2)**↑** FA(20:1)**↑** FA(20:3)**↑** FA(22:3)**↑** Phytomonate**↑** LPC(14:0)**↑** 13-HpODE**↑** PC(37:4)↓ PC(38:4)↓ PC(38:6)↓ SM(34:1)↓ SM(34:2)↓SM(42:3)↓ SM(40:1)↓ PC(40:5)↓ PC(40:6)↓ PC(40:8)↓ Creatine↓ Indoxyl sulfate↓ (**all in serum**)	[233]
Rat	HFD-induced NASH, positive controls (methionine plus choline supplementation), control diet	HFD vs. positive control vs. control diet	UPLC-QTOFMS	HFD vs. control: FA(28:8)**↑** CE(12:0)**↑** PG(14:0/18:1)**↑** Cortisone↓ Antrosta-1,4-diene-3,17-dione↓ All-*trans*-retinoyl-β-glucuronide↓ LPA(18:2)↓ PE(15:0/22:2)↓ Cortol↓ 21-Hydroxypregnenolone↓ Cortolone↓ Urobilin↓ LPA(18:1)↓ PA(P-20:0/14:0)↓ (**all in serum**)	[234]
Rat Human	Rats fed HFD to lead to steatosis, rats fed MCD diet to lead to NASH, rats fed methionine and choline sufficient diet as controls→liver samples NASH (fatty), NASH (not fatty), steatosis, healthy liver samples	NASH vs. NAFL vs. control (rat and human)	UPLC Orbitrap-MS	Bile acid metabolomics: Significant BA profile differences between rat MCD and human NASH. Amino acid metabolomics: Asparagine**↑** Citrulline**↑** Lysine**↑** comparable between rat MCD and human NASH. Fatty acid, carnitine and LPC metabolomics: Stearoyl carnitine**↑** only lipid in both rat MCD and human NASH	[237]
Rat	HFD, MCD diet and streptozocin (STZ) in rats. Metabolomics and transcriptomics on serum and liver.	NAFL vs. NASH vs. NAFL + T2DM	UPLC-QTOFMS	Venn diagram for HFD, MCD and HFD+STZ serum: Stearoyl carnitine**↑** (9*E*)-octadecenoyl carnitine**↑** docosapentaenoic acid**↑** vitamin D2**↑**	[238]
Rat	HFD/cholesterol diet vs. normal diet	Stage of steatosis, inflammation and fibrosis determined histologically	GC	Correlation between liver and blood cell total fatty acids for control diet: FA(16:1), FA(22:6), FA(18:1n-7), FA(22:5) Correlation between liver and blood cell total fatty acids for HFD/cholesterol diet: FA(22:6), FA(18:1n-7)	[239]
Rat	HFD vs. normal diet	NAFLD established by histology and liver enzymes	UPLC-TQMS	BAs in liver: Taurocholate**↑** Taurohyodeoxycholate↓ Ursodeoxycholate↓ BAs in caecal contents: Cholate**↑** Hyodeoxycholate↓ Muricholate↓ BAs in serum: Taurocholate**↑** Hyodeoxycholate↓ Taurohyodeoxycholate↓	[240]

## References

[1] Massarweh N.N., El-Serag H.B. (2017). Epidemiology of Hepatocellular Carcinoma and Intrahepatic Cholangiocarcinoma. Cancer Control.

[2] Severi T., van Malenstein H., Verslype C., van Pelt J.F. (2010). Tumor initiation and progression in hepatocellular carcinoma: Risk factors, classification, and therapeutic targets. Acta Pharmacol. Sin..

[3] Altekruse S.F., Devesa S.S., Dickie L.A., McGlynn K.A., Kleiner D.E. (2011). Histological classification of liver and intrahepatic bile duct cancers in SEER registries. J. Registry Manag..

[4] Baecker A., Liu X., La Vecchia C., Zhang Z.F. (2018). Worldwide incidence of hepatocellular carcinoma cases attributable to major risk factors. Eur. J. Cancer Prev..

[5] Uhlen M., Fagerberg L., Hallstrom B.M., Lindskog C., Oksvold P., Mardinoglu A., Sivertsson A., Kampf C., Sjostedt E., Asplund A. (2015). Proteomics. Tissue-based map of the human proteome. Science.

[6] Ben-Moshe S., Itzkovitz S. (2019). Spatial heterogeneity in the mammalian liver. Nat. Rev. Gastroenterol. Hepatol..

[7] Zhang D.Y., Goossens N., Guo J., Tsai M.C., Chou H.I., Altunkaynak C., Sangiovanni A., Iavarone M., Colombo M., Kobayashi M. (2016). A hepatic stellate cell gene expression signature associated with outcomes in hepatitis C cirrhosis and hepatocellular carcinoma after curative resection. Gut.

[8] Verhulst S., Roskams T., Sancho-Bru P., van Grunsven L.A. (2019). Meta-Analysis of Human and Mouse Biliary Epithelial Cell Gene Profiles. Cells.

[9] Aguilar-Bravo B., Sancho-Bru P. (2019). Laser capture microdissection: Techniques and applications in liver diseases. Hepatol. Int..

[10] Nicholson J.K., Lindon J.C., Holmes E. (1999). ‘Metabonomics’: Understanding the metabolic responses of living systems to pathophysiological stimuli via multivariate statistical analysis of biological NMR spectroscopic data. Xenobiotica.

[11] Idle J.R., Gonzalez F.J. (2007). Metabolomics. Cell Metab..

[12] Beyoglu D., Zhou Y., Chen C., Idle J.R. (2018). Mass isotopomer-guided decluttering of metabolomic data to visualize endogenous biomarkers of drug toxicity. Biochem. Pharmacol..

[13] Nicholson J.K., Lindon J.C. (2008). Systems biology: Metabonomics. Nature.

[14] Gika H., Virgiliou C., Theodoridis G., Plumb R.S., Wilson I.D. (2019). Untargeted LC/MS-based metabolic phenotyping (metabonomics/metabolomics): The state of the art. J. Chromatogr. B Anal. Technol. Biomed. Life Sci..

[15] Roy C., Tremblay P.Y., Bienvenu J.F., Ayotte P. (2016). Quantitative analysis of amino acids and acylcarnitines combined with untargeted metabolomics using ultra-high performance liquid chromatography and quadrupole time-of-flight mass spectrometry. J. Chromatogr. B Anal. Technol. Biomed. Life Sci..

[16] Sinclair K., Dudley E. (2019). Metabolomics and Biomarker Discovery. Adv. Exp. Med. Biol..

[17] Bartel J., Krumsiek J., Theis F.J. (2013). Statistical methods for the analysis of high-throughput metabolomics data. Comput. Struct. Biotechnol. J..

[18] Considine E.C., Thomas G., Boulesteix A.L., Khashan A.S., Kenny L.C. (2017). Critical review of reporting of the data analysis step in metabolomics. Metabolomics.

[19] Rosato A., Tenori L., Cascante M., De Atauri Carulla P.R., Martins Dos Santos V.A.P., Saccenti E. (2018). From correlation to causation: Analysis of metabolomics data using systems biology approaches. Metabolomics.

[20] Wishart D.S., Lewis M.J., Morrissey J.A., Flegel M.D., Jeroncic K., Xiong Y., Cheng D., Eisner R., Gautam B., Tzur D. (2008). The human cerebrospinal fluid metabolome. J. Chromatogr. B Anal. Technol. Biomed. Life Sci..

[21] Psychogios N., Hau D.D., Peng J., Guo A.C., Mandal R., Bouatra S., Sinelnikov I., Krishnamurthy R., Eisner R., Gautam B. (2011). The human serum metabolome. PLoS ONE.

[22] Bouatra S., Aziat F., Mandal R., Guo A.C., Wilson M.R., Knox C., Bjorndahl T.C., Krishnamurthy R., Saleem F., Liu P. (2013). The human urine metabolome. PLoS ONE.

[23] Karu N., Deng L., Slae M., Guo A.C., Sajed T., Huynh H., Wine E., Wishart D.S. (2018). A review on human fecal metabolomics: Methods, applications and the human fecal metabolome database. Anal. Chim. Acta.

[24] Wishart D.S., Feunang Y.D., Marcu A., Guo A.C., Liang K., Vazquez-Fresno R., Sajed T., Johnson D., Li C., Karu N. (2018). HMDB 4.0: The human metabolome database for 2018. Nucleic Acids Res..

[25] Scalbert A., Brennan L., Manach C., Andres-Lacueva C., Dragsted L.O., Draper J., Rappaport S.M., van der Hooft J.J., Wishart D.S. (2014). The food metabolome: A window over dietary exposure. Am. J. Clin. Nutr..

[26] Lydic T.A., Goo Y.H. (2018). Lipidomics unveils the complexity of the lipidome in metabolic diseases. Clin. Transl. Med..

[27] Garcia-Ortega L.F., Martinez O. (2015). How Many Genes Are Expressed in a Transcriptome? Estimation and Results for RNA-Seq. PLoS ONE.

[28] Wilhelm M., Schlegl J., Hahne H., Gholami A.M., Lieberenz M., Savitski M.M., Ziegler E., Butzmann L., Gessulat S., Marx H. (2014). Mass-spectrometry-based draft of the human proteome. Nature.

[29] Zanger U.M., Schwab M. (2013). Cytochrome P450 enzymes in drug metabolism: Regulation of gene expression, enzyme activities, and impact of genetic variation. Pharmacol. Ther..

[30] Aslebagh R., Wormwood K.L., Channaveerappa D., Wetie A.G.N., Woods A.G., Darie C.C. (2019). Identification of Posttranslational Modifications (PTMs) of Proteins by Mass Spectrometry. Adv. Exp. Med. Biol..

[31] Menetski J.P., Hoffmann S.C., Cush S.S., Kamphaus T.N., Austin C.P., Herrling P.L., Wagner J.A. (2019). The Foundation for the National Institutes of Health Biomarkers Consortium: Past Accomplishments and New Strategic Direction. Clin. Pharmacol. Ther..

[32] Booth J. (1977). A short history of blood pressure measurement. Proc. R. Soc. Med..

[33] Dobson M. (1776). Nature of the urine in diabetes. Med. Obs. Inqu..

[34] Garrod A.E. (1909). Inborn Errors of Metabolism.

[35] Garrod A.E. (1902). The incidence of alkaptonuria: A study in chemical individuality. Lancet.

[36] Piro A., Tagarelli G., Lagonia P., Quattrone A., Tagarelli A. (2010). Archibald Edward Garrod and alcaptonuria: “Inborn errors of metabolism” revisited. Genet. Med..

[37] Perlman R.L., Govindaraju D.R. (2016). Archibald E. Garrod: The father of precision medicine. Genet. Med..

[38] Phornphutkul C., Introne W.J., Perry M.B., Bernardini I., Murphey M.D., Fitzpatrick D.L., Anderson P.D., Huizing M., Anikster Y., Gerber L.H. (2002). Natural history of alkaptonuria. N. Engl. J. Med..

[39] Ranganath L.R., Khedr M., Milan A.M., Davison A.S., Hughes A.T., Usher J.L., Taylor S., Loftus N., Daroszewska A., West E. (2018). Nitisinone arrests ochronosis and decreases rate of progression of Alkaptonuria: Evaluation of the effect of nitisinone in the United Kingdom National Alkaptonuria Centre. Mol. Genet. Metab..

[40] Adinolfi M., Delves P.J. (1998). Embryonic antigens. Encyclopedia of Immunology.

[41] Abelev G.I., Perova S.D., Khramkova N.I., Postnikova Z.A., Irlin I.S. (1963). Production of embryonal alpha-globulin by transplantable mouse hepatomas. Transplantation.

[42] Chan S.L., Chan A.W.H., Yu S.C.H., Patel V.B., Preedy V.R. (2017). Alpha-Fetoprotein as a Biomarker in Hepatocellular Carcinoma: Focus on Its Role in Composition of Tumor Staging Systems and Monitoring of Treatment Response. Biomarkers in Disease: Methods, Discoveries and Applications.

[43] Wu M., Liu H., Liu Z., Liu C., Zhang A., Li N. (2018). Analysis of serum alpha-fetoprotein (AFP) and AFP-L3 levels by protein microarray. J. Int. Med. Res..

[44] Derosa G., Maffioli P., Patel V.B., Preedy V.R. (2017). Traditional markers in liver disease. Biomarkers in Disease: Methods, Discoveries and Applications.

[45] Lou J., Zhang L., Lv S., Zhang C., Jiang S. (2017). Biomarkers for Hepatocellular Carcinoma. Biomark. Cancer.

[46] Bruha R., Patel V.B., Preedy V.R. (2017). Osteopontin as a biomarker in liver disease. Biomarkers in Liver Disease: Methods, Discoveries and Applications.

[47] Crandall D.I., Halikis D.N. (1954). Homogentisic acid oxidase. I. Distribution in animal tissue and relation to tyrosine metabolism in rat kidney. J. Biol. Chem..

[48] Bernardini G., Laschi M., Geminiani M., Braconi D., Vannuccini E., Lupetti P., Manetti F., Millucci L., Santucci A. (2015). Homogentisate 1,2 dioxygenase is expressed in brain: Implications in alkaptonuria. J. Inherit. Metab. Dis..

[49] Hartmann P., Seebauer C.T., Schnabl B. (2015). Alcoholic liver disease: The gut microbiome and liver cross talk. Alcohol. Clin. Exp. Res..

[50] Osna N.A., Donohue T.M., Kharbanda K.K. (2017). Alcoholic Liver Disease: Pathogenesis and Current Management. Alcohol. Res..

[51] Lieber C.S., Teschke R., Hasumura Y., Decarli L.M. (1975). Differences in hepatic and metabolic changes after acute and chronic alcohol consumption. Fed. Proc..

[52] You M., Arteel G.E. (2019). Effect of ethanol on lipid metabolism. J. Hepatol..

[53] Lieber C.S., Jones D.P., Decarli L.M. (1965). Effects of Prolonged Ethanol Intake: Production of Fatty Liver Despite Adequate Diets. J. Clin. Investig..

[54] Lieber C.S., DeCarli L.M. (1989). Liquid diet technique of ethanol administration: 1989 Update. Alcohol Alcohol..

[55] Guo F., Zheng K., Benede-Ubieto R., Cubero F.J., Nevzorova Y.A. (2018). The Lieber-DeCarli Diet-A Flagship Model for Experimental Alcoholic Liver Disease. Alcohol Clin. Exp. Res..

[56] Kim S.J., Jung Y.S., Kwon D.Y., Kim Y.C. (2008). Alleviation of acute ethanol-induced liver injury and impaired metabolomics of S-containing substances by betaine supplementation. Biochem. Biophys. Res. Commun..

[57] Zivkovic A.M., Bruce German J., Esfandiari F., Halsted C.H. (2009). Quantitative lipid metabolomic changes in alcoholic micropigs with fatty liver disease. Alcohol Clin. Exp. Res..

[58] Manna S.K., Patterson A.D., Yang Q., Krausz K.W., Li H., Idle J.R., Fornace A.J., Gonzalez F.J. (2010). Identification of noninvasive biomarkers for alcohol-induced liver disease using urinary metabolomics and the Ppara-null mouse. J. Proteome Res..

[59] Manna S.K., Patterson A.D., Yang Q., Krausz K.W., Idle J.R., Fornace A.J., Gonzalez F.J. (2011). UPLC-MS-based urine metabolomics reveals indole-3-lactic acid and phenyllactic acid as conserved biomarkers for alcohol-induced liver disease in the Ppara-null mouse model. J. Proteome Res..

[60] Fernando H., Bhopale K.K., Kondraganti S., Kaphalia B.S., Shakeel Ansari G.A. (2011). Lipidomic changes in rat liver after long-term exposure to ethanol. Toxicol. Appl. Pharmacol..

[61] Suciu A.M., Crisan D.A., Procopet B.D., Radu C.I., Socaciu C., Tantau M.V., Stefanescu H.O., Grigorescu M. (2018). What’s in Metabolomics for Alcoholic Liver Disease?. J. Gastrointestin. Liver Dis..

[62] Shi X., Yao D., Chen C. (2012). Identification of N-acetyltaurine as a novel metabolite of ethanol through metabolomics-guided biochemical analysis. J. Biol. Chem..

[63] Johnson C.H., Patterson A.D., Krausz K.W., Lanz C., Kang D.W., Luecke H., Gonzalez F.J., Idle J.R. (2011). Radiation metabolomics. 4. UPLC-ESI-QTOFMS-Based metabolomics for urinary biomarker discovery in gamma-irradiated rats. Radiat. Res..

[64] Johnson C.H., Patterson A.D., Krausz K.W., Kalinich J.F., Tyburski J.B., Kang D.W., Luecke H., Gonzalez F.J., Blakely W.F., Idle J.R. (2012). Radiation metabolomics. 5. Identification of urinary biomarkers of ionizing radiation exposure in nonhuman primates by mass spectrometry-based metabolomics. Radiat. Res..

[65] Luginbuhl M., Rutjens S., Konig S., Furrer J., Weinmann W. (2016). N-Acetyltaurine as a novel urinary ethanol marker in a drinking study. Anal. Bioanal. Chem..

[66] Luginbuhl M., Konig S., Schurch S., Weinmann W. (2017). Evaluation of N-acetyltaurine as an ethanol marker in human blood. Alcohol.

[67] Xie G., Zhong W., Li H., Li Q., Qiu Y., Zheng X., Chen H., Zhao X., Zhang S., Zhou Z. (2013). Alteration of bile acid metabolism in the rat induced by chronic ethanol consumption. FASEB J..

[68] Xie G., Zhong W., Zheng X., Li Q., Qiu Y., Li H., Chen H., Zhou Z., Jia W. (2013). Chronic ethanol consumption alters mammalian gastrointestinal content metabolites. J. Proteome Res..

[69] Chen P., Torralba M., Tan J., Embree M., Zengler K., Starkel P., van Pijkeren J.P., DePew J., Loomba R., Ho S.B. (2015). Supplementation of saturated long-chain fatty acids maintains intestinal eubiosis and reduces ethanol-induced liver injury in mice. Gastroenterology.

[70] Fang H., Zhang A.H., Sun H., Yu J.B., Wang L., Wang X.J. (2019). High-throughput metabolomics screen coupled with multivariate statistical analysis identifies therapeutic targets in alcoholic liver disease rats using liquid chromatography-mass spectrometry. J. Chromatogr. B Anal. Technol. Biomed. Life Sci..

[71] Zhang T., Zhang A., Qiu S., Sun H., Guan Y., Wang X. (2017). High-throughput metabolomics approach reveals new mechanistic insights for drug response of phenotypes of geniposide towards alcohol-induced liver injury by using liquid chromatography coupled to high resolution mass spectrometry. Mol. Biosyst..

[72] Deda O., Virgiliou C., Orfanidis A., Gika H.G. (2019). Study of Fecal and Urinary Metabolite Perturbations Induced by Chronic Ethanol Treatment in Mice by UHPLC-MS/MS Targeted Profiling. Metabolites.

[73] He L., Li F., Yin X., Bohman P., Kim S., McClain C.J., Feng W., Zhang X. (2019). Profiling of Polar Metabolites in Mouse Feces Using Four Analytical Platforms to Study the Effects Of Cathelicidin-Related Antimicrobial Peptide in Alcoholic Liver Disease. J. Proteome Res..

[74] Gao B., Lang S., Duan Y., Wang Y., Shawcross D.L., Louvet A., Mathurin P., Ho S.B., Starkel P., Schnabl B. (2019). Serum and Fecal Oxylipins in Patients with Alcohol-Related Liver Disease. Dig. Dis. Sci..

[75] Michelena J., Alonso C., Martinez-Arranz I., Altamirano J., Mayo R., Sancho-Bru P., Bataller R., Gines P., Castro A., Caballeria J. (2019). Metabolomics Discloses a New Non-invasive Method for the Diagnosis and Prognosis of Patients with Alcoholic Hepatitis. Ann. Hepatol..

[76] Keitel V., Droge C., Haussinger D. (2019). Targeting FXR in Cholestasis. Handb. Exp. Pharmacol..

[77] Ishihara K., Katsutani N., Asai N., Inomata A., Uemura Y., Suganuma A., Sawada K., Yokoi T., Aoki T. (2009). Identification of urinary biomarkers useful for distinguishing a difference in mechanism of toxicity in rat model of cholestasis. Basic Clin. Pharmacol. Toxicol..

[78] Cho J.Y., Matsubara T., Kang D.W., Ahn S.H., Krausz K.W., Idle J.R., Luecke H., Gonzalez F.J. (2010). Urinary metabolomics in Fxr-null mice reveals activated adaptive metabolic pathways upon bile acid challenge. J. Lipid Res..

[79] Passmore I.J., Letertre M.P.M., Preston M.D., Bianconi I., Harrison M.A., Nasher F., Kaur H., Hong H.A., Baines S.D., Cutting S.M. (2018). Para-cresol production by Clostridium difficile affects microbial diversity and membrane integrity of Gram-negative bacteria. PLoS Pathog.

[80] Matsubara T., Tanaka N., Sato M., Kang D.W., Krausz K.W., Flanders K.C., Ikeda K., Luecke H., Wakefield L.M., Gonzalez F.J. (2012). TGF-beta-SMAD3 signaling mediates hepatic bile acid and phospholipid metabolism following lithocholic acid-induced liver injury. J. Lipid Res..

[81] Aoki M., Konya Y., Takagaki T., Umemura K., Sogame Y., Katsumata T., Komuro S. (2011). Metabolomic investigation of cholestasis in a rat model using ultra-performance liquid chromatography/tandem mass spectrometry. Rapid Commun. Mass Spectrom..

[82] Yamazaki M., Miyake M., Sato H., Masutomi N., Tsutsui N., Adam K.P., Alexander D.C., Lawton K.A., Milburn M.V., Ryals J.A. (2013). Perturbation of bile acid homeostasis is an early pathogenesis event of drug induced liver injury in rats. Toxicol. Appl. Pharmacol..

[83] Chen Z., Zhu Y., Zhao Y., Ma X., Niu M., Wang J., Su H., Wang R., Li J., Liu L. (2016). Serum Metabolomic Profiling in a Rat Model Reveals Protective Function of Paeoniflorin Against ANIT Induced Cholestasis. Phytother. Res..

[84] Ma X., Chi Y.H., Niu M., Zhu Y., Zhao Y.L., Chen Z., Wang J.B., Zhang C.E., Li J.Y., Wang L.F. (2016). Metabolomics Coupled with Multivariate Data and Pathway Analysis on Potential Biomarkers in Cholestasis and Intervention Effect of Paeonia lactiflora Pall. Front. Pharmacol..

[85] Zhang C.E., Niu M., Li R.Y., Feng W.W., Ma X., Dong Q., Ma Z.J., Li G.Q., Meng Y.K., Wang Y. (2016). Untargeted Metabolomics Reveals Dose-Response Characteristics for Effect of Rhubarb in a Rat Model of Cholestasis. Front. Pharmacol..

[86] Sun H., Zhang A.H., Zou D.X., Sun W.J., Wu X.H., Wang X.J. (2014). Metabolomics coupled with pattern recognition and pathway analysis on potential biomarkers in liver injury and hepatoprotective effects of yinchenhao. Appl. Biochem. Biotechnol..

[87] Li Y.F., Wu J.S., Li Y.Y., Dai Y., Zheng M., Zeng J.K., Wang G.F., Wang T.M., Li W.K., Zhang X.Y. (2017). Chicken bile powder protects against alpha-naphthylisothiocyanate-induced cholestatic liver injury in mice. Oncotarget.

[88] Wu J.S., Li Y.F., Li Y.Y., Dai Y., Li W.K., Zheng M., Shi Z.C., Shi R., Wang T.M., Ma B.L. (2017). Huangqi Decoction Alleviates Alpha-Naphthylisothiocyanate Induced Intrahepatic Cholestasis by Reversing Disordered Bile Acid and Glutathione Homeostasis in Mice. Front. Pharmacol..

[89] Han H., Xu L., Xiong K., Zhang T., Wang Z. (2018). Exploration of Hepatoprotective Effect of Gentiopicroside on Alpha-Naphthylisothiocyanate-Induced Cholestatic Liver Injury in Rats by Comprehensive Proteomic and Metabolomic Signatures. Cell Physiol. Biochem..

[90] Zhu G., Feng F. (2019). UPLC-MS-based metabonomic analysis of intervention effects of Da-Huang-Xiao-Shi decoction on ANIT-induced cholestasis. J. Ethnopharmacol..

[91] Ma X., Idle J.R., Krausz K.W., Tan D.X., Ceraulo L., Gonzalez F.J. (2006). Urinary metabolites and antioxidant products of exogenous melatonin in the mouse. J. Pineal Res..

[92] Ma X., Chen C., Krausz K.W., Idle J.R., Gonzalez F.J. (2008). A metabolomic perspective of melatonin metabolism in the mouse. Endocrinology.

[93] Yu H., Li Y., Xu Z., Wang D., Shi S., Deng H., Zeng B., Zheng Z., Sun L., Deng X. (2018). Identification of potential biomarkers in cholestasis and the therapeutic effect of melatonin by metabolomics, multivariate data and pathway analyses. Int J. Mol. Med..

[94] Lin S., Wang T.Y., Xu H.R., Zhang X.N., Wang Q., Liu R., Li Q., Bi K.S. (2019). A systemic combined nontargeted and targeted LC-MS based metabolomic strategy of plasma and liver on pathology exploration of alpha-naphthylisothiocyanate induced cholestatic liver injury in mice. J. Pharm. Biomed. Anal..

[95] Dai M., Hua H., Lin H., Xu G., Hu X., Li F., Gonzalez F.J., Liu A., Yang J. (2018). Targeted Metabolomics Reveals a Protective Role for Basal PPARalpha in Cholestasis Induced by alpha-Naphthylisothiocyanate. J. Proteome Res..

[96] Wang B.L., Zhang C.W., Wang L., Tang K.L., Tanaka N., Gonzalez F.J., Xu Y., Fang Z.Z. (2019). Lipidomics reveal aryl hydrocarbon receptor (Ahr)-regulated lipid metabolic pathway in alpha-naphthyl isothiocyanate (ANIT)-induced intrahepatic cholestasis. Xenobiotica.

[97] Fu K., Wang C., Gao Y., Fan S., Zhang H., Sun J., Jiang Y., Liu C., Guan L., Liu J. (2019). Metabolomics and Lipidomics Reveal the Effect of Hepatic Vps33b Deficiency on Bile Acids and Lipids Metabolism. Front. Pharmacol..

[98] Long Y., Dong X., Yuan Y., Huang J., Song J., Sun Y., Lu Z., Yang L., Yu W. (2015). Metabolomics changes in a rat model of obstructive jaundice: Mapping to metabolism of amino acids, carbohydrates and lipids as well as oxidative stress. J. Clin. Biochem. Nutr..

[99] Wei D.D., Wang J.S., Duan J.A., Kong L.Y. (2018). Metabolomic Assessment of Acute Cholestatic Injuries Induced by Thioacetamide and by Bile Duct Ligation, and the Protective Effects of Huang-Lian-Jie-Du-Decoction. Front. Pharmacol..

[100] Yang R., Zhao Q., Hu D.D., Xiao X.R., Huang J.F., Li F. (2018). Metabolomic analysis of cholestatic liver damage in mice. Food Chem. Toxicol..

[101] Lian J.S., Liu W., Hao S.R., Chen D.Y., Wang Y.Y., Yang J.L., Jia H.Y., Huang J.R. (2015). A serum metabolomic analysis for diagnosis and biomarker discovery of primary biliary cirrhosis and autoimmune hepatitis. Hepatobiliary Pancreat Dis. Int..

[102] Trottier J., Bialek A., Caron P., Straka R.J., Heathcote J., Milkiewicz P., Barbier O. (2012). Metabolomic profiling of 17 bile acids in serum from patients with primary biliary cirrhosis and primary sclerosing cholangitis: A pilot study. Dig. Liver Dis..

[103] Bell L.N., Wulff J., Comerford M., Vuppalanchi R., Chalasani N. (2015). Serum metabolic signatures of primary biliary cirrhosis and primary sclerosing cholangitis. Liver Int..

[104] Tang Y.M., Wang J.P., Bao W.M., Yang J.H., Ma L.K., Yang J., Chen H., Xu Y., Yang L.H., Li W. (2015). Urine and serum metabolomic profiling reveals that bile acids and carnitine may be potential biomarkers of primary biliary cirrhosis. Int. J. Mol. Med..

[105] Vignoli A., Orlandini B., Tenori L., Biagini M.R., Milani S., Renzi D., Luchinat C., Calabro A.S. (2019). Metabolic Signature of Primary Biliary Cholangitis and Its Comparison with Celiac Disease. J. Proteome Res..

[106] Ozkan S., Ceylan Y., Ozkan O.V., Yildirim S. (2015). Review of a challenging clinical issue: Intrahepatic cholestasis of pregnancy. World J. Gastroenterol..

[107] Ma L., Zhang X., Pan F., Cui Y., Yang T., Deng L., Shao Y., Ding M. (2017). Urinary metabolomic analysis of intrahepatic cholestasis of pregnancy based on high performance liquid chromatography/mass spectrometry. Clin. Chim. Acta..

[108] Cui Y., Xu B., Zhang X., He Y., Shao Y., Ding M. (2018). Diagnostic and therapeutic profiles of serum bile acids in women with intrahepatic cholestasis of pregnancy-a pseudo-targeted metabolomics study. Clin. Chim. Acta..

[109] Wang P., Zhong H., Song Y., Yuan P., Li Y., Lin S., Zhang X., Li J., Che L., Feng B. (2019). Targeted metabolomics analysis of maternal-placental-fetal metabolism in pregnant swine reveals links in fetal bile acid homeostasis and sulfation capacity. Am. J. Physiol. Gastrointest. Liver Physiol..

[110] Chen X., Zhang X., Xu B., Cui Y., He Y., Yang T., Shao Y., Ding M. (2019). The urinary bile acid profiling analysis of asymptomatic hypercholanemia of pregnancy: A pseudo-targeted metabolomics study. Clin. Chim. Acta.

[111] De Seymour J.V., Tu S., He X., Zhang H., Han T.L., Baker P.N., Sulek K. (2018). Metabolomic profiling of maternal hair suggests rapid development of intrahepatic cholestasis of pregnancy. Metabolomics.

[112] Li Y., Zhang X., Chen J., Feng C., He Y., Shao Y., Ding M. (2018). Targeted metabolomics of sulfated bile acids in urine for the diagnosis and grading of intrahepatic cholestasis of pregnancy. Genes Dis..

[113] Li W.W., Yang Y., Dai Q.G., Lin L.L., Xie T., He L.L., Tao J.L., Shan J.J., Wang S.C. (2018). Non-invasive urinary metabolomic profiles discriminate biliary atresia from infantile hepatitis syndrome. Metabolomics.

[114] Bataller R., Brenner D.A. (2005). Liver fibrosis. J. Clin. Investig..

[115] Goodman Z.D. (2007). Grading and staging systems for inflammation and fibrosis in chronic liver diseases. J. Hepatol..

[116] D’Amico G., Garcia-Tsao G., Pagliaro L. (2006). Natural history and prognostic indicators of survival in cirrhosis: A systematic review of 118 studies. J. Hepatol..

[117] Fleming K.M., Aithal G.P., Card T.R., West J. (2012). All-cause mortality in people with cirrhosis compared with the general population: A population-based cohort study. Liver Int..

[118] Chang M.L., Yang S.S. (2019). Metabolic Signature of Hepatic Fibrosis: From Individual Pathways to Systems Biology. Cells.

[119] Tokushige K., Hashimoto E., Kodama K., Tobari M., Matsushita N., Kogiso T., Taniai M., Torii N., Shiratori K., Nishizaki Y. (2013). Serum metabolomic profile and potential biomarkers for severity of fibrosis in nonalcoholic fatty liver disease. J. Gastroenterol..

[120] Fishman J., Schneider J., Hershcope R.J., Bradlow H.L. (1984). Increased estrogen-16 alpha-hydroxylase activity in women with breast and endometrial cancer. J. Steroid Biochem..

[121] Batista A.D., Barros C.J.P., Costa T., de Godoy M.M.G., Silva R.D., Santos J.C., de Melo Lira M.M., Juca N.T., Lopes E.P.A., Silva R.O. (2018). Proton nuclear magnetic resonance-based metabonomic models for non-invasive diagnosis of liver fibrosis in chronic hepatitis C: Optimizing the classification of intermediate fibrosis. World J. Hepatol..

[122] Abellona U.M.R., Taylor-Robinson S.D. (2018). Comments on Gabbani, et al. Metabolomic analysis with (1)H NMR for non-invasive diagnosis of hepatic fibrosis degree in patients with chronic hepatitis C. Dig. Liver Dis..

[123] Wu F., Zheng H., Yang Z.T., Cheng B., Wu J.X., Liu X.W., Tang C.L., Lu S.Y., Chen Z.N., Song F.M. (2017). Urinary metabonomics study of the hepatoprotective effects of total alkaloids from Corydalis saxicola Bunting on carbon tetrachloride-induced chronic hepatotoxicity in rats using (1)H NMR analysis. J. Pharm. Biomed. Anal..

[124] Liu X.W., Tang C.L., Zheng H., Wu J.X., Wu F., Mo Y.Y., Liu X., Zhu H.J., Yin C.L., Cheng B. (2018). Investigation of the hepatoprotective effect of Corydalis saxicola Bunting on carbon tetrachloride-induced liver fibrosis in rats by (1)H-NMR-based metabonomics and network pharmacology approaches. J. Pharm. Biomed. Anal..

[125] Feng X., Li M.H., Xia J., Deng Ba D.J., Ruan L.Y., Xing Y.X., Chen C., Wang J.S., Zhong G.J. (2018). Tibetan Medical Formula Shi-Wei-Gan-Ning-Pill Protects Against Carbon Tetrachloride-Induced Liver Fibrosis—An NMR-Based Metabolic Profiling. Front. Pharmacol..

[126] Li M.H., Feng X., Deng Ba D.J., Chen C., Ruan L.Y., Xing Y.X., Chen L.Y., Zhong G.J., Wang J.S. (2019). Hepatoprotection of Herpetospermum caudigerum Wall. against CCl4-induced liver fibrosis on rats. J. Ethnopharmacol..

[127] Yin P., Wan D., Zhao C., Chen J., Zhao X., Wang W., Lu X., Yang S., Gu J., Xu G. (2009). A metabonomic study of hepatitis B-induced liver cirrhosis and hepatocellular carcinoma by using RP-LC and HILIC coupled with mass spectrometry. Mol. Biosyst..

[128] Xue R., Dong L., Wu H., Liu T., Wang J., Shen X. (2009). Gas chromatography/mass spectrometry screening of serum metabolomic biomarkers in hepatitis B virus infected cirrhosis patients. Clin. Chem. Lab. Med..

[129] Waldhier M.C., Almstetter M.F., Nurnberger N., Gruber M.A., Dettmer K., Oefner P.J. (2011). Improved enantiomer resolution and quantification of free D-amino acids in serum and urine by comprehensive two-dimensional gas chromatography-time-of-flight mass spectrometry. J. Chromatogr. A.

[130] Bastings J., van Eijk H.M., Olde Damink S.W., Rensen S.S. (2019). d-amino Acids in Health and Disease: A Focus on Cancer. Nutrients.

[131] Huang H.J., Zhang A.Y., Cao H.C., Lu H.F., Wang B.H., Xie Q., Xu W., Li L.J. (2013). Metabolomic analyses of faeces reveals malabsorption in cirrhotic patients. Dig. Liver Dis..

[132] Liu Z., Zhang Z., Huang M., Sun X., Liu B., Guo Q., Chang Q., Duan Z. (2018). Taurocholic acid is an active promoting factor, not just a biomarker of progression of liver cirrhosis: Evidence from a human metabolomic study and in vitro experiments. BMC Gastroenterol..

[133] Patterson A.D., Maurhofer O., Beyoglu D., Lanz C., Krausz K.W., Pabst T., Gonzalez F.J., Dufour J.F., Idle J.R. (2011). Aberrant lipid metabolism in hepatocellular carcinoma revealed by plasma metabolomics and lipid profiling. Cancer Res..

[134] Osman D., Ali O., Obada M., El-Mezayen H., El-Said H. (2017). Chromatographic determination of some biomarkers of liver cirrhosis and hepatocellular carcinoma in Egyptian patients. Biomed. Chromatogr..

[135] Wang B., Chen D., Chen Y., Hu Z., Cao M., Xie Q., Chen Y., Xu J., Zheng S., Li L. (2012). Metabonomic profiles discriminate hepatocellular carcinoma from liver cirrhosis by ultraperformance liquid chromatography-mass spectrometry. J. Proteome Res..

[136] Koller A., Aldwin L., Natelson S. (1975). Hepatic synthesis of canavaninosuccinate from ureidohomoserine and aspartate, and its conversion to guanidinosuccinate. Clin. Chem..

[137] Chen D.Q., Cao G., Chen H., Liu D., Su W., Yu X.Y., Vaziri N.D., Liu X.H., Bai X., Zhang L. (2017). Gene and protein expressions and metabolomics exhibit activated redox signaling and wnt/beta-catenin pathway are associated with metabolite dysfunction in patients with chronic kidney disease. Redox Biol..

[138] Mindikoglu A.L., Opekun A.R., Putluri N., Devaraj S., Sheikh-Hamad D., Vierling J.M., Goss J.A., Rana A., Sood G.K., Jalal P.K. (2018). Unique metabolomic signature associated with hepatorenal dysfunction and mortality in cirrhosis. Transl. Res..

[139] Liu Y., Hong Z., Tan G., Dong X., Yang G., Zhao L., Chen X., Zhu Z., Lou Z., Qian B. (2014). NMR and LC/MS-based global metabolomics to identify serum biomarkers differentiating hepatocellular carcinoma from liver cirrhosis. Int J. Cancer.

[140] McPhail M.J.W., Shawcross D.L., Lewis M.R., Coltart I., Want E.J., Antoniades C.G., Veselkov K., Triantafyllou E., Patel V., Pop O. (2016). Multivariate metabotyping of plasma predicts survival in patients with decompensated cirrhosis. J. Hepatol..

[141] Dai W., Yin P., Chen P., Kong H., Luo P., Xu Z., Lu X., Xu G. (2014). Study of urinary steroid hormone disorders: Difference between hepatocellular carcinoma in early stage and cirrhosis. Anal. Bioanal. Chem..

[142] Gao R., Cheng J., Fan C., Shi X., Cao Y., Sun B., Ding H., Hu C., Dong F., Yan X. (2015). Serum Metabolomics to Identify the Liver Disease-Specific Biomarkers for the Progression of Hepatitis to Hepatocellular Carcinoma. Sci. Rep..

[143] Hou Q., Duan Z.J. (2016). Metabonomic window into hepatitis B virus-related hepatic diseases. World J. Hepatol..

[144] Lu Y., Fang J., Zou L., Cui L., Liang X., Lim S.G., Dan Y.Y., Ong C.N. (2018). Omega-6-derived oxylipin changes in serum of patients with hepatitis B virus-related liver diseases. Metabolomics.

[145] Cabral M., Martin-Venegas R., Moreno J.J. (2014). Differential cell growth/apoptosis behavior of 13-hydroxyoctadecadienoic acid enantiomers in a colorectal cancer cell line. Am. J. Physiol. Gastrointest. Liver Physiol..

[146] Zhao C.Q., Chen L., Cai H., Yao W.L., Zhou Q., Zhu H.M., Gao Y., Liu P., Gou X.J., Zhang H. (2018). Classification of Gan Dan Shi Re Pattern and Gan Shen Yin Xu Pattern in Patients with Hepatitis B Cirrhosis Using Metabonomics. Evid. Based Complement. Altern. Med..

[147] Zhou K., Xie G., Wen J., Wang J., Pan W., Zhou Y., Xiao Y., Wang Y., Jia W., Cai W. (2016). Histamine is correlated with liver fibrosis in biliary atresia. Dig. Liver Dis..

[148] Jimenez B., Montoliu C., MacIntyre D.A., Serra M.A., Wassel A., Jover M., Romero-Gomez M., Rodrigo J.M., Pineda-Lucena A., Felipo V. (2010). Serum metabolic signature of minimal hepatic encephalopathy by (1)H-nuclear magnetic resonance. J. Proteome Res..

[149] Martinez-Granados B., Morales J.M., Rodrigo J.M., Del Olmo J., Serra M.A., Ferrandez A., Celda B., Monleon D. (2011). Metabolic profile of chronic liver disease by NMR spectroscopy of human biopsies. Int. J. Mol. Med..

[150] Schofield Z., Reed M.A., Newsome P.N., Adams D.H., Gunther U.L., Lalor P.F. (2017). Changes in human hepatic metabolism in steatosis and cirrhosis. World J. Gastroenterol..

[151] Gao H., Lu Q., Liu X., Cong H., Zhao L., Wang H., Lin D. (2009). Application of 1H NMR-based metabonomics in the study of metabolic profiling of human hepatocellular carcinoma and liver cirrhosis. Cancer Sci..

[152] Amathieu R., Nahon P., Triba M., Bouchemal N., Trinchet J.C., Beaugrand M., Dhonneur G., Le Moyec L. (2011). Metabolomic approach by 1H NMR spectroscopy of serum for the assessment of chronic liver failure in patients with cirrhosis. J. Proteome Res..

[153] Qi S.W., Tu Z.G., Peng W.J., Wang L.X., Ou-Yang X., Cai A.J., Dai Y. (2012). (1)H NMR-based serum metabolic profiling in compensated and decompensated cirrhosis. World J. Gastroenterol..

[154] Corbin I.R., Ryner L.N., Singh H., Minuk G.Y. (2004). Quantitative hepatic phosphorus-31 magnetic resonance spectroscopy in compensated and decompensated cirrhosis. Am. J. Physiol. Gastrointest. Liver Physiol..

[155] Traussnigg S., Kienbacher C., Gajdosik M., Valkovic L., Halilbasic E., Stift J., Rechling C., Hofer H., Steindl-Munda P., Ferenci P. (2017). Ultra-high-field magnetic resonance spectroscopy in non-alcoholic fatty liver disease: Novel mechanistic and diagnostic insights of energy metabolism in non-alcoholic steatohepatitis and advanced fibrosis. Liver Int..

[156] Iebba V., Guerrieri F., Di Gregorio V., Levrero M., Gagliardi A., Santangelo F., Sobolev A.P., Circi S., Giannelli V., Mannina L. (2018). Combining amplicon sequencing and metabolomics in cirrhotic patients highlights distinctive microbiota features involved in bacterial translocation, systemic inflammation and hepatic encephalopathy. Sci. Rep..

[157] Liu Y., Li J., Jin Y., Zhao L., Zhao F., Feng J., Li A., Wei Y. (2018). Splenectomy Leads to Amelioration of Altered Gut Microbiota and Metabolome in Liver Cirrhosis Patients. Front. Microbiol..

[158] Shao L., Ling Z., Chen D., Liu Y., Yang F., Li L. (2018). Disorganized Gut Microbiome Contributed to Liver Cirrhosis Progression: A Meta-Omics-Based Study. Front. Microbiol..

[159] Cox I.J., Idilman R., Fagan A., Turan D., Ajayi L., Le Guennec A.D., Taylor-Robinson S.D., Karakaya F., Gavis E., Andrew Atkinson R. (2019). Metabolomics and microbial composition increase insight into the impact of dietary differences in cirrhosis. Liver Int..

[160] Amathieu R., Triba M.N., Nahon P., Bouchemal N., Kamoun W., Haouache H., Trinchet J.C., Savarin P., Le Moyec L., Dhonneur G. (2014). Serum 1H-NMR metabolomic fingerprints of acute-on-chronic liver failure in intensive care unit patients with alcoholic cirrhosis. PLoS ONE.

[161] Dabos K.J., Parkinson J.A., Sadler I.H., Plevris J.N., Hayes P.C. (2015). (1)H nuclear magnetic resonance spectroscopy-based metabonomic study in patients with cirrhosis and hepatic encephalopathy. World J. Hepatol..

[162] Weiss N., Barbier Saint Hilaire P., Colsch B., Isnard F., Attala S., Schaefer A., Amador M.D., Rudler M., Lamari F., Sedel F. (2016). Cerebrospinal fluid metabolomics highlights dysregulation of energy metabolism in overt hepatic encephalopathy. J. Hepatol..

[163] Embade N., Marino Z., Diercks T., Cano A., Lens S., Cabrera D., Navasa M., Falcon-Perez J.M., Caballeria J., Castro A. (2016). Metabolic Characterization of Advanced Liver Fibrosis in HCV Patients as Studied by Serum 1H-NMR Spectroscopy. PLoS ONE.

[164] Gabbani T., Marsico M., Bernini P., Lorefice E., Grappone C., Biagini M.R., Milani S., Annese V. (2017). Metabolomic analysis with (1)H-NMR for non-invasive diagnosis of hepatic fibrosis degree in patients with chronic hepatitis C. Dig. Liver Dis..

[165] Wei D.D., Wang J.S., Wang P.R., Li M.H., Yang M.H., Kong L.Y. (2014). Toxic effects of chronic low-dose exposure of thioacetamide on rats based on NMR metabolic profiling. J. Pharm. Biomed. Anal..

[166] Zhang H., Wang X., Hu P., Zhou W., Zhang M., Liu J., Wang Y., Liu P., Luo G. (2016). Serum Metabolomic Characterization of Liver Fibrosis in Rats and Anti-Fibrotic Effects of Yin-Chen-Hao-Tang. Molecules.

[167] Song Y.N., Zhang G.B., Lu Y.Y., Chen Q.L., Yang L., Wang Z.T., Liu P., Su S.B. (2016). Huangqi decoction alleviates dimethylnitrosamine-induced liver fibrosis: An analysis of bile acids metabolic mechanism. J. Ethnopharmacol..

[168] Yu J., He J.Q., Chen D.Y., Pan Q.L., Yang J.F., Cao H.C., Li L.J. (2019). Dynamic changes of key metabolites during liver fibrosis in rats. World J. Gastroenterol..

[169] Zhang K., Zhang Y., Li N., Xing F., Zhao J., Yang T., Liu C., Feng N. (2019). An herbal-compound-based combination therapy that relieves cirrhotic ascites by affecting the L-arginine/nitric oxide pathway: A metabolomics-based systematic study. J. Ethnopharmacol..

[170] Wang G., Li Z., Li H., Li L., Li J., Yu C. (2016). Metabolic Profile Changes of CCl(4)-Liver Fibrosis and Inhibitory Effects of Jiaqi Ganxian Granule. Molecules.

[171] Chang H., Meng H.Y., Wang Y., Teng Z., Liu S.M. (2018). [Inhibitory effect of Scutellariae Radix on hepatic fibrosis based on urinary metabonomic]. Zhongguo Zhong Yao Za Zhi.

[172] Zheng M., Li Y.Y., Wang G.F., Jin J.Y., Wang Y.H., Wang T.M., Yang L., Liu S.Y., Wu J.S., Wang Z.T. (2019). Protective effect of cultured bear bile powder against dimethylnitrosamine-induced hepatic fibrosis in rats. Biomed. Pharmacother..

[173] EASL, EASD, EASO (2016). EASL-EASD-EASO Clinical Practice Guidelines for the management of non-alcoholic fatty liver disease. J. Hepatol..

[174] Puri P., Daita K., Joyce A., Mirshahi F., Santhekadur P.K., Cazanave S., Luketic V.A., Siddiqui M.S., Boyett S., Min H.K. (2018). The presence and severity of nonalcoholic steatohepatitis is associated with specific changes in circulating bile acids. Hepatology.

[175] Fedchuk L., Nascimbeni F., Pais R., Charlotte F., Housset C., Ratziu V., Group L.S. (2014). Performance and limitations of steatosis biomarkers in patients with nonalcoholic fatty liver disease. Aliment. Pharmacol. Ther..

[176] Mato J.M., Alonso C., Noureddin M., Lu S.C. (2019). Biomarkers and subtypes of deranged lipid metabolism in non-alcoholic fatty liver disease. World J. Gastroenterol..

[177] Johnson C.H., Ivanisevic J., Siuzdak G. (2016). Metabolomics: Beyond biomarkers and towards mechanisms. Nat. Rev. Mol. Cell Biol..

[178] Puri P., Wiest M.M., Cheung O., Mirshahi F., Sargeant C., Min H.K., Contos M.J., Sterling R.K., Fuchs M., Zhou H. (2009). The plasma lipidomic signature of nonalcoholic steatohepatitis. Hepatology.

[179] Barr J., Vazquez-Chantada M., Alonso C., Perez-Cormenzana M., Mayo R., Galan A., Caballeria J., Martin-Duce A., Tran A., Wagner C. (2010). Liquid chromatography-mass spectrometry-based parallel metabolic profiling of human and mouse model serum reveals putative biomarkers associated with the progression of nonalcoholic fatty liver disease. J. Proteome Res..

[180] Kalhan S.C., Guo L., Edmison J., Dasarathy S., McCullough A.J., Hanson R.W., Milburn M. (2011). Plasma metabolomic profile in nonalcoholic fatty liver disease. Metabolism.

[181] Li H., Wang L., Yan X., Liu Q., Yu C., Wei H., Li Y., Zhang X., He F., Jiang Y. (2011). A proton nuclear magnetic resonance metabonomics approach for biomarker discovery in nonalcoholic fatty liver disease. J. Proteome Res..

[182] Lehmann R., Franken H., Dammeier S., Rosenbaum L., Kantartzis K., Peter A., Zell A., Adam P., Li J., Xu G. (2013). Circulating lysophosphatidylcholines are markers of a metabolically benign nonalcoholic fatty liver. Diabetes Care.

[183] Ferslew B.C., Xie G., Johnston C.K., Su M., Stewart P.W., Jia W., Brouwer K.L., Barritt A.S.t. (2015). Altered Bile Acid Metabolome in Patients with Nonalcoholic Steatohepatitis. Dig. Dis. Sci..

[184] Lake A.D., Novak P., Shipkova P., Aranibar N., Robertson D.G., Reily M.D., Lehman-McKeeman L.D., Vaillancourt R.R., Cherrington N.J. (2015). Branched chain amino acid metabolism profiles in progressive human nonalcoholic fatty liver disease. Amino. Acids.

[185] Loomba R., Quehenberger O., Armando A., Dennis E.A. (2015). Polyunsaturated fatty acid metabolites as novel lipidomic biomarkers for noninvasive diagnosis of nonalcoholic steatohepatitis. J. Lipid Res..

[186] Mannisto V.T., Simonen M., Hyysalo J., Soininen P., Kangas A.J., Kaminska D., Matte A.K., Venesmaa S., Kakela P., Karja V. (2015). Ketone body production is differentially altered in steatosis and non-alcoholic steatohepatitis in obese humans. Liver Int..

[187] Rodriguez-Gallego E., Guirro M., Riera-Borrull M., Hernandez-Aguilera A., Marine-Casado R., Fernandez-Arroyo S., Beltran-Debon R., Sabench F., Hernandez M., del Castillo D. (2015). Mapping of the circulating metabolome reveals alpha-ketoglutarate as a predictor of morbid obesity-associated non-alcoholic fatty liver disease. Int. J. Obes..

[188] Zhou Y., Oresic M., Leivonen M., Gopalacharyulu P., Hyysalo J., Arola J., Verrijken A., Francque S., Van Gaal L., Hyotylainen T. (2016). Noninvasive Detection of Nonalcoholic Steatohepatitis Using Clinical Markers and Circulating Levels of Lipids and Metabolites. Clin. Gastroenterol. Hepatol..

[189] Chiappini F., Coilly A., Kadar H., Gual P., Tran A., Desterke C., Samuel D., Duclos-Vallee J.C., Touboul D., Bertrand-Michel J. (2017). Metabolism dysregulation induces a specific lipid signature of nonalcoholic steatohepatitis in patients. Sci. Rep..

[190] Dong S., Zhan Z.Y., Cao H.Y., Wu C., Bian Y.Q., Li J.Y., Cheng G.H., Liu P., Sun M.Y. (2017). Urinary metabolomics analysis identifies key biomarkers of different stages of nonalcoholic fatty liver disease. World J. Gastroenterol..

[191] O’Sullivan J.F., Morningstar J.E., Yang Q., Zheng B., Gao Y., Jeanfavre S., Scott J., Fernandez C., Zheng H., O’Connor S. (2017). Dimethylguanidino valeric acid is a marker of liver fat and predicts diabetes. J. Clin. Investig..

[192] Qi S., Xu D., Li Q., Xie N., Xia J., Huo Q., Li P., Chen Q., Huang S. (2017). Metabonomics screening of serum identifies pyroglutamate as a diagnostic biomarker for nonalcoholic steatohepatitis. Clin. Chim. Acta.

[193] Keogh A., Senkardes S., Idle J.R., Kucukguzel S.G., Beyoglu D. (2017). A Novel Anti-Hepatitis C Virus and Antiproliferative Agent Alters Metabolic Networks in HepG2 and Hep3B Cells. Metabolites.

[194] Golla S., Golla J.P., Krausz K.W., Manna S.K., Simillion C., Beyoglu D., Idle J.R., Gonzalez F.J. (2017). Metabolomic Analysis of Mice Exposed to Gamma Radiation Reveals a Systemic Understanding of Total-Body Exposure. Radiat Res..

[195] Wang M., Keogh A., Treves S., Idle J.R., Beyoglu D. (2016). The metabolomic profile of gamma-irradiated human hepatoma and muscle cells reveals metabolic changes consistent with the Warburg effect. PeerJ.

[196] Yang R.X., Hu C.X., Sun W.L., Pan Q., Shen F., Yang Z., Su Q., Xu G.W., Fan J.G. (2017). Serum Monounsaturated Triacylglycerol Predicts Steatohepatitis in Patients with Non-alcoholic Fatty Liver Disease and Chronic Hepatitis B. Sci. Rep..

[197] Hoyles L., Fernandez-Real J.M., Federici M., Serino M., Abbott J., Charpentier J., Heymes C., Luque J.L., Anthony E., Barton R.H. (2018). Molecular phenomics and metagenomics of hepatic steatosis in non-diabetic obese women. Nat. Med..

[198] Mayo R., Crespo J., Martinez-Arranz I., Banales J.M., Arias M., Minchole I., Aller de la Fuente R., Jimenez-Aguero R., Alonso C., de Luis D.A. (2018). Metabolomic-based noninvasive serum test to diagnose nonalcoholic steatohepatitis: Results from discovery and validation cohorts. Hepatol. Commun..

[199] Perakakis N., Polyzos S.A., Yazdani A., Sala-Vila A., Kountouras J., Anastasilakis A.D., Mantzoros C.S. (2019). Non-invasive diagnosis of non-alcoholic steatohepatitis and fibrosis with the use of omics and supervised learning: A proof of concept study. Metabolism.

[200] Pietzner M., Budde K., Homuth G., Kastenmuller G., Henning A.K., Artati A., Krumsiek J., Volzke H., Adamski J., Lerch M.M. (2018). Hepatic Steatosis Is Associated With Adverse Molecular Signatures in Subjects Without Diabetes. J. Clin. Endocrinol. Metab..

[201] Romero-Ibarguengoitia M.E., Vadillo-Ortega F., Caballero A.E., Ibarra-Gonzalez I., Herrera-Rosas A., Serratos-Canales M.F., Leon-Hernandez M., Gonzalez-Chavez A., Mummidi S., Duggirala R. (2018). Family history and obesity in youth, their effect on acylcarnitine/aminoacids metabolomics and non-alcoholic fatty liver disease (NAFLD). Structural equation modeling approach. PLoS ONE.

[202] Boone S., Mook-Kanamori D., Rosendaal F., den Heijer M., Lamb H., de Roos A., le Cessie S., Willems van Dijk K., de Mutsert R. (2019). Metabolomics: A search for biomarkers of visceral fat and liver fat content. Metabolomics.

[203] Tan Y., Liu X., Zhou K., He X., Lu C., He B., Niu X., Xiao C., Xu G., Bian Z. (2016). The Potential Biomarkers to Identify the Development of Steatosis in Hyperuricemia. PLoS ONE.

[204] Von Schonfels W., Patsenker E., Fahrner R., Itzel T., Hinrichsen H., Brosch M., Erhart W., Gruodyte A., Vollnberg B., Richter K. (2015). Metabolomic tissue signature in human non-alcoholic fatty liver disease identifies protective candidate metabolites. Liver Int..

[205] Alonso C., Fernandez-Ramos D., Varela-Rey M., Martinez-Arranz I., Navasa N., Van Liempd S.M., Lavin Trueba J.L., Mayo R., Ilisso C.P., de Juan V.G. (2017). Metabolomic Identification of Subtypes of Nonalcoholic Steatohepatitis. Gastroenterology.

[206] Chen Y., Li C., Liu L., Guo F., Li S., Huang L., Sun C., Feng R. (2016). Serum metabonomics of NAFLD plus T2DM based on liquid chromatography-mass spectrometry. Clin. Biochem..

[207] Semmo N., Weber T., Idle J.R., Beyoglu D. (2015). Metabolomics reveals that aldose reductase activity due to AKR1B10 is upregulated in hepatitis C virus infection. J. Viral Hepat..

[208] Liu C.R., Li X., Chan P.L., Zhuang H., Jia J.D., Wang X., Lo Y.R., Walsh N. (2019). Prevalence of hepatitis C virus infection among key populations in China: A systematic review. Int. J. Infect. Dis..

[209] Alkhouri N., Cikach F., Eng K., Moses J., Patel N., Yan C., Hanouneh I., Grove D., Lopez R., Dweik R. (2014). Analysis of breath volatile organic compounds as a noninvasive tool to diagnose nonalcoholic fatty liver disease in children. Eur. J. Gastroenterol. Hepatol..

[210] Reid D.T., McDonald B., Khalid T., Vo T., Schenck L.P., Surette M.G., Beck P.L., Reimer R.A., Probert C.S., Rioux K.P. (2016). Unique microbial-derived volatile organic compounds in portal venous circulation in murine non-alcoholic fatty liver disease. Biochim. Biophys. Acta.

[211] Pastore A., Alisi A., di Giovamberardino G., Crudele A., Ceccarelli S., Panera N., Dionisi-Vici C., Nobili V. (2014). Plasma levels of homocysteine and cysteine increased in pediatric NAFLD and strongly correlated with severity of liver damage. Int. J. Mol. Sci..

[212] Jin R., Banton S., Tran V.T., Konomi J.V., Li S., Jones D.P., Vos M.B. (2016). Amino Acid Metabolism is Altered in Adolescents with Nonalcoholic Fatty Liver Disease-An Untargeted, High Resolution Metabolomics Study. J. Pediatr..

[213] Troisi J., Pierri L., Landolfi A., Marciano F., Bisogno A., Belmonte F., Palladino C., Guercio Nuzio S., Campiglia P., Vajro P. (2017). Urinary Metabolomics in Pediatric Obesity and NAFLD Identifies Metabolic Pathways/Metabolites Related to Dietary Habits and Gut-Liver Axis Perturbations. Nutrients.

[214] Goffredo M., Santoro N., Trico D., Giannini C., D’Adamo E., Zhao H., Peng G., Yu X., Lam T.T., Pierpont B. (2017). A Branched-Chain Amino Acid-Related Metabolic Signature Characterizes Obese Adolescents with Non-Alcoholic Fatty Liver Disease. Nutrients.

[215] Troisi J., Belmonte F., Bisogno A., Pierri L., Colucci A., Scala G., Cavallo P., Mandato C., Di Nuzzi A., Di Michele L. (2019). Metabolomic Salivary Signature of Pediatric Obesity Related Liver Disease and Metabolic Syndrome. Nutrients.

[216] Hartley A., Santos Ferreira D.L., Anderson E.L., Lawlor D.A. (2018). Metabolic profiling of adolescent non-alcoholic fatty liver disease. Wellcome Open Res..

[217] Khusial R.D., Cioffi C.E., Caltharp S.A., Krasinskas A.M., Alazraki A., Knight-Scott J., Cleeton R., Castillo-Leon E., Jones D.P., Pierpont B. (2019). Development of a Plasma Screening Panel for Pediatric Nonalcoholic Fatty Liver Disease Using Metabolomics. Hepatol. Commun..

[218] Tanaka N., Matsubara T., Krausz K.W., Patterson A.D., Gonzalez F.J. (2012). Disruption of phospholipid and bile acid homeostasis in mice with nonalcoholic steatohepatitis. Hepatology.

[219] Tanaka N., Takahashi S., Fang Z.Z., Matsubara T., Krausz K.W., Qu A., Gonzalez F.J. (2014). Role of white adipose lipolysis in the development of NASH induced by methionine- and choline-deficient diet. Biochim. Biophys. Acta.

[220] Thomas A., Stevens A.P., Klein M.S., Hellerbrand C., Dettmer K., Gronwald W., Oefner P.J., Reinders J. (2012). Early changes in the liver-soluble proteome from mice fed a nonalcoholic steatohepatitis inducing diet. Proteomics.

[221] Depner C.M., Traber M.G., Bobe G., Kensicki E., Bohren K.M., Milne G., Jump D.B. (2013). A metabolomic analysis of omega-3 fatty acid-mediated attenuation of western diet-induced nonalcoholic steatohepatitis in LDLR-/- mice. PLoS ONE.

[222] Lake A.D., Novak P., Shipkova P., Aranibar N., Robertson D., Reily M.D., Lu Z., Lehman-McKeeman L.D., Cherrington N.J. (2013). Decreased hepatotoxic bile acid composition and altered synthesis in progressive human nonalcoholic fatty liver disease. Toxicol. Appl. Pharmacol..

[223] Chao J., Huo T.I., Cheng H.Y., Tsai J.C., Liao J.W., Lee M.S., Qin X.M., Hsieh M.T., Pao L.H., Peng W.H. (2014). Gallic acid ameliorated impaired glucose and lipid homeostasis in high fat diet-induced NAFLD mice. PLoS ONE.

[224] Lai Y.S., Chen W.C., Kuo T.C., Ho C.T., Kuo C.H., Tseng Y.J., Lu K.H., Lin S.H., Panyod S., Sheen L.Y. (2015). Mass-Spectrometry-Based Serum Metabolomics of a C57BL/6J Mouse Model of High-Fat-Diet-Induced Non-alcoholic Fatty Liver Disease Development. J. Agric. Food Chem..

[225] Tu L.N., Showalter M.R., Cajka T., Fan S., Pillai V.V., Fiehn O., Selvaraj V. (2017). Metabolomic characteristics of cholesterol-induced non-obese nonalcoholic fatty liver disease in mice. Sci. Rep..

[226] Chen C.J., Liao W.L., Chang C.T., Lin Y.N., Tsai F.J. (2018). Identification of Urinary Metabolite Biomarkers of Type 2 Diabetes Nephropathy Using an Untargeted Metabolomic Approach. J. Proteome Res..

[227] Pandey V., Sultan M., Kashofer K., Ralser M., Amstislavskiy V., Starmann J., Osprian I., Grimm C., Hache H., Yaspo M.L. (2014). Comparative analysis and modeling of the severity of steatohepatitis in DDC-treated mouse strains. PLoS ONE.

[228] Sanyal A.J., Pacana T. (2015). A Lipidomic Readout of Disease Progression in A Diet-Induced Mouse Model of Nonalcoholic Fatty Liver Disease. Trans. Am. Clin. Climatol. Assoc..

[229] Patterson R.E., Kalavalapalli S., Williams C.M., Nautiyal M., Mathew J.T., Martinez J., Reinhard M.K., McDougall D.J., Rocca J.R., Yost R.A. (2016). Lipotoxicity in steatohepatitis occurs despite an increase in tricarboxylic acid cycle activity. Am. J. Physiol. Endocrinol. Metab..

[230] Qian M., Hu H., Zhao D., Wang S., Pan C., Duan X., Gao Y., Liu J., Zhang Y., Yang S. (2019). Coordinated changes of gut microbiome and lipidome differentiates nonalcoholic steatohepatitis (NASH) from isolated steatosis. Liver Int..

[231] Kim K.E., Jung Y., Min S., Nam M., Heo R.W., Jeon B.T., Song D.H., Yi C.O., Jeong E.A., Kim H. (2016). Caloric restriction of db/db mice reverts hepatic steatosis and body weight with divergent hepatic metabolism. Sci. Rep..

[232] Gogiashvili M., Edlund K., Gianmoena K., Marchan R., Brik A., Andersson J.T., Lambert J., Madjar K., Hellwig B., Rahnenfuhrer J. (2017). Metabolic profiling of ob/ob mouse fatty liver using HR-MAS (1)H-NMR combined with gene expression analysis reveals alterations in betaine metabolism and the transsulfuration pathway. Anal. Bioanal. Chem..

[233] Li J., Liu Z., Guo M., Xu K., Jiang M., Lu A., Gao X. (2015). Metabolomics profiling to investigate the pharmacologic mechanisms of berberine for the treatment of high-fat diet-induced nonalcoholic steatohepatitis. Evid. Based Complement. Altern. Med..

[234] Wang Y., Niu M., Jia G.L., Li R.S., Zhang Y.M., Zhang C.E., Meng Y.K., Cui H.R., Ma Z.J., Li D.H. (2016). Untargeted Metabolomics Reveals Intervention Effects of Total Turmeric Extract in a Rat Model of Nonalcoholic Fatty Liver Disease. Evid. Based Complement. Altern. Med..

[235] Van Pelt C.K., Huang M.C., Tschanz C.L., Brenna J.T. (1999). An octaene fatty acid, 4,7,10,13,16,19,22,25-octacosaoctaenoic acid (28:8n-3), found in marine oils. J. Lipid Res..

[236] Lu Y., Chen Y., Wu Y., Hao H., Liang W., Liu J., Huang R. (2019). Marine unsaturated fatty acids: Structures, bioactivities, biosynthesis and benefits. RSC Adv..

[237] Han J., Dzierlenga A.L., Lu Z., Billheimer D.D., Torabzadeh E., Lake A.D., Li H., Novak P., Shipkova P., Aranibar N. (2017). Metabolomic profiling distinction of human nonalcoholic fatty liver disease progression from a common rat model. Obesity (Silver Spring).

[238] Liu X.L., Ming Y.N., Zhang J.Y., Chen X.Y., Zeng M.D., Mao Y.M. (2017). Gene-metabolite network analysis in different nonalcoholic fatty liver disease phenotypes. Exp. Mol. Med..

[239] Maciejewska D., Palma J., Dec K., Skonieczna-Zydecka K., Gutowska I., Szczuko M., Jakubczyk K., Stachowska E. (2019). Is the Fatty Acids Profile in Blood a Good Predictor of Liver Changes? Correlation of Fatty Acids Profile with Fatty Acids Content in the Liver. Diagnostics.

[240] Tang Y., Zhang J., Li J., Lei X., Xu D., Wang Y., Li C., Li X., Mao Y. (2019). Turnover of bile acids in liver, serum and caecal content by high-fat diet feeding affects hepatic steatosis in rats. Biochim. Biophys. Acta Mol. Cell Biol. Lipids.

[241] Papandreou C., Bullo M., Tinahones F.J., Martinez-Gonzalez M.A., Corella D., Fragkiadakis G.A., Lopez-Miranda J., Estruch R., Fito M., Salas-Salvado J. (2017). Serum metabolites in non-alcoholic fatty-liver disease development or reversion; a targeted metabolomic approach within the PREDIMED trial. Nutr. Metab..

[242] Del Chierico F., Nobili V., Vernocchi P., Russo A., De Stefanis C., Gnani D., Furlanello C., Zandona A., Paci P., Capuani G. (2017). Gut microbiota profiling of pediatric nonalcoholic fatty liver disease and obese patients unveiled by an integrated meta-omics-based approach. Hepatology.

[243] Gawlik A., Shmoish M., Hartmann M.F., Wudy S.A., Olczak Z., Gruszczynska K., Hochberg Z. (2019). Steroid metabolomic signature of liver disease in nonsyndromic childhood obesity. Endocr. Connect..

[244] Robertson D.G. (2005). Metabonomics in toxicology: A review. Toxicol. Sci..

[245] Miao H., Zhao Y.H., Vaziri N.D., Tang D.D., Chen H., Chen H., Khazaeli M., Tarbiat-Boldaji M., Hatami L., Zhao Y.Y. (2016). Lipidomics Biomarkers of Diet-Induced Hyperlipidemia and Its Treatment with Poria cocos. J. Agric. Food Chem..

